# Indicators of the Statuses of Amphibian Populations and Their Potential for Exposure to Atrazine in Four Midwestern U.S. Conservation Areas

**DOI:** 10.1371/journal.pone.0107018

**Published:** 2014-09-12

**Authors:** Walt Sadinski, Mark Roth, Tyrone Hayes, Perry Jones, Alisa Gallant

**Affiliations:** 1 Upper Midwest Environmental Sciences Center, U.S. Geological Survey, La Crosse, Wisconsin, United States of America; 2 Laboratory for Integrative Studies in Amphibian Biology, Molecular Toxicology, Energy and Resources Group, Group in Endocrinology, Museum of Vertebrate Zoology, and Department of Integrative Biology, University of California, Berkeley, California, United States of America; 3 Minnesota Water Science Center, U.S. Geological Survey, Mounds View, Minnesota, United States of America; 4 Earth Resources Observation and Science Center, U.S. Geological Survey, Sioux Falls, South Dakota, United States of America; University of Sydney, Australia

## Abstract

Extensive corn production in the midwestern United States has physically eliminated or fragmented vast areas of historical amphibian habitat. Midwestern corn farmers also apply large quantities of fertilizers and herbicides, which can cause direct and indirect effects on amphibians. Limited field research regarding the statuses of midwestern amphibian populations near areas of corn production has left resource managers, conservation planners, and other stakeholders needing more information to improve conservation strategies and management plans. We repeatedly sampled amphibians in wetlands in four conservation areas along a gradient of proximity to corn production in Illinois, Iowa, Minnesota, and Wisconsin from 2002 to 2005 and estimated site occupancy. We measured frequencies of gross physical deformities in recent metamorphs and triazine concentrations in the water at breeding sites. We also measured trematode infection rates in kidneys of recently metamorphosed *Lithobates pipiens* collected from nine wetlands in 2003 and 2004. We detected all possible amphibian species in each study area. The amount of nearby row crops was limited in importance as a covariate for estimating site occupancy. We observed deformities in <5% of metamorphs sampled and proportions were not associated with triazine concentrations. Trematode infections were high in metamorphs from all sites we sampled, but not associated with site triazine concentrations, except perhaps for a subset of sites sampled in both years. We detected triazines more often and in higher concentrations in breeding wetlands closer to corn production. Triazine concentrations increased in floodplain wetlands as water levels rose after rainfall and were similar among lotic and lentic sites. Overall, our results suggest amphibian populations were not faring differently among these four conservation areas, regardless of their proximity to corn production, and that the ecological dynamics of atrazine exposure were complex.

## Introduction

Large-scale agriculture is a principal driver of amphibian population declines [1]–[3] primarily for two reasons: historical and ongoing conversion of native land cover to agricultural production has caused the loss and fragmentation of amphibian habitat in many regions worldwide (e.g., [4]–[7]) and amphibians are susceptible to direct and indirect effects on fitness from potentially eutrophying or toxic agricultural chemicals released into the environment to enhance agricultural production (e.g., [4], [8]–[10]). Parts of the midwestern United States are among the most intensively farmed areas in the world [6]–[7]. Midwestern farmers annually produce the majority of corn (Fig. S1) and soybeans in the United States, as well as lesser quantities of barley, oats, wheat, beans, hay, apples, pork, dairy products, beef, and other commodities [11]. As has been described similarly for other areas [12], conversion of native land cover to agricultural production has caused broad-scale loss and fragmentation of midwestern wildlife habitat with direct ramifications for amphibians, particularly in the corn belt across Minnesota, Iowa, Illinois, Indiana, and Ohio. In Iowa, for example, farmers reduced native grasslands from approximately 80% of the state's land area in the mid-1800s to about 5% by 2001 and drained approximately 96% of the historical wetland surface area in the Des Moines Lobe, the southernmost part of the Prairie Pothole Region, over a similar time span [7]. Comparable changes in land cover and use also occurred across areas of other midwestern states, resulting in the extensive loss and fragmentation of habitat for amphibians and other species since European settlers began farming the Midwest's rich soils [4].

Agricultural chemicals applied to the landscape can degrade remaining native habitat and affect organisms in various ways. Manure and industrial sources of nitrogen and phosphorous applied extensively as fertilizers in the Midwest (Fig. S2) can cause eutrophication of wetlands locally and regionally [4] and also drive hypoxia in the Gulf of Mexico (e.g., [13]). Herbicides and other pesticides applied throughout this region can cause direct and indirect effects on aquatic biodiversity [4]. Atrazine, a triazine herbicide used to control weeds and grasses in corn fields primarily [14], has been of particular concern [8], [15], especially given the quantities of this compound farmers have applied annually ([16]; Fig. S3). Atrazine reportedly can cause a range of effects on amphibians and other wildlife, including direct endocrine disruption and increased trematode infections mediated indirectly via food-web linkages (see [4], [8], [15], [17]–[19], and [20] for reviews and analyses and Text S1 for details pertaining specifically to the Midwest). However, most of the concern and evidence for atrazine's effects have come from controlled studies in laboratories and mesocosms, leaving considerable uncertainty regarding the extent of effects on wild populations [8], [20].

Recent increased corn production (Fig. S4), associated with economic incentives and mandates to produce more corn for biofuels [21]–[23], likely has further reduced the quantity and quality of midwestern amphibian habitat and underscores the need for rigorous baseline information on regional amphibian populations and their habitats [24]–[25]. Yet, we know of no reported data from field sampling across years, areas, and sites that describe species diversity and site occupancy for midwestern amphibian species in relation to the proximity of corn production or their potential for exposure to atrazine at breeding sites.

State-coordinated nighttime call surveys of frogs and toads have produced most of the information available on the long-term, broad-scale occurrence of amphibian species that call in the Midwest [26]–[30]. Results from these programs suggest reduced occurrence for some species in or near agricultural areas in recent years [26]–[30], during which occurrence was relatively stable for most species and appeared to increase for others (e.g., [30]). Such results are useful for evaluating species presence broadly across geographic areas [25]. However, interpreting them more specifically in terms of population statuses and specific habitat conditions can be challenging because of environmental variability these surveys do not address [25] and limitations of the sampling protocols often used [31]. For example, when they are available, volunteers of varying experience typically survey calling amphibians from roads or other easy-access points at night and may have little knowledge of a specific wetland from which amphibians are calling. Also, these surveys usually do not include characterizing habitat conditions (but see [26]–[27]) or measuring indicators of the health of any amphibians surveyed. Thus, the resultant data can be limited for understanding site occupancy and habitat conditions relative to corn production.

This overall lack of useful information limits the ability of resource managers, scientists, and regulators to assess the statuses of amphibian populations relative to risks associated with corn and other agricultural production. This is true even in protected conservation areas where managers have ranked ecological effects of agricultural land use high on their list of concerns (e.g., [32]). Researchers and regulators also lack critical information for broader applications. For example, they mostly have to use atrazine concentrations measured in streams, rivers, and large reservoirs as part of national water-quality assessment programs to evaluate exposure risks amphibians face in the wild [20], [33] and the relevance of concentrations used in experimental tests of atrazine's effects on amphibians [15], [20]. Flowing water and large reservoirs are not suitable breeding habitat for most U.S. amphibian species [34], which could limit the relevance of this overall approach given the likelihood of different fate-and-transport dynamics in and around smaller lentic wetlands [15], [20] in which most species breed.

To help address such data deficiencies, we report here on our research conducted on lands managed by the U.S. Department of Interior in parts of four midwestern states. Our study areas included two national wildlife refuges, a national park, and a national scenic riverway located along a gradient of proximity to intensive corn production in Iowa, Illinois, Minnesota, and Wisconsin. Land cover within the boundaries of these areas was protected from conversion to corn production, but amphibian populations within and overlapping with each area hypothetically still were vulnerable to effects from corn production in relation to the distance, and the atmospheric and hydrologic connectivity, to such production. Our general objectives were to measure the proximity and extent of corn production relative to our study areas, the presence of individual amphibian species relative to those likely present, the frequency of gross physical deformities, and triazine concentrations in breeding wetlands for each area (atrazine often is the dominant triazine found in the environment in this region [35]). We also set out to model site occupancy for several amphibian species relative to the amount of nearby cropland and other land cover, measure frequencies of trematode infections in *Lithobates pipiens* metamorphs from a sample of wetlands, measure triazine concentrations in water samples from lotic non-breeding sites in the floodplain of the Mississippi River to compare with nearby lentic breeding sites, and to qualitatively evaluate how climate and farming practices might influence variation in atrazine use relative to the timing of amphibian reproduction. Our results provide relatively unique information regarding the statuses of amphibian populations along this gradient of corn production and their potential to be exposed to atrazine. All of this work was in support of the U.S. Geological Survey's Amphibian Research and Monitoring Initiative [36]–[37]. The surveys for amphibians in the St. Croix National Scenic Riverway and Voyageurs National Park also were in support of the U.S. National Park Service's Inventory and Monitoring Program [38].

## Methods

### 1. Ethics Statement

We handled and euthanized all animals humanely under the conditions of a permit issued by the Upper Midwest Environmental Sciences Center's Animal Care and Use Committee (to WS) and in accordance with Animal Use Protocol R209–011BRC issued by the University of California, Berkeley (to TH). None of this research involved endangered or protected species.

### 2. Study Areas

**Figure 1:**
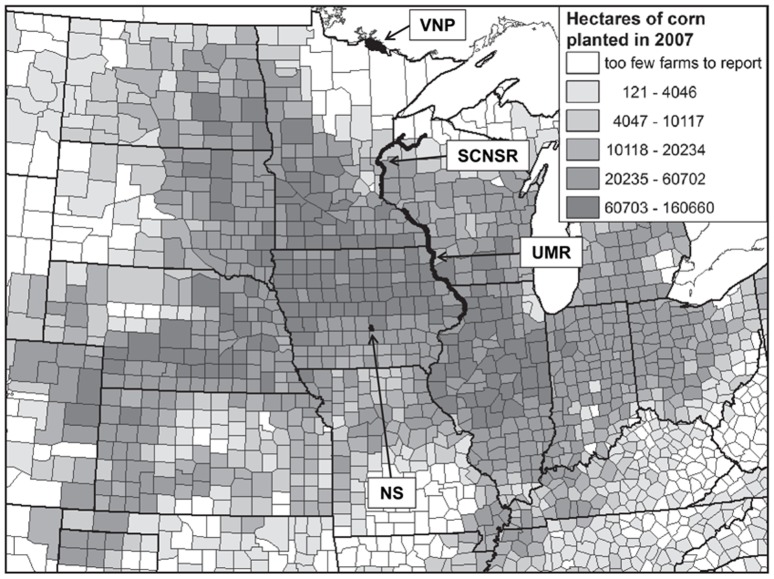
Location of study areas relative to areas of intensive corn production. NS  =  the Neal Smith National Wildlife Refuge; UMR  =  the Upper Mississippi River National Wildlife and Fish Refuge; SCNSR  =  the St. Croix National Scenic Riverway; VNP  =  Voyageurs National Park. Gray tones across counties indicate total number of ha planted in corn in 2007 [41], rendered via classes representing natural breaks in the data distribution.

We conducted field work in the Neal Smith National Wildlife Refuge (NS), the Upper Mississippi River National Wildlife and Fish Refuge (UMR), the St. Croix National Scenic Riverway (SCNSR), and Voyageurs National Park (VNP). These four conservation areas lie along a broad north-south gradient of climate, land cover, land use, habitat types, amphibian diversity, and proximity to intensive corn production (Fig. 1). The NS (2,172 ha) is in central Iowa. The UMR (97,125 ha) is centered along approximately 420 km of the Mississippi River floodplain in Minnesota, Wisconsin, Iowa, and Illinois. The SCNSR (37,530 ha) is centered along approximately 405 km of the St. Croix and Namekagon Rivers in Wisconsin and Minnesota. A portion of VNP (88,244 ha) forms approximately 85 km of the international border between Minnesota and Ontario, Canada. The NS, UMR, SCNSR, and VNP are in Omernik Level-III Ecoregions 47; 47, 52, and 54; 50 and 51; and 50, respectively [39]–[40] (Table S1), which was reflected in differences in land cover and other features among these areas (Fig. S5). The NS and the UMR are public lands managed by the U.S. Fish and Wildlife Service (FWS). The SCNSR and VNP are public lands managed by the U.S. National Park Service (NPS). The FWS and NPS provided the permits (to WS) necessary for us to work in these areas.

### 3. Proximity of Study Areas to Intensive Corn Production

To establish the relative proximity of each study area to intensive corn production, we estimated land planted in corn from annual cropland maps developed by the U.S. Department of Agriculture's National Agricultural Statistics Service based upon remotely sensed data collected in 2008 by the Advanced Wide Field Sensor [41]. This was the earliest data set available for crop types across all states containing our study areas and likely described land use similar to 2002 to 2005 based on typical land use and crop rotations in these areas. Metadata for the crop maps described mapping accuracies for corn as >97% in Iowa, Illinois, and Minnesota and approximately 93% in Wisconsin. We calculated the proportion of pixels in corn crops from within one km of the boundaries of each study area outward to 512 km, doubling the distance for each successive buffer and estimate. This range encompassed distances reported for aerial transport of atrazine [15].

### 4. Selection of Study Wetlands

We selected all study wetlands described in this report, except the water-collection sites in the main channel of the Mississippi River, based upon their potential to be amphibian breeding sites. We used a geographic information system to select sites for daytime amphibian sampling, which included a grid of 25-ha square cells placed over the area of inference in each individual study area. We selected cells randomly from this grid in each study area, then surveyed each selected cell on the ground to identify individual study wetlands. See Text S2 for further details.

### 5. Amphibian Surveys

#### 5.1. Sites sampled per management area and site characteristics

We surveyed wetlands for amphibians in the NS in 2004 and 2005 and in the UMR, the SCNSR, and VNP from 2002 to 2005. We surveyed a total of 17, 73, 64, and 57 individual wetlands across years in the NS, the UMR, the SCNSR, and VNP, respectively. We did not survey all wetlands for amphibians in all years.

We evaluated the hydroperiod of each wetland we surveyed as ephemeral, semi-permanent, or permanent based upon site visits across seasons. We measured pH and conductivity for each wetland just below the surface in 20 to 40 cm of water at one location selected haphazardly during each sampling occasion. We used either Hanna (Woonsocket, RI, USA) Model HI991300 portable waterproof field meters or Oakton (Vernon Hills, IL, USA) Model PC10 portable waterproof field meters for these measurements according to the manufacturers' recommendations.

#### 5.2. Sampling frequency

Our intent was to sample most breeding sites during daytime once during each of the early (late March to early May), middle (mid-May to late June), and late (late June to early August) intervals of the amphibian breeding season in each study area. This enabled us to observe the presence of various life stages of all species that might breed and be present in a wetland over the course of the season. However, we were not always able to sample all sites on three separate occasions in each year due to wetlands drying, time constraints, and other unforeseen circumstances (Table S2). Two persons typically surveyed a site on each sampling occasion by working independently but according to the same protocols. We treated each person's survey on each occasion as an independent survey event for that site.

#### 5.3. Daytime surveys

Two surveyors independently measured presence/absence of species per wetland via call, visual-encounter, and dip-net surveys, and also surveyed for metamorphs later in the season, during each site visit. We conducted less than two surveys per method per site visit when conditions did not allow us to do so, such as small wetland size, insufficient water or larval habitat, or the site visit being too early for metamorphs. Each surveyor measured presence/absence and recorded data without communication with the other surveyor.

We conducted daytime call surveys by listening with the naked ear for ten minutes from a location ten to 30 m away from the wetland, hidden from view when possible to reduce the likelihood of interrupting calling or breeding. We began surveying after waiting quietly for five minutes to allow any interrupted amphibians to resume calling.

We conducted time-constrained visual-encounter (VES) and dip-net surveys, using the naked eye and dip nets, respectively, immediately after completing call surveys. During the VES portion, individual surveyors walked slowly along a haphazardly chosen transect through potential amphibian habitat in each wetland for 20 minutes and visually surveyed for all life stages of all possible species. During the dip-net portion, each surveyor took one sweep with a dip net at up to ten nodes along the VES transect, depending upon the size of the wetland and the quantity of appropriate habitat to sample. Nodes were two minutes of walking time apart from the beginning of the VES transect or from the previous node. Time allotted for each VES was suspended (stopwatch) at each node while we sampled via dip nets and identified and recorded amphibians collected in the sweep.

During site visits later in the season and after VES and dip-net surveys were completed, each surveyor sampled separate appropriate habitat adjacent to the breeding site for recent metamorphs and captured as many metamorphs as possible by hand and with nets during a 20-minute period. We surveyed for metamorphs when we expected recent metamorphs to be present based upon earlier breeding activity of species at sites. We did not monitor larval development and environmental conditions closely at individual breeding sites over the course of the season. Therefore, our metamorph surveys were opportunistic within a range of time per season and with regard to which species we sampled at a site in a given year.

#### 5.4. Nighttime call surveys

We primarily used parabolic microphones (Telinga Pro 5, Pro 6; Tobo, Sweden) connected to mini-disc recorders (HHB MDP500; HHB Communications USA; Los Angeles, CA, USA) to conduct nighttime call surveys for the presence of amphibian species from listening locations along roads. We picked these locations non-randomly from 2002 to 2005 in VNP, and from 2002 to 2004 in the SCNSR and the UMR (Table S3), to be near wetlands we sampled during the day and, thus, to complement our daytime sampling. From 2004 to 2005, we did the same for the NS. In 2005, we selected individual sites randomly from a pool of potential listening locations along roads in the SCNSR and the UMR, regardless of their proximity to our daytime sampling sites. We typically conducted nighttime call surveys once per listening location in each management area during the dates we conducted daytime surveys in the same area. Given the extensive and condition-dependent reach of the parabolic microphones and the non-random process we used for selecting listening locations in some cases, our areas of inference for the nighttime surveys were not well-defined.

### 6. Data Analyses for Amphibian Surveys

#### 6.1. Naïve occupancy estimates

We calculated naïve occupancy estimates (number of surveyed sites where species x was detected/number of sites surveyed) based upon results from daytime and nighttime surveys. In calculating these raw estimates, we did not adjust the number of sites surveyed to account for the likely habitat suitability of each wetland for individual species.

#### 6.2. Occupancy models

We were not able to model site occupancy based upon our nighttime call-survey data due to small numbers of sites in some years and changes in sampling protocols and locations in some areas across years. We had sufficient numbers of sites, repeat surveys, and detections from our daytime surveys to model site occupancy for eight species across areas and years (NS: 2004–2005; UMR, the SCNSR, and VNP: 2002 to 2005). We used the multi-year option [42]–[46] in the computer program, PRESENCE [47], to estimate model parameters for each combination of species and study area. We only included sites for each species that likely were suitable habitat based upon our knowledge of each species' distribution and life-history traits and assessments of each site in the field. We did not analyze data for any rare or seldom detected species. Thus, depending upon the study area, we modeled occupancy for *Anaxyrus americanus* (American toads), *Hyla versicolor/chrysoscelis* (eastern and gray treefrogs collectively, which we could not distinguish visually), *Pseudacris crucifer* (spring peepers), *P. maculata* (boreal chorus frogs), *L. clamitans* (green frogs), *L. pipiens* (northern leopard frogs), *L. septentrionalis* (mink frogs), and *L. sylvaticus* (wood frogs).

We hypothesized *a priori* which habitat and landscape characteristics might influence occupancy (ψ) and detection (ρ) probabilities for each species and tested their influence on our estimates. We used site hydroperiod (ephemeral, semi-permanent, permanent) and three landscape variables based on land cover (described below) within a 4-km radius (buffer) of each site as covariates on ψ and included each covariate singly and in all possible combinations. We selected this buffer size based upon known life-history traits of our study species that included use of upland habitats and dispersal capabilities [34]. This buffer also is well within distances atrazine has been reported to travel via atmospheric transport [15]. We derived values for the three landscape variables through geospatial analyses of land-cover data sets. We calculated 1) the percentage of the buffer in cultivated cropland (% crops), 2) percentage of the buffer in non-cultivated-cropland cover types that likely were not suitable as amphibian habitat (urban/suburban, roads, and quarries/mines; % not habitat), and 3) the area-weighted mean size of the patches of the remaining (after 1 and 2 were removed) land-cover types in the buffer that were suitable as potential amphibian habitat (mean patch size of habitat). These three variables conveyed information about the proportion of the local landscape that likely was planted in cropland, the proportion that likely was not habitat, and the degree of coherence or fragmentation of the proportion that potentially was habitat, respectively. See Text S3 for more details regarding our data sources and how we measured % crops, % not habitat, and mean patch size of habitat.

We also modeled observer (novice or experienced) and sampling method (call survey, VES, dip-net survey, metamorph survey) as covariates on ρ, but did not allow them to vary by model, except in the case of the null model (no covariates on any model parameter). In combination with the covariates we associated with ψ, this resulted in 17 possible models (including the null model). Note that we were not able to include triazine concentration as a covariate on any model parameter due to insufficient numbers of repeat samples for triazines across the sites we surveyed and limited detections among such samples.

We considered any model from PRESENCE outputs that met the following four criteria: 1) no estimates of coefficients for model parameters >|5|; 2) no estimates of the standard error for coefficients for model parameters >5× |estimate of the corresponding parameter coefficient|; 3) no estimates of 0 for coefficients for model parameters or for estimates of the standard errors for such coefficients; and 4) no warning in the output suggesting non-convergence described as >4 significant digits. Of the models that met these criteria, we further considered only those models with ΔAIC (Akaike's Information Criterion) values of ≤ 5 compared with the AIC value of the highest ranked model. Burnham and Anderson [48] suggested that models with ΔAIC values of 0–2 have “substantial” empirical support, whereas models with ΔAIC values of 4–7 have “considerably less” empirical support and models with a ΔAIC value>10 have essentially no empirical support. We selected a maximum ΔAIC value of five to report results from a potentially relevant and reasonable set of models for each species.

We assessed the relative impact of a specific covariate on the estimate of its associated model parameter by summing the AIC weights across the set of individual models (≤ 5 ΔAIC for each species) that included that specific covariate as important [46].

### 7. Surveys and Analyses for Gross External Deformities

We used containers to sequester individual recent metamorphs (tails fully resorbed) of all species captured during the metamorph surveys described earlier. Upon completion of the metamorph survey, we assessed each individual for gross external deformities using the naked eye according to Meteyer et al. [49]. When recent metamorphs of a species were present at a site during more than one metamorph survey, we only considered measurements from the survey with the largest number of individuals to avoid resampling individuals. We report here on results from surveys at all sites we sampled for deformities, regardless of whether we sampled them for triazines. The numbers of sites we sampled for gross deformities per study area were eight unique sites in the NS from 2004 to 2005; 57 unique sites in the UMR from 2002 to 2005; 58 unique sites in the SCNSR from 2002 to 2005; and 51 unique sites in VNP from 2002 to 2005 (Table S4). Our exploratory analyses of the resultant data suggested measurements of deformities across sites and years likely were not related to triazine concentrations at those sites we had sampled for triazines. However, we also tested for an association for sites where we measured both variables in 2004 and 2005 via the Spearman's Rank Test in Origin Pro software (v. 9.1; Origin Lab; Northampton, MA, USA).

### 8. Collection of Metamorphs for Trematode Analyses

We collected 100 to 105 recently metamorphosed (tails fully resorbed) *L. pipiens* from each of seven wetlands (three in the UMR, two in the SCNSR, and two in VNP) in 2003 for analyses of trematode infections. In 2004, we collected 51 to 100 *L. pipiens* metamorphs from five of these same seven sites. We also collected metamorphs from two new sites (wetlands distributed among the study areas similar to 2003) because of insufficient numbers of metamorphs at two of the sites we sampled in 2003. We measured gross physical abnormalities in all *L. pipiens* metamorphs we collected for these analyses. We also measured triazine concentrations in water samples we collected from these wetlands in both years.

We shipped live animals collected from site P4DD1 in 2004 to the University of California, Berkeley (UCB), for fixing, preserving, and trematode analyses. We used MS-222 (250 mg/L) to euthanize all other metamorphs we collected, fixed them in Bouin's Solution, and preserved them in a 70% solution of EtOH at the U.S. Geological Survey's (USGS) Upper Midwest Environmental Sciences Center (UMESC) in La Crosse, Wisconsin, prior to shipment to UCB. We shipped 50 to 54 haphazardly selected specimens from each set of specimens collected at each wetland to UCB for analyses. We kept the remaining specimens from each site at UMESC in case shipments were lost or damaged. All sets of metamorphs collected from each site were given information-neutral numeric codes prior to shipment, such that researchers from UCB did not know the identity of the sites from which specimens were collected or anything about their triazine concentrations beforehand.

### 9. Specimen Preparation for Trematode Analyses

At UCB, we sampled specimens for analysis haphazardly from the coded, site-specific storage jars shipped from UMESC and conducted all assessments of trematode infections blindly. Each such specimen was measured for snout-vent length and examined for any gross external abnormalities, such as limb deformities and macroparasites. We then examined gonads through an abdominal incision using a Nikon SMZ 10A dissecting scope (Technical Instruments; Burlingame, CA, USA) to identify males. We removed kidneys from 20 haphazardly selected males from each site (except for four sites where there were fewer than 20 males among the 50 animals examined), dehydrated them in degraded alcohols and infiltrated them with Histoclear (National Diagnostics; Atlanta, GA, USA) and paraffin, then cut them into serial histological transverse sections at 8 µm with a rotary microtome and mounted them on slides. We stained slides in hematoxilin:eosin, and analyzed them using a Nikon Optiphot 2 microscope (Technical Instruments; Burlingame, CA, USA). We used a Nikon Digital Sight DS–U (Technical Instruments; Burlingame, CA, USA) and Scion Image (Scion Corporation; Frederick, MD, USA) to capture photomicrographs and NIS Freeware 2.1 (NIS Elements; Melville, NY, USA) to analyze morphometry.

### 10. Measurement of Trematode (Echinostome) Infections and Test for Association with Triazines Concentrations

We measured trematode-infection rates at UCB by counting the number of trematode (echinostome) cysts in both kidneys. We identified the section with the maximum number of echinostomes and counted the echinostomes in the largest section of the kidney. We distinguished live and dead echinostomes based on the appearance of the individual cysts and whether they were encapsulated in the kidney and calculated mean infection rates using all males analyzed and using only males with infections. Exploratory analyses of the resultant data suggested no likely relations between our measurements of echinostomes in males collected across sites and years and triazine concentrations at those sites, but we tested for any associations via the Spearman's Rank Test in the Origin Pro software described earlier.

### 11. Water Sampling and Analyses for Triazines

#### 11.1. Sample collection for enzyme-linked immunosorbent assays

We collected water samples from seven and 27 wetlands in the NS in 2004 and 2005, respectively. In 2003, 2004, and 2005, we collected water samples from wetlands in the UMR (five, 60, 38, respectively), SCNSR (four, 53, 18, respectively), and VNP (five, 46, 24, respectively). These wetlands were a subset of the wetlands we sampled for amphibians in each study area, except for 14 lotic sites in the main channel of the Mississippi River (UMR), which we sampled just for triazines in 2004 and 2005. These channel sites were directly outward from the lentic amphibian breeding sites (slightly higher in elevation) we sampled for amphibians and triazines in Pools 4, 7, 8, 10, 13, and 14 of the floodplain and were not amphibian breeding habitat. We sampled these channel locations to compare triazine concentrations in the flowing river with those in the non-flowing breeding sites. All these sites could have become connected across the surface during high water. We also collected water samples from channel sites and known amphibian breeding sites in Pool 11 of the UMR. However, we did not survey these latter sites for amphibians as part of this study.

We selected wetlands for collecting water samples each year based upon the set of all sites we surveyed for amphibians, logistical considerations, available resources, and the presence of sufficient standing water. Water samples were not necessarily collected from the same sites in all years. We typically sampled sites once during 2003 and one to three times per site per year during 2004 and 2005, except in the UMR during 2004 and 2005, when we sampled specific sites up to six (2005) or seven (2004) times to obtain finer-scaled spatial and temporal data regarding triazine concentrations.

We collected unfiltered water samples from just below the surface of the water at one shallow location per breeding site or over unknown depths in the main channel of the Mississippi River. One person wearing new latex gloves filled and capped a sterile glass sample bottle while the bottle was completely submerged. Samples were stored unfrozen in ice-filled coolers while in the field and at 4°C in the laboratory prior to analyses.

#### 11.2. Enzyme-linked immunosorbent assays

We analyzed water samples at UMESC via an enzyme-linked immunosorbent assay (ELISA) for atrazine (Atrazine Magnetic Particle Kit; Abraxis Ltd.; Warminster, PA, USA) according to the manufacturer's recommended protocols using their reagents and a Thermo Scientific spectrophotometer (Model Genesys 6; Waltham, MA, USA). The antibodies in the ELISA kits were designed to react specifically with atrazine (minimal detection limit = 0.05 ppb), but they also could have reacted with additional triazines (propazine, ametryn, prometryn, prometon, desethyl atrazine, terbutryn, simazine, desisopropyl atrazine, cyanazine, and 2-hydroxy atrazine) when such compounds were present according to information provided by the manufacturer.

#### 11.3. Sample collection for comparison of ELISA with liquid chromatography/mass spectrometry

We collected water samples in 2003 from three sites in the UMR, two sites in the SCNSR, and three sites in VNP to compare concentrations of atrazine and other triazines at the same sites measured via the ELISA and measured via solid-phase extraction and liquid chromatography/mass spectrometry (LCMS). We collected water samples from 12 other sites in the UMR in 2004 and from 11 of these same 12 sites again in 2005 for the same purpose. We collected all water samples for these analyses according to USGS sampling protocols [50]. We collected three water samples sequentially per site just below the water's surface at the same location. We filtered two of these samples using a peristaltic pump to draw sufficient water through 0.7-µm glass-fiber filters to remove suspended particulate matter that might have bound dissolved triazines during storage.

#### 11.4. ELISA and LCMS analyses for triazine herbicides

We analyzed the unfiltered sample and one of the filtered samples via the ELISA at UMESC and sent the other filtered sample to the USGS's Organic Geochemistry Research Group in Lawrence, Kansas, for analyses of triazines, including atrazine, and their degradation products. The analytical methods and procedures using solid-phase extraction and LCMS are described in Lee et al. [51]. Note that of the triazines that potentially cross-reacted with the atrazine antibodies using the ELISA, we did not analyze for ametryn, prometryn, and terbutryn via LCMS.

## Results

### 1. Proximity of Study Areas to Intensive Corn Production

**Figure 2:**
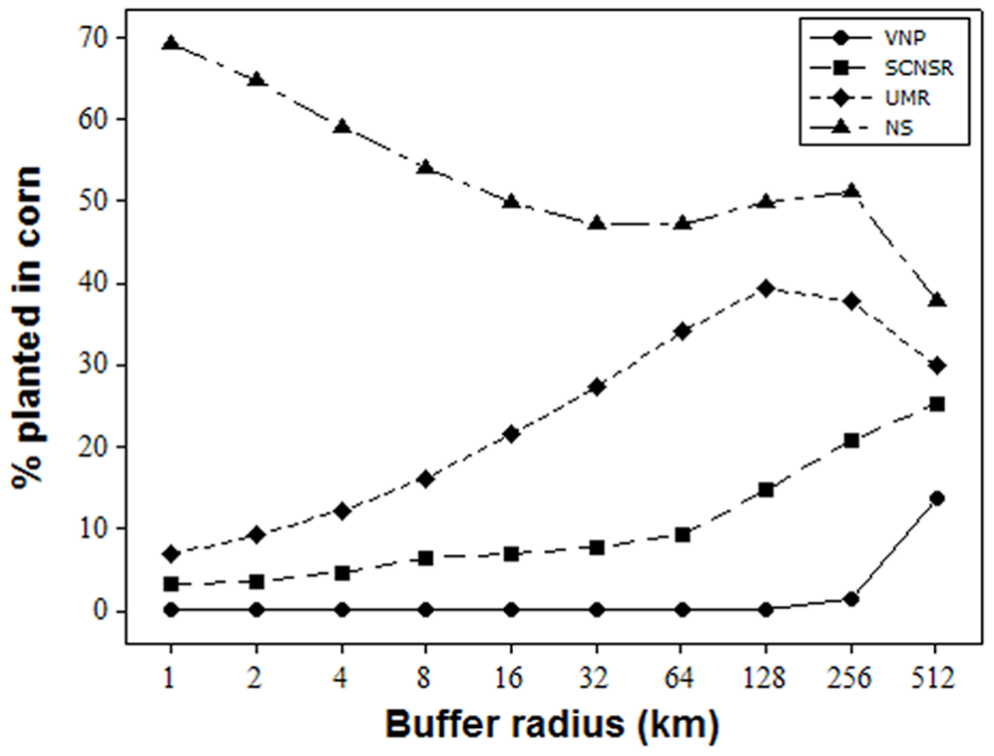
Percent of the surrounding landscape planted in corn within successive buffers outwards from each study area during 2008. NS  =  the Neal Smith National Wildlife Refuge; UMR  =  the Upper Mississippi River National Wildlife and Fish Refuge; SCNSR  =  the St. Croix National Scenic Riverway; VNP  =  Voyageurs National Park.

A greater proportion of the landscape surrounding the NS was planted in corn than the proportions surrounding the UMR and the SCNSR (Fig. 2). Essentially none of the landscape surrounding VNP was planted in corn until we considered distances greater than 256 km. The ranking of study areas based on the proportion of surrounding landscape planted in corn was consistent (NS > UMR > SCNSR > VNP) regardless of distance, although proportions converged with increasing distance (Fig. 2).

### 2. Site Characteristics

#### 2.1. Wetland pHs and conductivities

Analytical results (via one-sample sign tests; Minitab v.16; State College, PA) of measurements *in situ* showed pHs were lowest in VNP (n = 64; median = 5.80; 95% confidence interval [c.i.] = 5.53, 6.085) and highest in the UMR (n = 62; median = 7.36; 95% c.i. = 7.22, 7.48). Values for the SCNSR (n = 76; median = 6.29; 95% c.i. = 5.99, 6.67) and the NS (n = 22; median = 6.95; 95% c.i. = 6.509, 7.801) were intermediate. Conductivity showed a similar pattern, with values in VNP lowest (n = 63; median = 56.0 µS/cm; 95% c.i. = 48.9, 67.6) and those in the UMR highest (n = 62; median = 406.8 µS/cm; 95% c.i. = 386, 438). Values for the SCNSR (n = 77; median = 112 µS/cm; 95% c.i. = 67.6, 123) and the NS (n = 22; median = 283 µS/cm; 95% c.i. = 237, 360) were intermediate.

### 3. Amphibian Surveys

We detected 75, 72, 89, and 71% of the amphibian species potentially present in the NS, UMR, SCNSR, and VNP, respectively, based upon information we obtained from field guides (e.g., [52]–[53]), reference books (e.g., [34]), and NPS and FWS biologists. We concluded that we had little chance of detecting the species we did not observe in each conservation area because our sampling methods (e.g., *Plethodon cinereus* in the SCNSR and VNP and *Hemidactylum scutatum* in the UMR and the SCNSR), the habitat types we sampled (e.g., *A. tigrinum* in the UMR and the SCNSR and *L. palustris* in the SCNSR), or the likelihood that a species actually occurred there (e.g., *A. maculatum* in the UMR and *L. catesbeiana* in VNP) were preclusive. *Ambystoma texanum* was the lone exception to this in the NS. Thus, from a practical perspective, all the species projected beforehand to potentially inhabit our study areas and for which we surveyed were present, except one.

#### 3.1. Naïve occupancy estimates

The proportions of sites where we detected individual species varied within and across study areas and across years (Tables 1, 2). Daytime surveys enabled us to observe salamander species that do not call and nighttime call surveys enabled us to detect *L*. *palustris* (pickerel frog), a relatively uncommon species in the UMR. We also assumed we were able to distinguish between *H. versicolor* and *H. chrysoscelis* via call surveys, which we could not do via visual surveys.

**Table 1:** Naïve estimates (number of sites where we observed each species relative to the total number of sites we surveyed) from daytime surveys conducted during 2002 to 2005.

Management Area	*Notophthalmus viridescens louisianensis*	*Ambystoma maculatum*	*A. laterale*	*A. tigrinum*	*Anaxyrus americanus*	*Acris crepitans*	*Hyla chrysoscelis* 1	*H. chrysoscelis/versicolor* 2	*Pseudacris crucifer*	*P. maculata*	*Lithobates blairi*	*L. catesbeiana*	*L. clamitans*	*L. palustris*	*L. pipiens*	*L. septentrionalis*	*L. sylvaticus*
NS	-	-	-	3/17	7/17	11/17	3/17	6/17	0/17^3^	16/17	1/17	6/17	0/17^3^	-	9/17	-	-
UMR	1/73	0/73	4/73	0/73	14/73	13/73	15/73	34/73	13/73	6/73	-	44/73	49/73	0/73	58/73	-	3/73
SCNSR	1/64	3/64	18/64	0/64	34/64	-	17/64	32/64	29/64	25/64	-	1/64	57/64	0/64^3^	40/64	16/64	46/64
VNP	8/57	-	17/57	-	21/57	-	-	20/57	31/57	9/57	-	0/57	23/57	-	33/57	15/57	52/57

NS  =  the Neal Smith National Wildlife Refuge from 2004 to 2005; UMR  =  the Upper Mississippi National Wildlife and Fish Refuge from 2002 to 2005; SCNSR  =  the St. Croix National Scenic Riverway from 2002 to 2005; VNP  =  Voyageurs National Park from 2002 to 2005. Dashes indicate management areas outside of the described range for that species.

1 The ranges of *Hyla chrysoscelis and H. versicolor* overlapped and they were indistinguishable visually, but we assumed we could distinguish their calls [34]. The detections listed in this column are based upon the numbers of sites where we heard *H. chrysoscelis* call.

2 The combined totals of sites where we detected *H. chryoscelis* via calls, sites where we detected *H. versicolor* via calls, and sites where we detected either *H. chrysoscelis* or *versicolor* visually, but could not distinguish between them. Thus, the numbers of sites with detections listed in the previous column, *Hyla chrysoscelis*, are included in the totals here.

3 This management area was very close to the edge of the described range of this species. We did not detect it there.

Note: We also found *Necturus maculosus*, *Hemidactylium scutatum*, and *Plethodon cinereus* in the SCNSR during small numbers of targeted searches.

**Table 2:** Naïve estimates (number of sites where we observed each species relative to the total number of sites we surveyed) from nighttime call surveys during 2002 to 2005.

Management Area	*Anaxyrus americanus*	*Acris crepitans*	*Hyla chrysoscelis*	*H. versicolor*	*Pseudacris crucifer*	*P. maculata*	*Lithobates catesbeiana*	*L. clamitans*	*L. palustris*	*L. pipiens*	*L. septentrionalis*	*L. sylvaticus*
NS	7/7	7/7	7/7	7/7	0/7^1^	7/7	7/7	0/7^1^	-	7/7	-	-
UMR	6/8	3/8	6/8	6/8	5/8	4/8	5/8	8/8	1/8	6/8	-	3/8
UMR 2005	22/34	10/34	4/34	28/34	15/34	11/34	15/34	25/34	4/34	13/34	-	0/34
SCNSR	8/10	-	4/10	8/10	10/10	6/10	1/10	7/10	0/10^1^	5/10	2/10	7/10
SCNSR 2005	28/36	-	4/36	35/36	21/36	19/36	3/36	25/36	0/36^1^	9/36	5/36	8/36
VNP	3/4	-	-	4/4	4/4	1/4	0/4	4/4	-	4/4	4/4	4/4

NS  =  the Neal Smith National Wildlife Refuge from 2004 to 2005; UMR  =  the Upper Mississippi National Wildlife and Fish Refuge from 2002 to 2004 and 2005 (when we changed sampling locations from the previous years); SCNSR  =  the St. Croix National Scenic Riverway from 2002 to 2004 and 2005 (when we changed sampling locations from the previous years); VNP  =  Voyageurs National Park from 2002 to 2005. Dashes indicate management areas outside of the described range for that species.

1 This management area was very close to the edge of the described range of this species. We did not detect it there.

### 4. Occupancy Estimates

We report key general outcomes here regarding estimates of occupancy and detection probabilities for species across study areas and the relative values of covariates in the top models. See Text S4 for outcomes more specific to each study area.

#### 4.1. General outcomes

We modeled occupancy for two species in the NS, four in the UMR, seven in the SCNSR, and seven in VNP (Tables 3, 4, 5, 6). The median number of models that were ≤ 5 ΔAIC and that met our other acceptance criteria (top models) across species and study areas was two (rounded up), with a range of one to six (Tables S5–8). Null models were among the models that were ≤ 5 ΔAIC for seven of the 20 species/study area combinations (Tables S5–8), indicating covariates did not improve those models much or at all. As expected, estimates of occupancy and detection probabilities varied by species and study area (Tables 3, 4, 5, 6). Generally, estimates of ψ and ρ were higher for *Lithobates* species across study areas than for the other species (Tables 3, 4, 5, 6) and standard errors associated with estimates of ψ generally precluded clear species-specific comparisons of occupancy across years. Across the overall relatively limited number of top models (Tables S5–8), % crops was important (based upon the sum of AIC weights across top models; Tables S5–12) for estimating ψ for *L. pipiens* in the UMR (Table S10) and for *A. americanus* in the SCNSR (Table S11) and somewhat less important for estimating ψ for *L. pipiens* in the SCNSR (Table S11), but not for any other combinations of species and study area (Tables S5–12). Mean patch size of habitat was the covariate on ψ that occurred most often in the top models across species and study areas (Tables S5–12) and notably was important for *L. pipiens* in the UMR (Table S10) and *P. crucifer* and *L. pipiens* in the SCNSR (Table S11). Hydroperiod was important for estimating ψ for *L. pipiens* in the UMR (Table S10) and *P. crucifer* in VNP (Table S12). Observer and method were important covariates for estimating ρ for all species and study areas (Tables S5–12), except for *L. clamitans* in the UMR (Table S10), *P. maculata* and *L. septentrionalis* in the SCNSR (Table S11), and *L. sylvaticus* in VNP (Table S12).

**Table 3:** Estimated values for parameters from site occupancy models of data collected via daytime surveys in the Neal Smith National Wildlife Refuge from 2004 to 2005.

*Species*		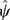 2	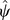	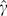 4	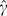	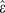 5	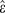	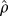 6	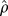
Year	( 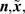 IQR, Naïve est.)^1^	_Min, Max_	(SE)^3^	_Min, Max_	(SE)	_Min, Max_	(SE)	_Min, Max_	(SE)
*Pseudacris maculata*									
2004	(16, 5, 6, 0.625)	0.675	0.124					0.482	0.056
2005	(17, 10, 4, 0.764)	0.782	0.106	0.462	0.229	0.064	0.088	0.387	0.056
*Lithobates pipiens*									
2004	(9, 10, 4, 0.667)	0.730, 0.734	0.176, 0.175					0.415, 0.431	0.077, 0.047
2005	(9, 12, 4, 0.667)	0.674, 0.675	0.159, 0.159	0.173, 0.183	0.382,0.380	0.143, 0.145	0.145, 0.145	0.431, 0.437	0.047, 0.077

Ranges for each parameter represent the high and low median values across all top models (≤ 5 ΔAIC [Akaike's Information Criterion]) for each species. Single values are due to outcomes with only one top model. Standard errors listed are those associated with the listed minimum and maximum values or the individual estimates from single models.

1 *n*  =  number of sites sampled; 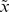  =  median number of times sites were sampled; IQR  =  interquartile range for the median number of times sites were sampled; Naïve est.  =  naïve estimate, which we calculated independently for each year simply as the number of sites surveyed where we detected the species at least once/the total number of potential sites for that species that we surveyed. This value estimates site occupancy without correcting for the detection probability. Note that we used the multiyear option in PRESENCE [47] to estimate ψ for each year, resulting in derived annual estimates after the first year based upon the sum of occupied sites that did not go extinct from the previous year plus unoccupied sites that became colonized, thus making annual comparisons of naïve estimates with ψ less straightforward.

2 Estimate of the occupancy probability.

3 Standard errors for the listed minimum and maximum values or the individual estimates from single models.

4 Estimate of the colonization probability.

5 Estimate of the extinction probability.

6 Estimate of the detection probability.

**Table 4:** Estimated values for parameters from site occupancy models of data collected via daytime surveys in the Upper Mississippi River National Wildlife and Fish Refuge from 2002 to 2005.

*Species*		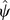 2	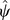	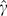 4	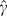	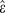 5	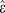	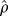 6	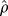
Year	( 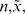 IQR, Naïve est.)^1^	_Min, Max_	(SE)^3^	_Min, Max_	(SE)	_Min, Max_	(SE)	_Min, Max_	(SE)
*Anaxyrus americanus*									
2002	(56, 7, 3, 0.071)	0.129, 0.135	0.091, 0.069					0.119, 0.134	0.054, 0.036
2003	(54, 8, 6, 0.056)	0.102, 0.103	0.035, 0.036	0.092, 0.093	0.036, 0.036	0.825, 0.839	0.159, 0.149	0.111, 0.134	0.036, 0.036
2004	(54, 6, 6, 0.093)	0.100, 0.103	0.036, 0.036	0.092, 0.093	0.036, 0.036	0.825, 0.839	0.159, 0.149	0.134, 0.148	0.036, 0.059
2005	(20, 14, 2, 0.050)	0.100, 0.103	0.036, 0.036	0.092, 0.093	0.036, 0.036	0.825, 0.839	0.159, 0.149	0.119, 0.134	0.054, 0.036
*Hyla chrysoscelis/versicolor* 7									
2002	(56, 7, 3, 0.250)	0.605, 0.694	0.225, 0.276					0.070, 0.076	0.023, 0.023
2003	(54, 8, 6, 0.074)	0.257, 0.268	0.076, 0.077	0.260, 0.313	0.122, 0.149	0.726, 0.768	0.147, 0.110	0.070, 0.076	0.023, 0.023
2004	(52, 6, 6, 0.135)	0.264, 0.292	0.078, 0.105	0.260, 0.313	0.122, 0.149	0.726, 0.768	0.147, 0.110	0.070, 0.076	0.023, 0.023
2005	(20, 14, 2, 0.400)	0.264, 0.289	0.079, 0.094	0.260, 0.313	0.122, 0.149	0.726, 0.768	0.147, 0.110	0.070, 0.076	0.023, 0.023
*Lithobates clamitans*									
2002	(50, 7, 3, 0.660)	0.748	0.075					0.330	0.016
2003	(50, 10, 8, 0.740)	0.612	0.052	0.382	0.100	0.310	0.061	0.330	0.016
2004	(47, 8, 8, 0.298)	0.571	0.063	0.382	0.100	0.310	0.061	0.330	0.016
2005	(19, 14, 1, 0.684)	0.558	0.069	0.382	0.100	0.310	0.061	0.330	0.016
*L. pipiens*									
2002	(52,6, 3, 0.635)	0.760, 0.915	0.077, 0.161					0.380, 0.380	0.023, 0.023
2003	(52, 10, 8, 0.788)	0.707, 0.768	0.050, 0.073	0.376, 0.393	0.119, 0.117	0.189, 0.199	0.048, 0.049	0.380, 0.380	0.023, 0.023
2004	(49, 10, 6, 0.551)	0.684, 0.707	0.061, 0.061	0.376, 0.393	0.119, 0.117	0.189, 0.199	0.048, 0.049	0.380, 0.380	0.023, 0.023
2005	(19, 10, 1, 0.684)	0.675, 0.681	0.071, 0.068	0.376, 0.393	0.119, 0.117	0.189, 0.199	0.048, 0.049	0.307, 0.310	0.028, 0.029

Ranges for each parameter represent the high and low median values across all top models (≤ 5 ΔAIC [Akaike's Information Criterion]) for each species. Single values are due to outcomes with only one top model. Standard errors listed are those associated with the listed minimum and maximum values or the individual estimates from single models.

1 *n*  =  number of sites sampled; 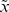  =  median number of times sites were sampled; IQR  =  interquartile range for the median number of times sites were sampled; Naïve est.  =  naïve estimate, which we calculated independently for each year simply as the number of sites surveyed where we detected the species at least once/the total number of potential sites for that species that we surveyed. This value estimates site occupancy without correcting for the detection probability. Note that we used the multiyear option in PRESENCE [47] to estimate ψ for each year, resulting in derived annual estimates after the first year based upon the sum of occupied sites that did not go extinct from the previous year plus unoccupied sites that became colonized, thus making annual comparisons of naïve estimates with ψ less straightforward.

2 Estimate of the occupancy probability

3 Standard errors for the listed minimum and maximum values or the individual estimates from single models.

4 Estimate of the colonization probability.

5 Estimate of the extinction probability.

6 Estimate of the detection probability.

7 Distinguishing between *Hyla chrysoscelis* and *H. versicolor* was not possible based upon external morphology. For these analyses, we used the complex as the taxonomic category.

**Table 5:** Estimated values for parameters from site occupancy models of data collected via daytime surveys in the St. Croix National Scenic Riverway from 2002 to 2005.

*Species*		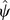 2	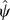	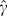 4	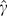	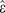 5	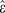	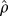 6	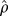
Year	( 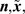 IQR, Naïve est.)^1^	_Min, Max_	(SE)^3^	_Min, Max_	(SE)	_Min, Max_	(SE)	_Min, Max_	(SE)
*Anaxyrus americanus*									
2002	(55, 7, 3, 0.255)	0.508	0.161					0.125	0.026
2003	(60, 14, 6, 0.117)	0.292	0.054	0.299	0.071	0.715	0.101	0.142	0.027
2004	(59, 8, 6, 0.322)	0.295	0.050	0.299	0.071	0.715	0.101	0.130	0.026
2005	(19, 14, 2, 0.263)	0.295	0.050	0.299	0.071	0.715	0.101	0.125	0.026
*Pseudacris crucifer*									
2002	(59, 6, 3, 0.305)	0.484	0.121					0.155	0.031
2003	(62, 12, 6, 0.145)	0.218	0.051	0.078	0.039	0.633	0.091	0.176	0.034
2004	(56, 9, 4, 0.143)	0.141	0.042	0.078	0.039	0.633	0.091	0.174	0.031
2005	(19, 12, 2, 0.158)	0.119	0.043	0.078	0.039	0.633	0.091	0.155	0.031
*P. maculata*									
2002	(59, 6, 3, 0.237)	0.403	0.092					0.188	0.019
2003	(62, 12, 6, 0.323)	0.353	0.068	0.022	0.053	0.155	0.081	0.188	0.019
2004	(56, 10, 6, 0.304)	0.313	0.064	0.022	0.053	0.155	0.081	0.188	0.019
2005	(19, 12, 2, 0.000)	0.280	0.073	0.022	0.053	0.155	0.081	0.188	0.019
*Lithobates clamitans*									
2002	(35, 7, 0, 0.857)	0.993	0.050					0.431	0.027
2003	(36, 14, 2, 0.778)	0.816	0.049	0.689	0.190	0.183	0.049	0.451	0.024
2004	(34, 13, 6, 0.735)	0.793	0.055	0.689	0.190	0.183	0.049	0.431	0.027
2005	(13, 14, 2, 0.846)	0.791	0.059	0.689	0.190	0.183	0.049	0.431	0.027
*L. pipiens*									
2002	(37, 6, 0, 0.324)	0.445, 0.456	0.116, 0.153					0.245, 0.245	0.030, 0.031
2003	(40, 10, 4, 0.450)	0.434, 0.437	0.077, 0.094	0.227, 0.230	0.084, 0.081	0.307, 0.308	0.089, 0.088	0.272, 0.273	0.031, 0.030
2004	(34, 12, 10, 0.382)	0.430, 0.431	0.080, 0.085	0.227, 0.230	0.084, 0.081	0.307, 0.308	0.089, 0.088	0.245, 0.245	0.030, 0.031
2005	(14, 10, 2, 0.429)	0.428, 0.428	0.090, 0.087	0.227, 0.230	0.084, 0.081	0.307, 0.308	0.089, 0.088	0.229, 0.229	0.030, 0.031
*L. septentrionalis*									
2002	(35, 7, 0, 0.086)	0.206	0.097					0.209	0.029
2003	(36, 14, 2, 0.250)	0.214	0.065	0.065	0.050	0.213	0.117	0.209	0.029
2004	(34, 13, 6, 0.088)	0.220	0.067	0.065	0.050	0.213	0.117	0.209	0.029
2005	(13, 14, 2, 0.308)	0.224	0.083	0.065	0.050	0.213	0.117	0.209	0.029
*L. sylvaticus*									
2002	(48, 5, 3, 0.292)	0.307, 0.398	0.125, 0.106					0.342, 0.374	0.032, 0.020
2003	(50, 10, 6, 0.500)	0.530, 0.541	0.063, 0.053	0.446, 0.452	0.069, 0.068	0.278, 0.281	0.067, 0.067	0.374, 0.381	0.020, 0.031
2004	(46, 8, 6, 0.630)	0.593, 0.597	0.060, 0.059	0.446, 0.452	0.069, 0.068	0.278, 0.281	0.067, 0.067	0.374, 0.381	0.020, 0.031
2005	(17, 10, 2, 0.471)	0.610, 0.612	0.063, 0.063	0.446, 0.452	0.069, 0.068	0.278, 0.281	0.067, 0.067	0.342, 0.374	0.032, 0.020

Ranges for each parameter represent the high and low median values across all top models (≤ 5 ΔAIC [Akaike's Information Criterion]) for each species. Single values are due to outcomes with only one top model. Standard errors listed are those associated with the listed minimum and maximum values or the individual estimates from single models.

1 *n*  =  number of sites sampled; 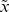  =  median number of times sites were sampled; IQR  =  interquartile range for the median number of times sites were sampled; Naïve est.  =  naïve estimate, which we calculated independently for each year simply as the number of sites surveyed where we detected the species at least once/the total number of potential sites for that species that we surveyed. This value estimates site occupancy without correcting for the detection probability. Note that we used the multiyear option in PRESENCE [47] to estimate ψ for each year, resulting in derived annual estimates after the first year based upon the sum of occupied sites that did not go extinct from the previous year plus unoccupied sites that became colonized, thus making annual comparisons of naïve estimates with ψ less straightforward.

2 Estimate of the occupancy probability.

3 Standard errors for the listed minimum and maximum values or the individual estimates from single models.

4 Estimate of the colonization probability.

5 Estimate of the extinction probability.

6 Estimate of the detection probability.

**Table 6:** Estimated values for parameters from site occupancy models of data collected via daytime surveys in Voyageurs National Park from 2002 to 2005.

*Species*		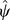 2	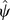	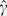 4	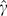	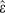 5	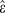	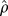 6	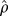
Year	( 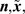 IQR, Naïve est.)^1^	_Min, Max_	(SE)^3^	_Min, Max_	(SE)	_Min, Max_	(SE)	_Min, Max_	(SE)
*Anaxyrus americanus*									
2002	(45, 7, 3, 0.356)	0.425, 0.461	0.105, 0.098					0.151, 0.151	0.032, 0.032
2003	(46, 8, 8, 0.130)	0.204, 0.206	0.049, 0.049	0.107, 0.108	0.037, 0.037	0.676, 0.679	0.104, 0.102	0.151, 0.151	0.032, 0.032
2004	(49, 12, 6, 0.082)	0.151, 0.152	0.042, 0.042	0.107, 0.108	0.037, 0.037	0.676, 0.679	0.104, 0.102	0.151, 0.151	0.032, 0.032
2005	(26, 14, 4, 0.269)	0.139, 0.140	0.041, 0.041	0.107, 0.108	0.037, 0.037	0.676, 0.679	0.104, 0.102	0.255, 0.256	0.044, 0.044
*Hyla chrysoscelis/veriscolor* 7									
2002	(45, 7, 3, 0.178)	0.197, 0.203	0.075, 0.067					0.178, 0.178	0.033, 0.033
2003	(46, 8, 8, 0.130)	0.211, 0.213	0.051, 0.049	0.090, 0.091	0.034, 0.035	0.307, 0.309	0.140, 0.141	0.178, 0.178	0.033, 0.033
2004	(49, 12, 6, 0.163)	0.217, 0.219	0.057, 0.057	0.090, 0.091	0.034, 0.035	0.307, 0.309	0.140, 0.141	0.178, 0.178	0.033, 0.033
2005	(26, 14, 4, 0.269)	0.221, 0.222	0.067, 0.067	0.090, 0.091	0.034, 0.035	0.307, 0.309	0.140, 0.141	0.157, 0.157	0.031, 0.031
*Pseudacris crucifer*									
2002	(47, 6, 3, 0.362)	0.317, 0.414	0.106, 0.084					0.229, 0.229	0.026, 0.026
2003	(50, 10, 6, 0.240)	0.340, 0.382	0.077, 0.053	0.189, 0.191	0.050, 0.050	0.336, 0.347	0.097, 0.097	0.229, 0.229	0.026, 0.026
2004	(52, 10, 6, 0.250)	0.350, 0.367	0.064, 0.062	0.189, 0.191	0.050, 0.050	0.336, 0.347	0.097, 0.097	0.229, 0.229	0.026, 0.026
2005	(26, 16, 4, 0.577)	0.356, 0.361	0.068, 0.069	0.189, 0.191	0.050, 0.050	0.336, 0.347	0.097, 0.097	0.229, 0.229	0.026, 0.026
*Lithobates clamitans*									
2002	(18, 7, 1, 0.611)	0.673	0.130					0.355	0.031
2003	(22, 14, 8, 0.727)	0.676	0.078	0.345	0.122	0.164	0.065	0.355	0.031
2004	(20, 14, 3, 0.550)	0.677	0.085	0.345	0.122	0.164	0.065	0.346	0.033
2005	(13, 16, 0, 0.769)	0.678	0.097	0.345	0.122	0.164	0.065	0.346	0.033
*L. pipiens*									
2002	(20, 6, 2, 0.450)	0.527, 0.539	0.127, 0.142					0.317, 0.318	0.035, 0.035
2003	(24, 10, 7, 0.625)	0.547, 0.550	0.072, 0.076	0.373, 0.378	0.099, 0.099	0.298, 0.298	0.086, 0.087	0.317, 0.318	0.033, 0.033
2004	(22, 12, 4, 0.364)	0.553, 0.556	0.083, 0.083	0.373, 0.378	0.099, 0.099	0.298, 0.298	0.086, 0.087	0.317, 0.318	0.036, 0.036
2005	(14, 14, 0, 0.643)	0.555, 0.559	0.089, 0.089	0.373, 0.378	0.099, 0.099	0.298, 0.298	0.086, 0.087	0.299, 0.299	0.036, 0.036
*L. septentrionalis*									
2002	(18, 7, 1, 0.611)	0.575	0.124					0.324	0.033
2003	(22, 14, 8, 0.545)	0.561	0.103	0.073	0.069	0.079	0.053	0.324	0.033
2004	(20, 14, 3, 0.550)	0.548	0.101	0.073	0.069	0.079	0.053	0.324	0.034
2005	(13, 16, 0, 0.538)	0.537	0.113	0.073	0.069	0.079	0.053	0.324	0.034
*L. sylvaticus*									
2002	(46, 5, 2, 0.717)	0.759	0.072					0.464	0.016
2003	(50, 8, 6, 0.680)	0.770	0.042	0.544	0.108	0.158	0.045	0.464	0.016
2004	(49, 8, 4, 0.776)	0.774	0.049	0.544	0.108	0.158	0.045	0.464	0.016
2005	(26, 14, 4, 0.731)	0.775	0.052	0.544	0.108	0.158	0.045	0.464	0.016

Ranges for each parameter represent the high and low median values across all top models (≤ 5 ΔAIC [Akaike's Information Criterion]) for each species. Single values are due to outcomes with only one top model. Standard errors listed are those associated with the listed minimum and maximum values or the individual estimates from single models.

1 *n*  =  number of sites sampled; 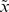  =  median number of times we sampled sites; IQR  =  interquartile range for the median number of times we sampled sites; Naïve est.  =  naïve estimate, which we calculated independently for each year simply as the number of sites surveyed where we detected the species at least once/the total number of potential sites for that species that we surveyed. This value estimates site occupancy without correcting for the detection probability. Note that we used the multiyear option in PRESENCE [47] to estimate ψ for each year, resulting in derived annual estimates after the first year based upon the sum of occupied sites that did not go extinct from the previous year plus unoccupied sites that became colonized, thus making annual comparisons of naïve estimates with ψ less straightforward.

2 Estimate of the occupancy probability.

3 Standard errors for the listed minimum and maximum values or the individual estimates from single models.

4 Estimate of the colonization probability.

5 Estimate of the extinction probability.

6 Estimate of the detection probability.

7 Distinguishing between *Hyla chrysoscelis* and *H. versicolor* was not possible based upon external morphology. For these analyses, we used the complex as the taxonomic category.

### 5. Gross External Deformities

*Ambystoma laterale, A. americanus, Acris crepitans, H. chrysoscelis/versicolor, P. crucifer, P. maculata, L. catesbeiana, L. clamitans, L. pipiens, L. septentrionalis, and L. sylvaticus* were represented (0.05, 4.4, 0.4, 1.2, 2.3, 0.1, 1.0, 24, 39, 13, and 15% of the total number of metamorphs, respectively) among the 5,501 metamorphs we caught and examined for deformities across sites and years. We observed relatively few deformities among these animals. Annual median proportions of metamorphs we caught that were deformed, among sites where we caught at least 20 metamorphs (85 site/year combinations; minimum, maximum, and median  = 20, 169, and 34 metamorphs caught, respectively), were zero for nine of the 13 combinations of conservation area and year sampled (Fig. S6). The largest annual median proportion was 0.012 and the largest proportion of deformed metamorphs at any one site was 0.15 (Fig. S6). Results from a Spearman Rank Test for association between the proportion of metamorphs we caught that were deformed and triazine concentrations for sites (at least 20 metamorphs caught) in 2004 and 2005 suggested no significant association (n = 31, coefficient = −0.0063, p-value = 0.97). We observed no gross external deformities in L. pipiens metamorphs from 11 of the 14 sites where we collected such metamorphs for trematode analyses. Frequencies were<4% at the other three sites and did not appear related to triazine concentrations we measured (Fig. S7).

### 6. Trematode Infections

**Figure 3:**
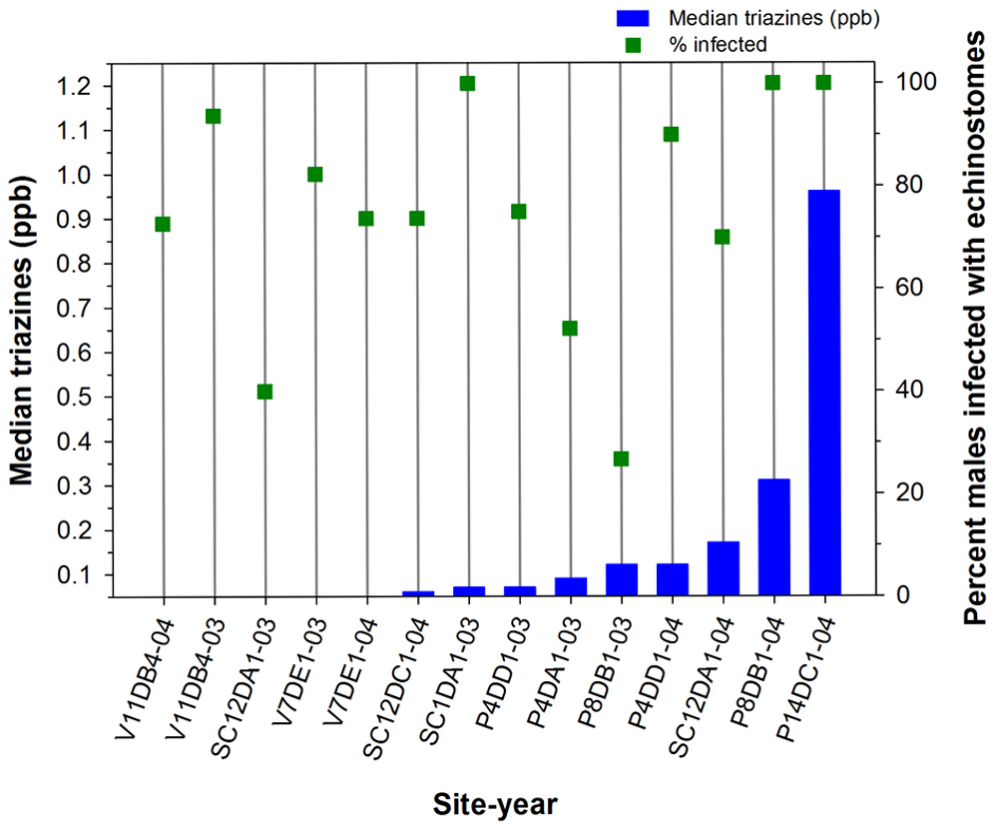
Median triazine concentrations and the percent of male *Lithobates pipiens* metamorphs with echinostome infections at individual breeding sites. Sites identified with a V, SC, or P were in Voyageurs National Park, the St. Croix National Scenic Riverway, and the Upper Mississippi River National Wildlife and Fish Refuge, respectively. Triazine concentrations at sites with no vertical bars were below the detection limit (0.050 ppb) of the enzyme-linked immunosorbent assay used to analyze water samples.

**Figure 4:**
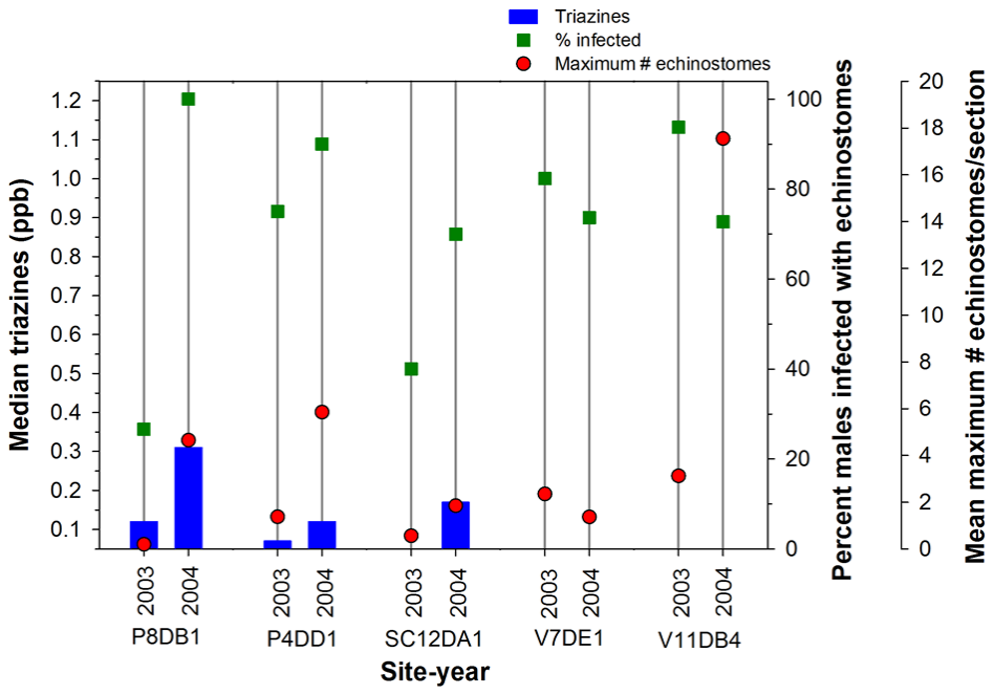
Median triazine concentrations, the percent of male *Lithobates pipiens* metamorphs with echinostome infections, and the mean maximum number of echinostomes measured per metamorph kidney section. Sites identified with a V, SC, or P were in Voyageurs National Park, the St. Croix National Scenic Riverway, and the Upper Mississippi River National Wildlife and Fish Refuge, respectively. Triazine concentrations at sites with no vertical bars were below the detection limit (0.050 ppb) of the enzyme-linked immunosorbent assay used to analyze water samples.

The percentage of individual metamorphs infected with echinostomes (echinostome infection rate) was high across most sites, including sites with triazine concentrations below the ELISA detection limit (<0.05 ppb). The echinostome infection rate did not appear related to triazine concentrations overall (Fig. 3), as suggested further by the results from the Spearman Rank Test (n = 14, coefficient = 0.23, p-value = 0.44). However, echinostome infection rates and the maximum number of echinostome infections per individual (severity of infections) were higher when triazine concentrations were higher for the three sites (P8DB1, P4DD1, and SC12DA1) we sampled in both years that had triazine concentrations above the detection limit (Fig. 4). Forty percent of the animals from SC12DA1 were infected with echinostomes when we did not detect triazines (2003) and 70% were infected when the median triazine concentration was 0.17 ppb (2004) and the severity of infections was>3× higher in 2004 compared with 2003 (Fig. 4).

Similarly, echinostome infection rates at site P4DD1were 75% and 90% when median triazine concentrations were 0.070 ppb (2003) and 0.12 ppb (2004), respectively, and the severity of infections was >4× higher when triazine concentrations were higher in 2004 (Fig. 4). At site P8DB1, one of only two of these sites where triazine levels were above 0.2 ppb, echinostome infection rates were 27% and 100% when median triazine concentrations were 0.12 ppb (2003) and 0.31 ppb (2004), respectively, and the severity of infections was 23× greater when the median concentration was 0.31 ppb (Fig. 4). Thus, echinostome infection rates and the severity of infections were higher when triazine concentrations were higher at these three sites. In addition, site P14DC1 was the southernmost site where we collected metamorphs in the floodplain of the Mississippi River and had the highest concentration of triazines we measured among this subset of sites (Fig. 3). All metamorphs collected from P14DC1 were infected with echinostomes (Fig. 3) and the severity of infections was 1.2× greater than in animals from P8DB1 (data not shown). We collected *L. pipiens* metamorphs from P14DC1 only in 2004, but this result further suggested possible associations between triazine concentrations, echinostome infection rates, and the severity of infections.

Although triazine concentrations were below detection limits at V7DE1 and V11DB4 in 2003 and 2004, infection rates and the severity of infections in metamorphs from these sites appeared to reflect each other across 2003 and 2004 (Fig. 4), except for V11DB4 in 2004, when the severity of infections was considerably greater than for metamorphs from this site in 2003 or from any other site in either year. We have no information that suggests why the severity of infections in metamorphs from this site was so remarkably high in 2004.

### 7. Atrazine and Other Herbicides

#### 7.1. Results via ELISA

**Figure 5:**
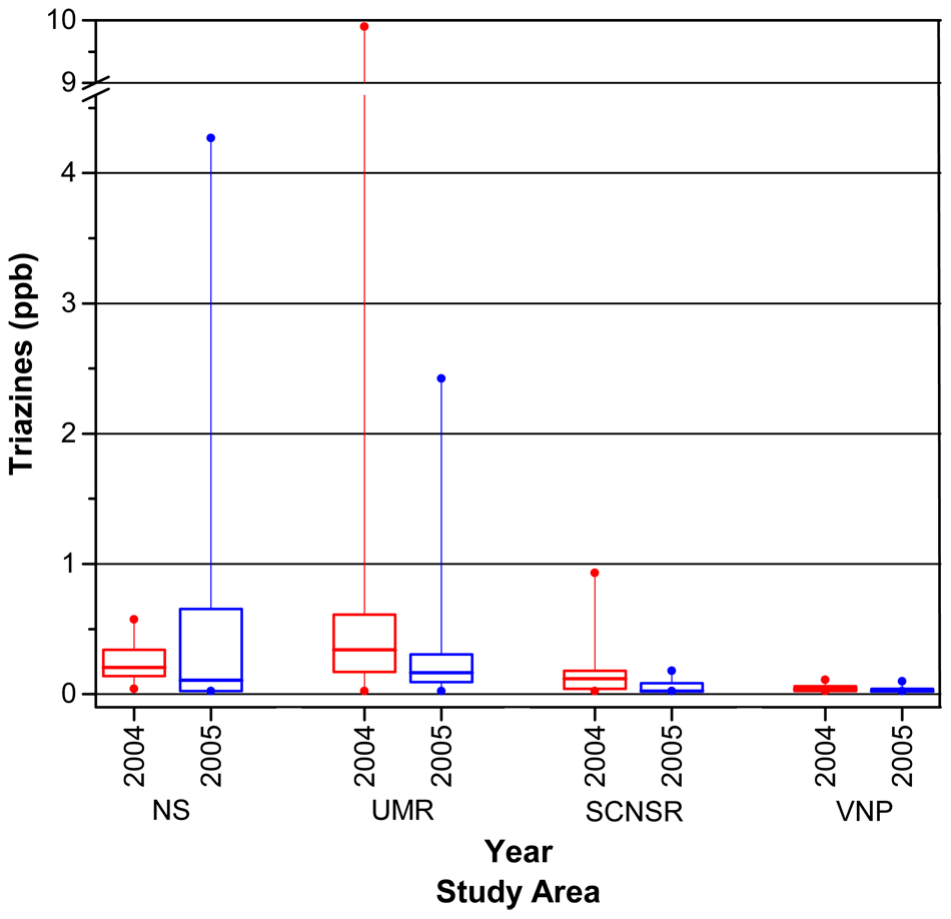
Triazine concentrations measured in water samples from amphibian breeding sites across study areas via an enzyme-linked immunosorbent assay. NS  =  the Neal Smith National Wildlife Refuge; UMR  =  the Upper Mississippi River National Wildlife and Fish Refuge; SCNSR  =  the St. Croix National Scenic Riverway; VNP  =  Voyageurs National Park. Values do not include samples from the main channel of the Mississippi River in the UMR. Horizontal lines within boxes indicate median values. The middle 50% of the data are contained within each box. Vertical lines attached to tops and bottoms of boxes extend to the highest and lowest values. Reference line at 0.05 ppb  =  the detection limit of the assay.

We sampled 14, 152, and 89 individual breeding sites for triazines across all study areas during 2003, 2004, and 2005, respectively, and analyzed them via the ELISA. Based upon our results, triazine concentrations varied considerably among individual sites within study areas during 2004 and 2005, but breeding sites in the UMR and NS as a whole had higher concentrations than those in the SCNSR, which in turn had higher concentrations than those in VNP (Fig. 5). These relative concentrations followed the physical proximity of each study area to extensive areas of corn production (Figs. 1, S1), where atrazine presumably was applied in large quantities (Fig. S3). Of the 523 (45 – NS, 270 – UMR, 94 – SCNSR, 114 – VNP) water samples represented in our 2004 to 2005 data set, including multiple samples from the same sites, 161 (30.8%) mean triazine concentrations (of two laboratory replicates of one water sample from the field) were below the ELISA detection limit of 0.05 ppb, mostly for sites in the SCNSR and VNP. Concentrations in the UMR, SCNSR, and VNP tended to be lower in 2005 (Fig. 5).

**Figure 6:**
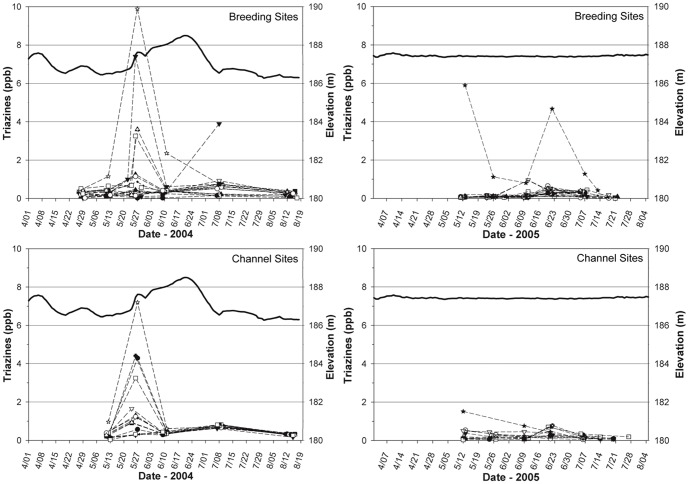
Triazine concentrations measured in water samples collected from amphibian breeding sites and sites in the main channel of the Mississippi River in the Upper Mississippi River National Wildlife and Fish Refuge via an enzyme-linked immunosorbent assay. Symbols represent individual sites sampled. Symbols used for individual sites are consistent across graphs. Solid line near the top of each graph is the hydrograph of the Mississippi River (http://www2.mvr.usace.army.mil/WaterControl/new/layout.cfm) averaged across pools in which we sampled.

We detected triazines at most floodplain breeding sites and in the main channel of the Mississippi River in the UMR in 2004 and 2005. Concentrations from channel sites generally were similar to those from the slightly more elevated amphibian breeding sites sampled across all pools in either year (Fig. S8). Concentrations in the UMR increased at different times in May, June, and July of 2004, most obviously in relation to spikes in water levels in the main channel, but tended to be lower and less variable in relation to more consistent water levels in 2005 (Fig. 6).

Linear regressions (TIBCO Spotfire S+ 8.1 for Windows; Boston, MA, USA) on the ELISA results from filtered and unfiltered samples showed little difference between the two types (Figs. S9a, b), suggesting our results from analyzing unfiltered samples via the ELISA would not have differed if we had filtered samples beforehand and that atrazine and any other cross-reacting triazines present in samples did not bind to particles.

#### 7.2. Results via LCMS analyses

Based on the results from the LCMS analyses, atrazine and atrazine degradation products were present almost exclusively in samples from UMR sites, where we focused most of our site sampling for the ELISA-LCMS comparisons (Tables S13–14). We detected atrazine in all water samples collected at all UMR sites, with concentrations as high as 4.36 µg/L. Atrazine and its degradation products, including deethlyatrazine, hydroxyatrazine, and deisopropylatrazine, were present at most of the UMR sites and concentrations varied with site and across years (Tables S13–14). We detected atrazine (0.04 µg/L) in a sample from one site in the SCNSR, but did not detect atrazine in any samples from VNP (Tables S13–14).

Linear regression suggested the LCMS results for atrazine were related to the ELISA results over the entire range of concentrations measured (Fig. S9b). However, this regression was affected strongly by the two water samples with the highest concentrations (Fig. S9b), which were noticeably higher than the concentrations for other samples. When we removed the two highest LCMS concentrations from the data set, the resultant regression showed a better fit to the data (Fig. S9c) and a similar trend for both analytical methods. This regression also indicated the ELISA concentrations were 36% higher than those obtained for the atrazine parent compound via LCMS (Fig. S9c). This could have been due to potential cross-reactions of the ELISA antibodies with triazines other than atrazine that were present in the samples, some of which we tested for and detected in various concentrations via the LCMS analyses (Tables S13–14), and some for which we did not analyze via the LCMS analyses.

## Discussion

Agricultural land use from the mid-1800s to the present has caused extensive loss and fragmentation of amphibian and other wildlife habitat in the midwestern United States (e.g., [7]) and greatly altered water retention, movement, and quality across the landscape [4]. Agricultural land use continues to be extensive and in flux in this region as farmers expand production [22], likely causing additional impacts to amphibians and their habitats [4]–[6]. Resource managers, conservationists, and other stakeholders largely lack integrated field information describing the recent statuses of midwestern amphibian populations and potential threats they face from agricultural production.

Our assessment was limited to four study areas in which landscapes were protected and managed for conservation. In addition, our results pertain only to these four areas because we did not select them randomly from all possible conservation areas in this region. We also studied these areas for limited periods: two years for the NS and four years for the UMR, SCNSR, and VNP. These relatively short time periods, coupled with a lack of historical survey data for wetlands within these areas and our inability to extrapolate our results beyond our study areas, limit our ability to interpret our observations within a broader temporal and spatial context. Despite these limitations, our results provide useful information regarding the recent statuses of amphibian populations and the relative threat of exposure to atrazine in areas and wetlands that varied in their proximity to intensive corn production.

### 1. Amphibian Species Diversity, Presence, and Site Occupancy

We detected all the amphibian species with historical ranges that overlapped each of our study areas and that we likely could detect via the sampling methods we used (Tables 1, 2), except for *A. texanum* in the NS, and accounted for a large majority of all the species possible for each area. Thus, amphibian species diversity at the conservation-area scale did not appear related to the extent of any nearby habitat alterations or the potential for exposure to triazines (Fig. 5; Tables S13–14) from corn production (Figs. 1, 2). Similarly, Knutson et al. [54] sampled a set of wetlands in unprotected agricultural areas in southeastern Minnesota and reported no differences in amphibian species richness in relation to the proximity of cropland, including corn, compared to pasture or native land cover.

Our results also provide information regarding site occupancy for individual species within and across our study areas. Two advantages of modeling occupancy are 1) deriving a more theoretically unbiased estimate of the true occupancy probability based upon repeated sampling and estimating detection probabilities [55]–[57] and 2) being able to use variables describing site or sampling conditions as covariates in the models to investigate their influence on parameter estimates (e.g., [55], [58]–[61]). This approach is an improvement over relying solely on naïve estimates of presence/absence and the assumption that detection is perfect [60].

Our site-occupancy estimates pertaining to *L. pipiens* were particularly interesting because this species reportedly has declined in distribution and abundance, appears vulnerable to various direct and indirect effects of atrazine exposure [15], and ranges widely across the landscape and different habitat types [62]. It also was the only species for which we modeled site occupancy that occurred in all four of our study areas. Our ψ estimates for 2004 and 2005 suggest *L. pipiens* occupied roughly 43–92% of wetlands across study areas according to the order NS > UMR > VNP > SCNSR (Tables 3, 4, 5, 6). However, our estimates of ρ followed the same order, indicating we generally were less successful detecting *L. pipiens* in the SCNSR and VNP (Tables 3, 4, 5, 6). These similar trends in estimates of ψ and ρ preclude discussing differences in occupancy for *L. pipiens* among study areas, given that more or improved sampling effort in the SCNSR and VNP might have reduced or eliminated such apparent differences (see Text S5 for further discussion of this issue). Regardless of any differences in estimated site occupancy for *L. pipiens* among study areas, the NS and UMR were closest to intensive corn production (Figs. 1, 2) and were small, and long and narrow, respectively, thereby providing limited spatial buffers relative to any effects of such production on habitat quantity and quality. In fact, the NS not only was small in size, but was practically surrounded by farms producing corn and/or soybeans. These factors increased the likelihood that NS populations of *L. pipiens* had been exposed to potential effects from corn production, as the triazine concentrations we measured in samples from breeding sites suggested (Fig. 5; Tables S13–14). Thus, we would have expected substantially lower estimates of ψ than roughly 67–92% for the NS and the UMR if nearby corn production dramatically had reduced fitness recently among *L. pipiens* in those areas. The only indication we have that proximity to corn production might have been related to the occupancy of *L. pipiens* in any study area is that the covariate, % crops, was moderately important for improving estimates of ψ for *L. pipiens* in the UMR (- coefficient; Table S10). This improvement was slight at best based upon comparing the ψ estimates (0.684 [2004] and 0.675 [2005]) from the highest ranked model for *L. pipiens* in the UMR with no covariates on ψ (Table S6) with the ranges of annual estimates of ψ from all the top models for *L. pipiens* in the UMR, including those with covariates on ψ (0.684–0.707 [2004] and 0.675–0.681 [2005]; Table 4).

Our estimates of ψ and ρ for *P*. *maculata* in the NS were similar to such estimates for *L. pipiens* in this refuge (Table 3). For the UMR, our estimates of ψ and ρ for *L. clamitans* were relatively high compared to such estimates for *A. americanus* and *H. chrysoscelis/versicolor* (Table 4). % crops was not an important covariate on ψ for any of these other species in either refuge (Tables S9–S10). Thus, in addition to *L. pipiens*, the other species we detected with the most confidence in the NS and the UMR also appeared to occupy substantial proportions of wetlands regardless of the proximity to corn production.

For a coarse comparison, results from statewide call surveys for amphibians conducted annually since the 1980s or 1990s in Iowa (NS), Minnesota (UMR, SCNSR, VNP), and Wisconsin (UMR, SCNSR) have shown relatively consistent presence for most of the same species we studied [26]–[27], [29]–[30], although Minnesota reported fewer detections for *H. versicolor* and *H. chrysoscelis* [29] and Wisconsin reported possible downward trends in the overall abundance of *L. pipiens* and *L. palustris* [30]. State reports of these broad-scale survey results do not consider any potential relations to environmental factors. However, Knutson et al. [25] reported they used data from the statewide call surveys in Iowa and Wisconsin to assess relations between the presence of amphibian species at wetlands and the proximity of agricultural land, but did not observe any. In contrast, Bonin et al. [24] reported negative associations between amphibian species diversity and agricultural land use in Quebec, which included areas of intensive corn production, based upon results from more limited call surveys.

### 2. Gross Physical Deformities and Trematode Infections

Trematode-induced deformities in amphibians can indicate degraded water quality from inputs of fertilizers and herbicides and potentially result in population declines via reduced individual fitness (e.g., [63]–[65]. Johnson et al. [64] described how eutrophication increased the abundance of algal food for snails, the intermediate hosts parasitic trematodes require, which led to increased trematode abundance and infection rates in amphibians. Kiesecker [66], Rohr and McCoy [15], and Rohr et al. [65] reported that atrazine also was associated with increased rates of trematode infections, including at field sites. Rohr et al. [65] described how atrazine can reduce the abundance of algae in the water column to the benefit of the periphyton on which host snails feed, resulting in increased abundance of snails, trematodes, and amphibian infection rates. They sampled 18 wetlands in Minnesota and reported atrazine concentrations predicted 51% of the variation in abundance of parasitic larval trematodes in recently metamorphosed *L. pipiens* and the combined concentrations of atrazine and the agricultural fertilizer, phosphate, predicted 74%. Atrazine concentrations at Rohr et al.'s [65] sites (mean: 0.179 µg/L; se: ±0.034 µg/L; maximum: 0.59 µg/L) appeared comparable to concentrations we measured at multiple sites in the NS and UMR (Fig. 5) after adjusting our atrazine estimates downward based upon calibrating our ELISA results with the LCMS results. Results from our exploratory assessment of phosphorous concentrations in our study areas (Text S6) also suggested a range of concentrations similar to those Rohr et al. [65] reported. Phosphorous concentrations hypothetically would have been higher in our UMR and NS study wetlands due to their closer proximity to agricultural production (e.g., [4]).

Trematode-infection rates we measured in recently metamorphosed *L. pipiens* ranged from 27 to 100% (median = 74%), but did not appear related to triazine concentrations we measured in water samples from the wetlands where we collected these metamorphs (Fig. 3). Water samples from five of these wetlands did not have detectable concentrations of triazines (Fig. 3). Intriguing, however, were our observations that infection rates were higher in years when triazine concentrations were higher (Fig. 4) for those sites where we detected triazines, possibly suggesting infection rates were associated with atrazine concentrations at those sites similar to observations Rohr et al. [65] reported. These results show that analyses of more metamorphs and water samples from more wetlands and years are necessary to address this issue with confidence for our study areas.

We did not observe any apparent relation between the high trematode-infection rates we observed and rates of gross physical deformities among the *L. pipiens* metamorphs we collected for trematode analyses (Figs. 3a, S7), which is interesting given known relationships between these two phenomena (e.g., [67]). In addition to the *L. pipiens* we collected for trematode analyses and for which we assessed deformities separately, *L. pipiens* accounted for 39% of the metamorphs we surveyed for deformities across all four study areas and were a substantial proportion of those we sampled in the UMR, where we estimated atrazine concentrations generally were similar to those reported by Rohr et al. [65]. However, we observed low frequencies of gross physical deformities, as one potential ramification of trematode infections, in *L. pipiens* or other species regardless of the proximity of our study wetlands to corn production or their atrazine concentrations. Nonetheless, the substantial trematode infection rates we measured in animals from a subset of our study wetlands raise important questions regarding the drivers of these infections and their effects on the fitness of amphibians that bred at our study wetlands given we did not measure trematode-related mortality among larvae or the long-term survival and ultimate reproductive success of metamorphs. See Text S7 for discussion regarding rates of deformities reported for amphibians relative to agricultural areas in the United States and Quebec.

### 3. Atrazine and Other Herbicides

Triazine concentrations were higher in wetlands in study areas closer to intensive corn production (Figs. 1, 4). Results from our comparative LCMS analyses (Tables S13–14) suggested atrazine was the dominant triazine compound in these wetlands and in the samples we analyzed via the ELISA. These results corroborate those of others who reported atrazine can be transported downwind, downhill, or downstream of application areas (summarized, e.g., in [15]) to amphibian breeding sites [65], [68]–[70], regardless of whether sites are in protected conservation areas [69]. Transport to amphibian breeding sites in all four of our study areas had to occur via atmospheric transport and dry and wet deposition, especially given the exclusively palustrine nature of wetlands in the NS, SCNSR, and VNP and their elevations relative to any nearby rivers or streams. Our detections of triazines at a small number of sites in VNP, the boundary of which we estimated was at least 128 km from substantial areas of corn production (Fig. 2), also suggested such transport occurred over considerable distances. In addition to likely atmospheric transport and deposition, runoff draining into the Mississippi River floodplain from areas of intensive corn production in the Upper Mississippi Basin was an obvious mechanism of atrazine transport into the UMR and added another dimension to consider regarding the dynamics of fate and transport of, and potential exposure to, agricultural chemicals for amphibians in this refuge.

Similar to results observed by McDaniel et al. [69] and Byer et al. [71], our results from the LCMS analyses indicated that atrazine accounted for less than the full concentration we measured via the ELISA (approximately 36% less), possibly due to the cross reactivity of the antibodies with the other triazines we described earlier. Even at 36% less, the range of atrazine concentrations we estimated via the ELISA were relevant to atrazine concentrations, either as atrazine alone or as a component of complex mixtures, for which several amphibian researchers reported various direct and indirect effects of exposure (summarized in [15]) and were similar to ranges reported for field sites by Bishop et al. [72], McDaniel et al. [69], Murphy et al. [70], and Rohr et al. [65]. Thus, at some risk of reduced accuracy in measuring the atrazine parent compound or of potentially measuring, but not identifying, other triazines present in samples, the ELISA enabled us to assess relative potential amphibian exposure to atrazine at many breeding sites across fairly broad spatial and temporal scales in these four areas and to describe this potential exposure relative to land use and indicators of the statuses of amphibian populations. Due to resource limitations, we could not have done this by relying on more expensive analytical methods, such as LCMS, a conclusion also reached by McDaniel et al. [69]. Their results and ours, however, do emphasize the need for some level of complementary, more exacting analysis, such as LCMS or gas chromatography-mass spectrometry, to calibrate the ELISA results, as also suggested by Byer et al. [71]. Collectively, these results also suggest a need for a better understanding of the relative proportions and toxicity of atrazine, atrazine degradates, other triazines, and non-triazine toxicants present at field sites.

We observed considerable spatial and temporal variation in triazine detections and concentrations (Figs. 4, 5), as reported from previous field studies [65], [69]–[70], [72]. Aside from the spatial variation related to distance from intensive corn production, the temporal variation we observed was notable between 2004 and 2005 (Figs. 4–5) and within the UMR within a season (Fig. 6). The drivers of such variation appear to be related at least to weather coupled most likely with farmer behavior in terms of when they planted corn and applied atrazine. For example, 2004 was substantially wetter and warmer during May (Figs. S10–11), a primary corn-planting period in this region, especially in Minnesota and Wisconsin compared to typically earlier plantings in Iowa. The much greater rainfall in 2004 likely contributed to the higher ranges of triazine concentrations we observed in the UMR, SCNSR, and VNP (Fig. 5) via atmospheric transport and leaching and helped explain the increased water levels in the Mississippi River from mid-May through June of 2004 (Fig. 6), when rain made its way into the floodplain as runoff.

Triazine concentrations increased in our samples from main-channel and amphibian breeding sites in the UMR in association with the early stages of the spike in the hydrograph of the Mississippi River during 2004 and then subsided by the time the hydrograph peaked (Fig. 6). This likely shows how weather affected triazine concentrations and increased potential amphibian exposures at UMR breeding sites via surface runoff, atmospheric transport, and precipitation-induced leaching of the atmosphere. Concentrations in the UMR during this period were among the highest we measured across sites, study areas, and years and estimated atrazine levels were well within ranges reported to cause effects in amphibians [15]. In addition, atrazine concentrations represented in the triazine concentrations we report here likely were a part of complex mixtures of agricultural chemicals and other toxicants present in the UMR [73], as well as the NS and, to a lesser extent, the SCNSR. Synergistic effects of atrazine on amphibians can occur in low concentrations in such mixtures based upon experimental studies [10], [15]. See Text S8 for further discussion of triazine concentrations in the NS and UMR, including between breeding sites and main-channel sites of the Mississippi River.

Overall, our results add to the limited data published previously on atrazine concentrations in amphibian breeding wetlands in the midwestern United States and elsewhere relative to the proximity of corn production. They also provide insights into the likely transport mechanisms for atrazine and other potential toxicants into our study areas and the general relevance of atrazine concentrations measured in river samples to concentrations measured in samples from nearby palustrine breeding sites. Importantly, they also demonstrate considerable variability in the potential for amphibian populations to be exposed to atrazine in the wild.

### 4. Drivers of Variation in Potential Exposure to Atrazine

Weather is a primary driver of variation in the potential for amphibian exposure to atrazine, as well as nitrogen, phosphorous, and other agricultural chemicals used to produce corn. Precipitation and temperature varied substantially across midwestern states within and across key corn-production months from 2003 to 2005 (Figs. S10–11). Precipitation in 2004, particularly in May, was associated strongly with coincidental increases in water levels and triazine concentrations in the UMR compared to less dynamic interactions of this type in 2005 (Figs. 5, S10). Variable combinations of regional temperatures (Fig. S11) and snow cover around the NS from 2003 to 2005 (Fig. S12) indicated how differences in weather likely caused farmers to plant corn and apply atrazine and other chemicals differently across years, as illustrated further in the variation in corn green-up around the NS from 2003 to 2005 (Fig. S13). Another factor contributing to seasonal and annual variation in the potential for amphibians to be exposed to agricultural chemicals is that midwestern farmers typically rotate planting of corn and soybeans among the same fields (Fig. S14), adding to the complexity of chemical use and transport within and across years and to the complexity and uncertainty of exposure dynamics related directly to weather, especially precipitation events, alone. Other farming practices, such as tillage, irrigation, and drainage methods, also likely influence overall transport and exposure dynamics.

Weather also affects amphibian metabolism and behavior in ways that can dictate when and where they breed in a given year, as we observed during this study and repeatedly since in the UMR, SCNSR, and other midwestern locations. In turn, this affects the likelihood that amphibians might be exposed to atrazine during vulnerable developmental windows [15]. Weather-induced variation in amphibian breeding phenology and farmer behavior suggests that any exposures of amphibians to atrazine at our study sites likely varied in magnitude, timing, and duration during and across seasons. Thus, assessing risks of atrazine exposure for wild amphibian populations requires more and improved information describing the likelihood of effects given the interactions of at least these very dynamic factors across the landscape of interest.

Based upon the dynamics we described here, we speculate that effects of exposure to atrazine or other agricultural chemicals during key developmental windows might occur in the NS and UMR in particular in some years, but not other years. Speaking generally, any effects from exposure to, and uptake of, atrazine might be inconsistent enough within and across years to allow for successful reproduction sufficient to maintain requisite age classes in the population. Thus, the boom-or-bust reproductive strategy of these amphibian species could allow populations to persist, similar to how amphibian populations have persisted in the face of variably unfavorable climate conditions that can reduce fitness or eliminate entire cohorts in some years. Of course, such dynamics have to be considered within the context of effects on populations from multiple stressors, including declines due to outright habitat loss or more direct effects of climate change, for example. These considerations reinforce the need to obtain more data from field studies to complement and calibrate results from non-field studies and to establish a richer context for evaluating risks amphibian populations face in agricultural landscapes.

### 5. Conclusions

Our results indicate that amphibian species richness in the four conservation areas we studied, and site occupancy within each area, were not related to the proximity of these areas and wetlands to intensive corn production, although the likelihood that amphibians were exposed to atrazine in wetlands was greater in study areas closer to such production. The proportion of recent metamorphs with gross physical deformities was low in wetlands we sampled across all study areas, yet rates of trematode infections were high in metamorphs of *L. pipiens* we collected from a subset of these sites, even when we did not detect triazines in water samples. Atrazine was transported atmospherically and/or hydrologically into our study areas, including across long distances via atmospheric transport into VNP. Triazine concentrations in water samples we collected from the main channel of the Mississippi River and in nearby slightly perched amphibian breeding wetlands in the UMR overlapped and showed strong relations to annual rainfall patterns and associated changes in the river's hydrograph. Collectively, our results provide resource managers and other stakeholders previously nonexistent baseline information on the statuses of amphibian populations and the potential for resident amphibians to be exposed to atrazine in these conservation areas, as well as on some of the complex dynamics that can drive exposure to atrazine in the field.

## Supporting Information

Figure S1 **Acres planted in corn in the conterminous United States during 2012.**
(DOC)

Figure S2 **The percent of all cropland acreage, excluding pastured cropland, farmers fertilized in the United States during 2007.**
(DOC)

Figure S3 **Estimated application rates for atrazine in the conterminous United States and the estimated percentage applied to corn.**
(DOC)

Figure S4 **Acres of corn planted in the United States from 1993–2013.**
(DOC)

Figure S5 **Map of study areas and associated Level-III Ecoregions.**
(DOC)

Figure S6 **Boxplots of proportions of metamorphs with gross external deformities per site per conservation area and year.**
(DOC)

Figure S7 **Median triazine concentrations and the percent of** ***Lithobates pipiens*** **metamorphs with gross external deformities.**
(DOC)

Figure S8 **Comparisons of triazine concentrations in water samples from amphibian breeding sites and main channel sites in the UMR.**
(DOC)

Figure S9 **Linear regressions on results from ELISA and LCMS analyses of water samples collected from the UMR, SCNSR, and VNP.**
(DOC)

Figure S10 **Total seasonal monthly precipitation across the region containing our study areas from 2003 to 2005.**
(DOC)

Figure S11 **Seasonal monthly average daily temperatures across the region containing our study areas from 2003 to 2005.**
(DOC)

Figure S12 **Percent snow cover for a 385 km^2^ landscape block centered on the NS from 2003 to 2005.**
(DOC)

Figure S13 **The Normalized Difference Vegetation Index for corn in a 385 km^2^ landscape block centered on the NS from 2003 to 2005.**
(DOC)

Figure S14 **Relative distributions of lands planted in corn within a 385-km^2^ landscape block centered on the NS from 2003 to 2005.**
(DOC)

Table S1 **Descriptions of the Level-III Ecoregions in which our study areas were located.**
(DOC)

Table S2 **Summary of wetlands surveyed during daytime amphibian surveys.**
(DOC)

Table S3 **Summary of the number of nighttime call surveys for amphibians.**
(DOC)

Table S4 **Summary of the number of wetlands surveyed for amphibian deformities.**
(DOC)

Table S5 **Output from PRESENCE ranking the top occupancy models for the NS.**
(DOC)

Table S6 **Output from PRESENCE ranking the top occupancy models for the UMR.**
(DOC)

Table S7 **Output from PRESENCE ranking the top occupancy models for the SCNSR.**
(DOC)

Table S8 **Output from PRESENCE ranking the top occupancy models for VNP.**
(DOC)

Table S9 **Summary of results pertaining to covariates from the top occupancy models for the NS.**
(DOC)

Table S10 **Summary of results pertaining to covariates from the top occupancy models for the UMR.**
(DOC)

Table S11 **Summary of results pertaining to covariates from the top occupancy models for the SCNSR.**
(DOC)

Table S12 **Summary of results pertaining to covariates from the top occupancy models for VNP.**
(DOC)

Table S13 **Triazine concentrations in amphibian breeding sites in the SCNSR, UMR, and VNP.**
(DOC)

Table S14 **Additional triazine concentrations in amphibian breeding sites in the SCNSR, UMR, and VNP.**
(DOC)

Text S1 **Studies of amphibians and atrazine in the midwestern United States.**
(DOC)

Text S2 **Details on the process of selecting study wetlands.**
(DOC)

Text S3 **Details regarding how we measured land-cover variables for use as covariates on ψ in occupancy models.**
(DOC)

Text S4 **Occupancy estimates − outcomes specific to study areas.**
(DOC)

Text S5 **Relative difficulty of detecting different genera.**
(DOC)

Text S6 **Results from exploratory sampling for phosphorous.**
(DOC)

Text S7 **Amphibian deformities reported relative to agricultural areas in the United States and Quebec.**
(DOC)

Text S8 **Further discussion of triazine concentrations in the NS and the UMR.**
(DOC)
