# Supplementary material for: Indicators of the Statuses of Amphibian Populations and Their Potential for Exposure to Atrazine in Four Midwestern U.S. Conservation Areas
Source: PLoS One. 2014 Sep 12;9(9):e107018. doi: 10.1371/journal.pone.0107018 (PMC4162561; doi:10.1371/journal.pone.0107018)
Supplement: Figure S1 — Acres planted in corn in the conterminous United States during 2012. (DOC) [file pone.0107018.s001.doc]

**Supporting Information**


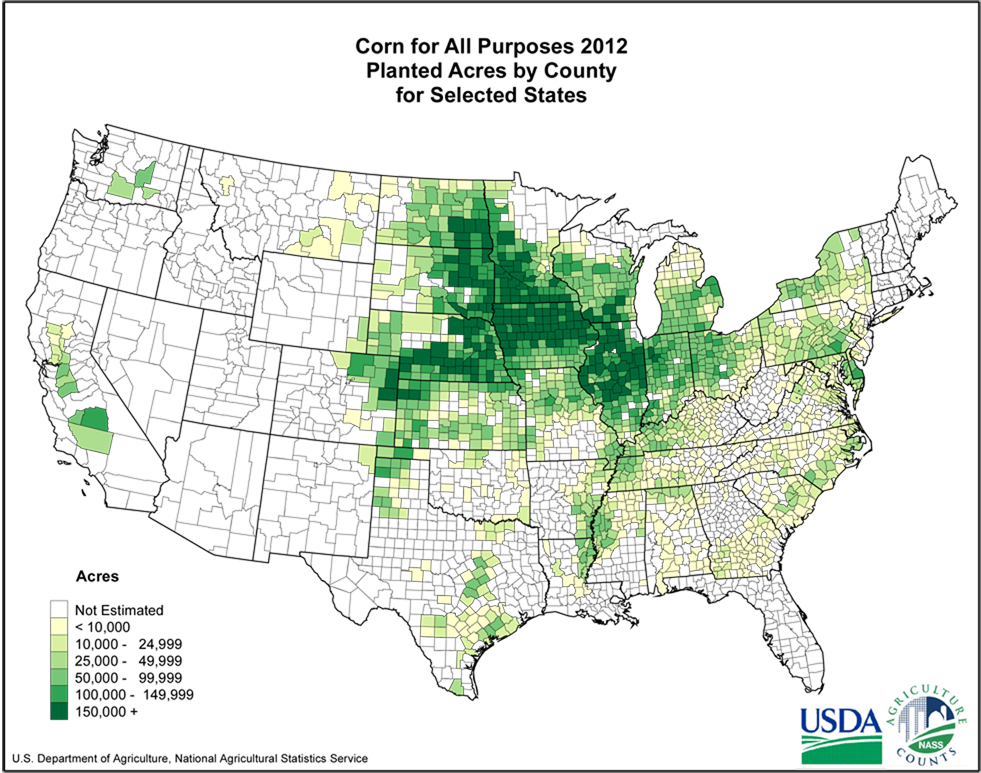


**Figure S1.** **Acres planted in corn in the conterminous United States during 2012.**

Available: http://www.nass.usda.gov/Charts_and_Maps/Crops_County/cr-pl.asp. Accessed 10 April 2014.
