# Supplementary material for: Indicators of the Statuses of Amphibian Populations and Their Potential for Exposure to Atrazine in Four Midwestern U.S. Conservation Areas
Source: PLoS One. 2014 Sep 12;9(9):e107018. doi: 10.1371/journal.pone.0107018 (PMC4162561; doi:10.1371/journal.pone.0107018)
Supplement: Figure S2 — The percent of all cropland acreage, excluding pastured cropland, farmers fertilized in the United States during 2007. (DOC) [file pone.0107018.s002.doc]

**Supporting Information**


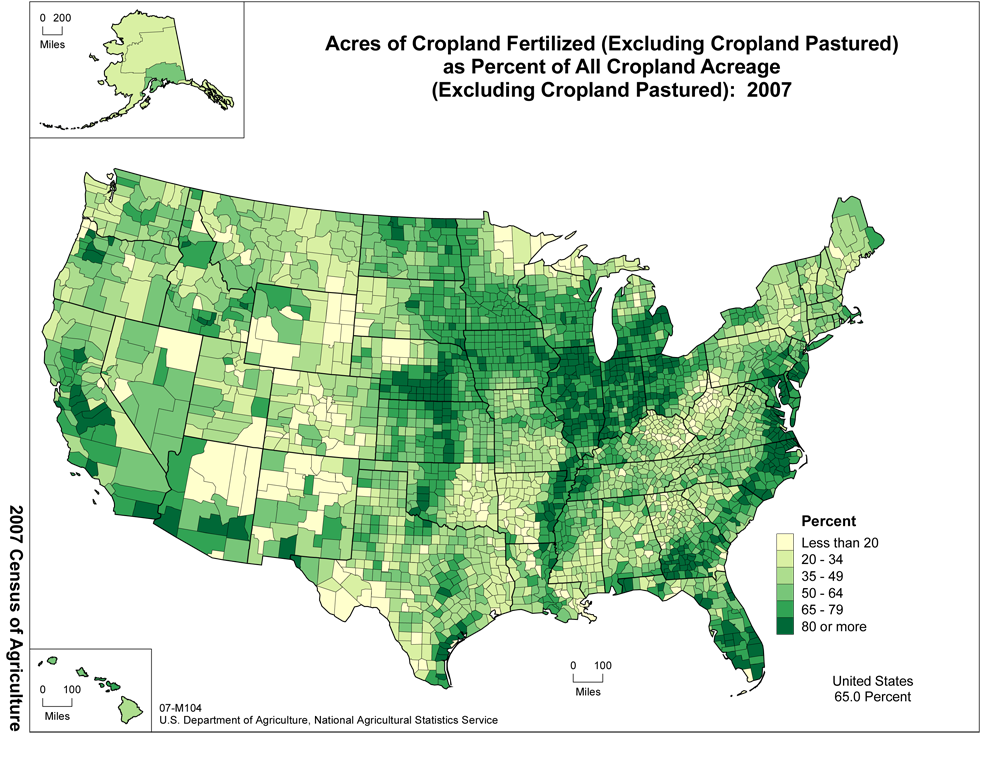


**Figure S2. The percent of all cropland acreage, excluding pastured cropland, farmers fertilized in the United States during 2007.**

Available: http://www.agcensus.usda.gov/Publications/2007/Online_Highlights/Ag_Atlas_Maps/Farms/Agricultural_Chemicals_Used/07-M104-RGBChor-largetext.pdf. Accessed 10 April 2014.
