# Supplementary material for: Indicators of the Statuses of Amphibian Populations and Their Potential for Exposure to Atrazine in Four Midwestern U.S. Conservation Areas
Source: PLoS One. 2014 Sep 12;9(9):e107018. doi: 10.1371/journal.pone.0107018 (PMC4162561; doi:10.1371/journal.pone.0107018)
Supplement: Figure S3 — Estimated application rates for atrazine in the conterminous United States and the estimated percentage applied to corn. (DOC) [file pone.0107018.s003.doc]

**Supporting Information**


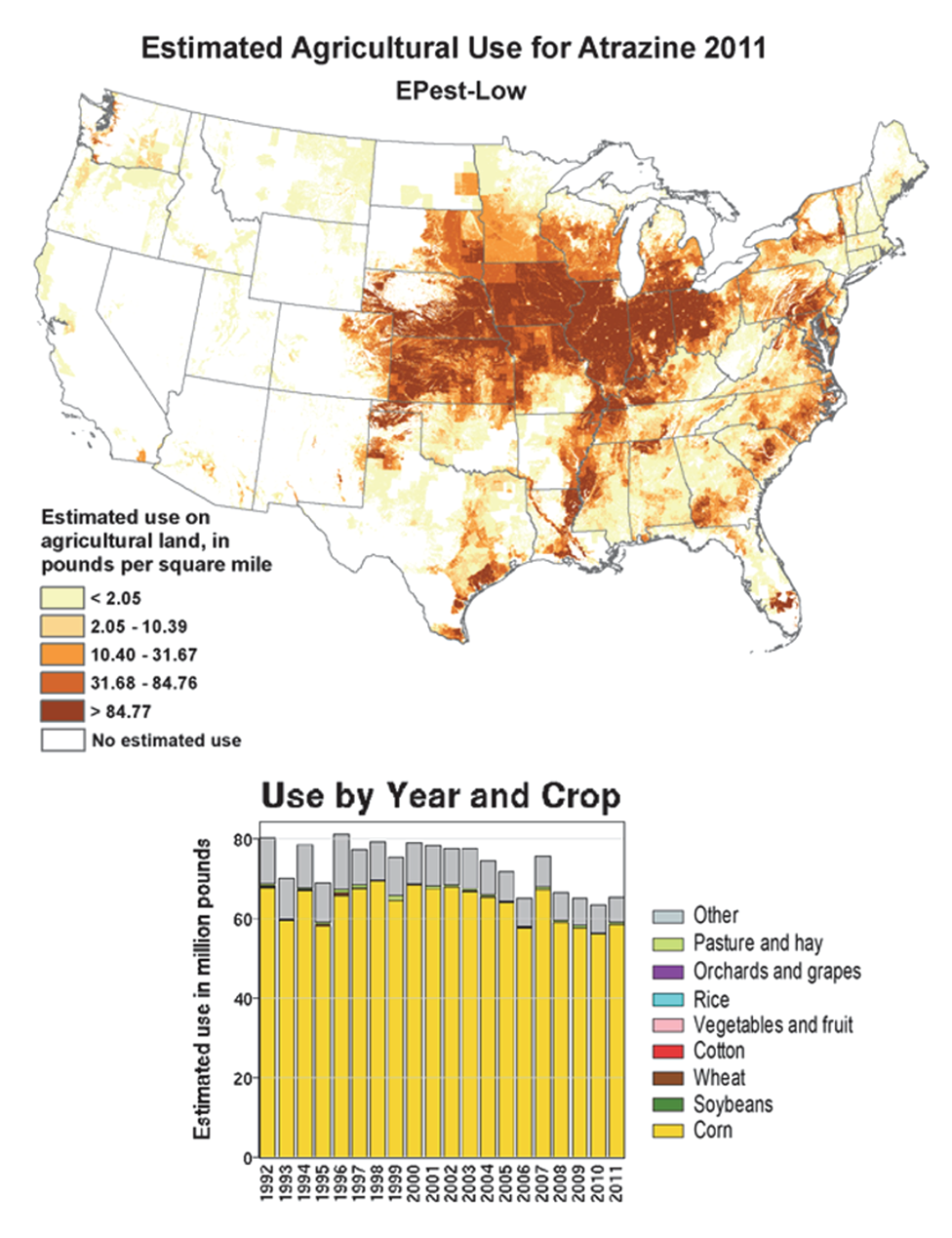


**Figure S3.** **Estimated application rates for atrazine in the conterminous United States during 2011 and the estimated percentage applied to corn from 1992–2011.**

Available: http://water.usgs.gov/nawqa/pnsp/usage/maps/show_map.php?year=2011&map=ATRAZINE&hilo=L. Accessed 10 April 2014.
