# Supplementary material for: Indicators of the Statuses of Amphibian Populations and Their Potential for Exposure to Atrazine in Four Midwestern U.S. Conservation Areas
Source: PLoS One. 2014 Sep 12;9(9):e107018. doi: 10.1371/journal.pone.0107018 (PMC4162561; doi:10.1371/journal.pone.0107018)
Supplement: Figure S4 — Acres of corn planted in the United States from 1993–2013. (DOC) [file pone.0107018.s004.doc]

**Supporting Information**


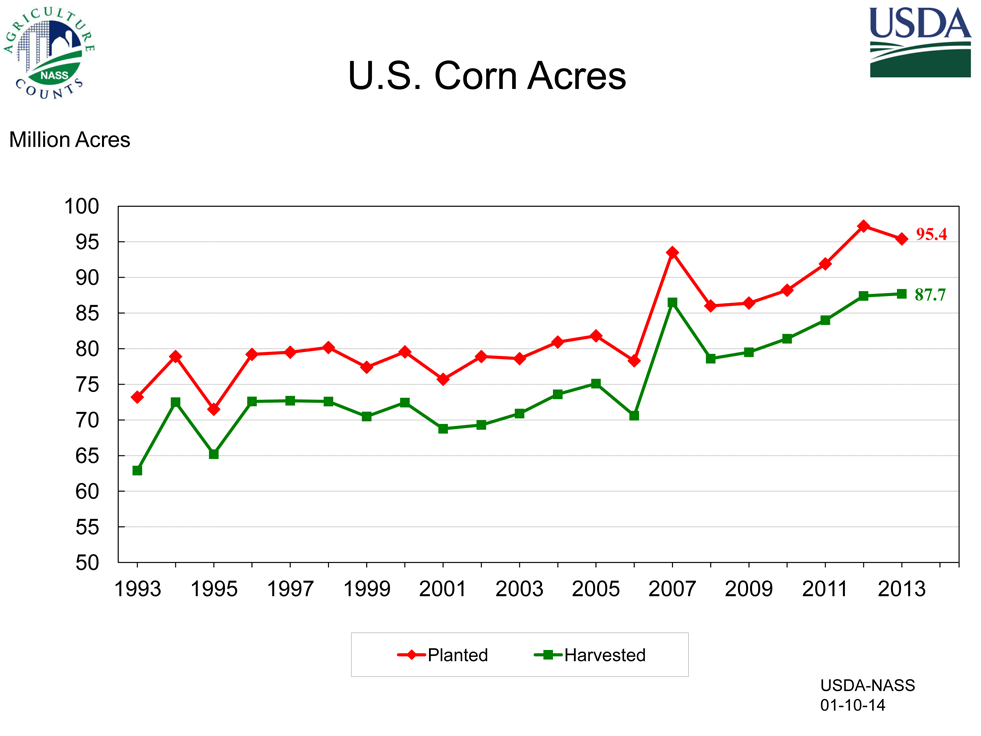


**Figure S4.** **Acres of corn planted in the United States from 1993−2013.**

Available: http://www.nass.usda.gov/Charts_and_Maps/Field_Crops/cornac.asp. Accessed 1 March 2014.
