# Supplementary material for: Indicators of the Statuses of Amphibian Populations and Their Potential for Exposure to Atrazine in Four Midwestern U.S. Conservation Areas
Source: PLoS One. 2014 Sep 12;9(9):e107018. doi: 10.1371/journal.pone.0107018 (PMC4162561; doi:10.1371/journal.pone.0107018)
Supplement: Figure S5 — Map of study areas and associated Level-III Ecoregions. (DOC) [file pone.0107018.s005.doc]

**Supporting Information**


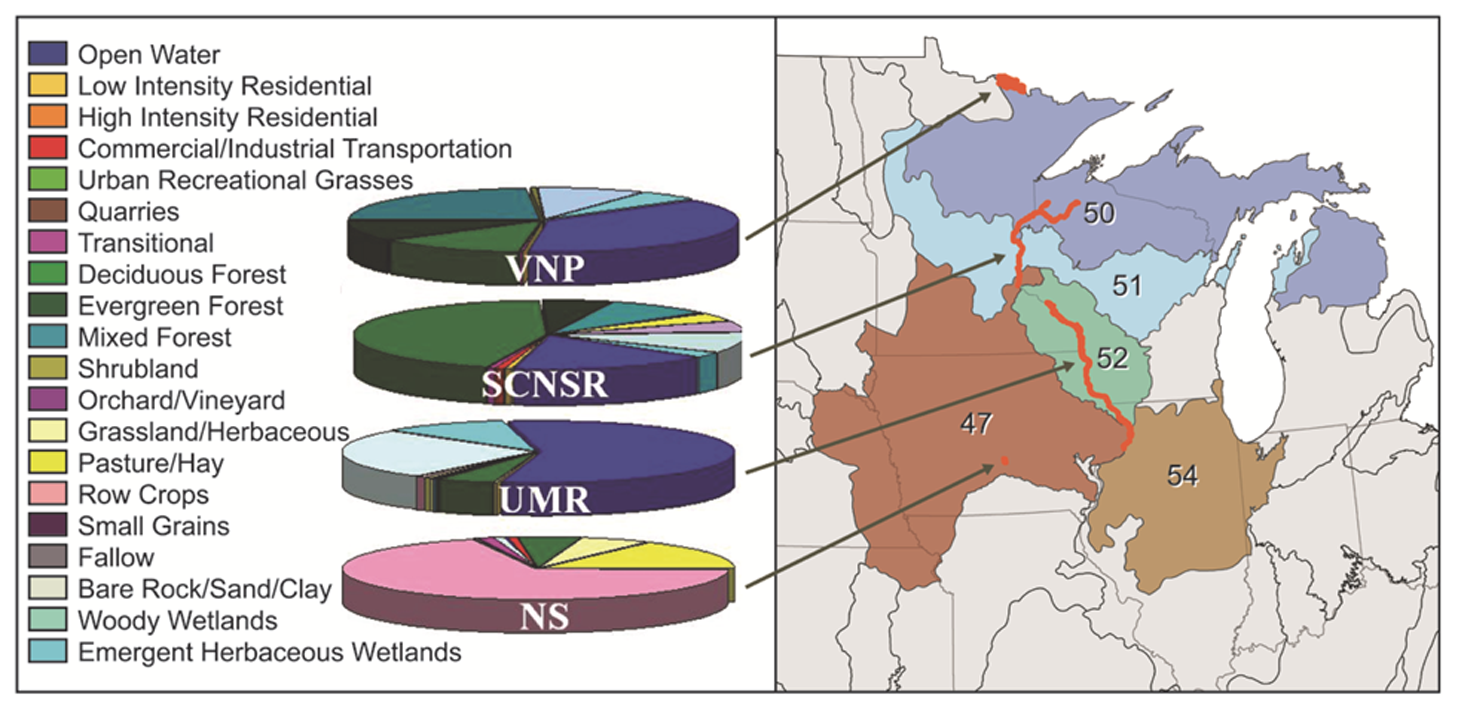


**Figure S5.** **Map of study areas and associated Level-III Ecoregions.**

47 = Western Corn Belt Plains; 50 = Northern Lakes and Forests; 51 = North Central Hardwood Forests; 52 = Driftless Area; 54 = Central Corn Belt Plains (Table S1). Inset describes the proportion of land-cover classes in each study area based upon the 2001 National Land Cover Database (http://www.mrlc.gov/nlcd2001.php). NS = the Neal Smith National Wildlife Refuge; UMR = the Upper Mississippi National Wildlife and Fish Refuge; SCNSR = the St. Croix National Scenic Riverway; VNP = Voyageurs National Park. Note: the agricultural categories shown in the NS have been restored to grasslands since 2000.
