# Supplementary material for: Indicators of the Statuses of Amphibian Populations and Their Potential for Exposure to Atrazine in Four Midwestern U.S. Conservation Areas
Source: PLoS One. 2014 Sep 12;9(9):e107018. doi: 10.1371/journal.pone.0107018 (PMC4162561; doi:10.1371/journal.pone.0107018)
Supplement: Figure S6 — Boxplots of proportions of metamorphs with gross external deformities per site per conservation area and year. (DOC) [file pone.0107018.s006.doc]

**Supporting Information**


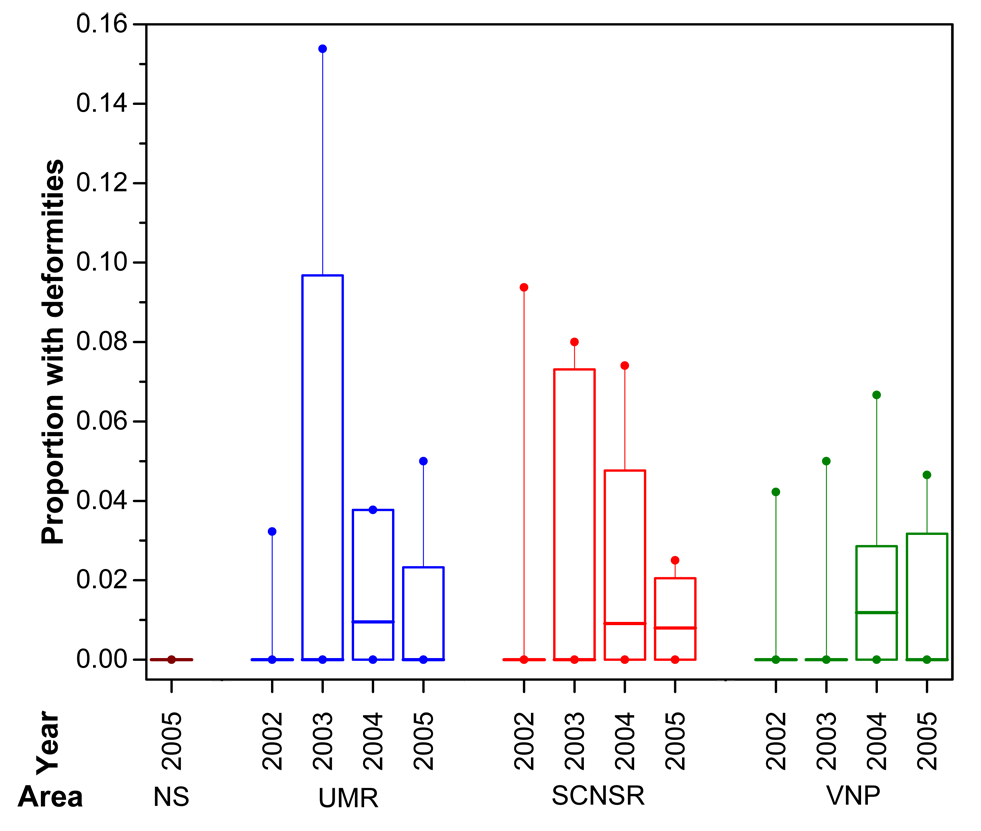


**Figure S6. Boxplots of proportions of metamorphs with gross external deformities per site per conservation area and year.**

Only includes sites where we caught and assessed at least 20 metamorphs, regardless of species. NS = Neal Smith National Wildlife Refuge; UMR = Upper Mississippi River National Wildlife and Fish Refuge; SCNSR = St. Croix National Scenic Riverway; VNP = Voyageurs National Park. Boxes indicate the interquartile range and capture the middle 50% of the data. Thick lines inside boxes indicate the median value. Vertical lines attached to boxes extend to the maximum and minimum values.
