# Supplementary material for: Indicators of the Statuses of Amphibian Populations and Their Potential for Exposure to Atrazine in Four Midwestern U.S. Conservation Areas
Source: PLoS One. 2014 Sep 12;9(9):e107018. doi: 10.1371/journal.pone.0107018 (PMC4162561; doi:10.1371/journal.pone.0107018)
Supplement: Figure S7 — Median triazine concentrations and the percent of Lithobates pipiens metamorphs with gross external deformities. (DOC) [file pone.0107018.s007.doc]

**Supporting Information**


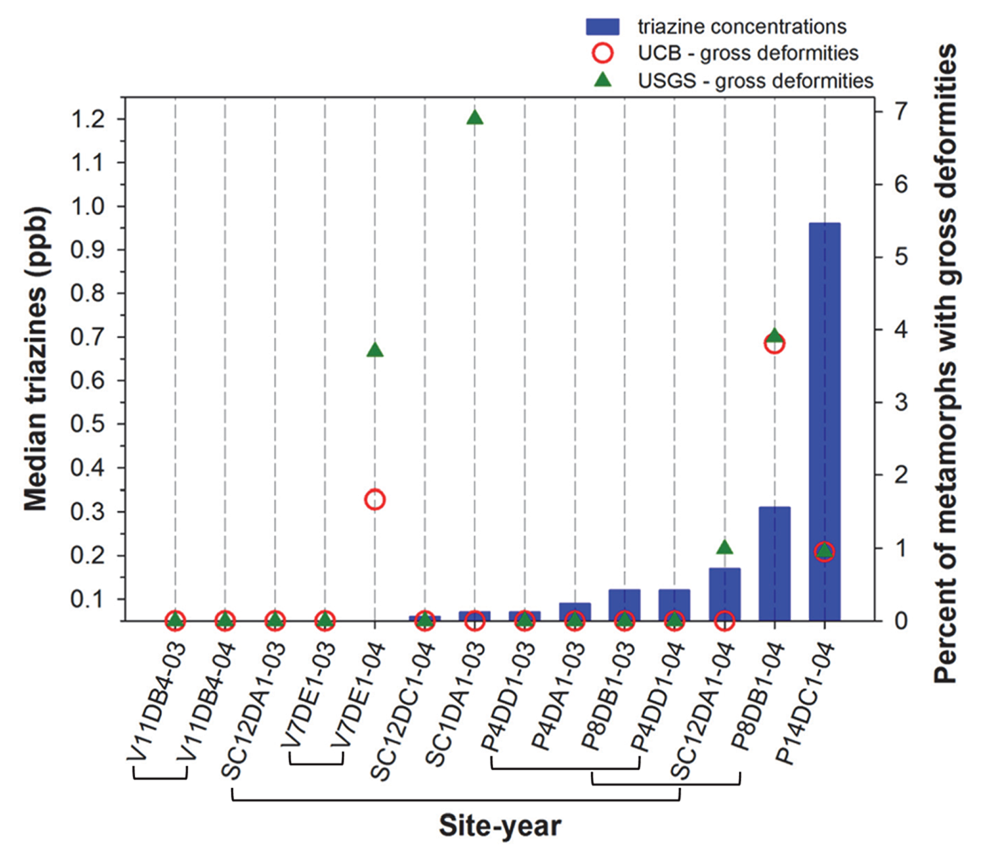


**Figure S7. Median triazine concentrations and the percent of *Lithobates pipiens* metamorphs with gross external deformities collected from individual breeding sites for assessments of trematode infections.**

UCB = University of California, Berkeley. USGS = U.S. Geological Survey. Total numbers of *L. pipiens* metamorphs assessed per site by UCB were: 50, 52, 50, 50, 54, 51, 50, 50, 50, 50, 50, 50, 50, and 50, respectively, from left to right on the x axis. Total numbers of metamorphs assessed by USGS were: 105, 102, 104, 101, 54, 51, 101, 100, 100, 100, 107, 101, 103, and 105, respectively. Sites identified with a V, SC, or P were in Voyageurs National Park, the St. Croix National Scenic Riverway, and the Upper Mississippi River National Wildlife and Fish Refuge, respectively. Triazine concentrations at sites with no vertical bars were below the detection limit (0.050 ppb) of the enzyme-linked immunosorbent assay used to analyze water samples. Lines below site names connect the same site surveyed in both years (2003 [-03] and 2004 [-04]).
