# Supplementary material for: Indicators of the Statuses of Amphibian Populations and Their Potential for Exposure to Atrazine in Four Midwestern U.S. Conservation Areas
Source: PLoS One. 2014 Sep 12;9(9):e107018. doi: 10.1371/journal.pone.0107018 (PMC4162561; doi:10.1371/journal.pone.0107018)
Supplement: Figure S8 — Comparisons of triazine concentrations in water samples from amphibian breeding sites and main channel sites in the UMR. (DOC) [file pone.0107018.s008.doc]

**Supporting Information**


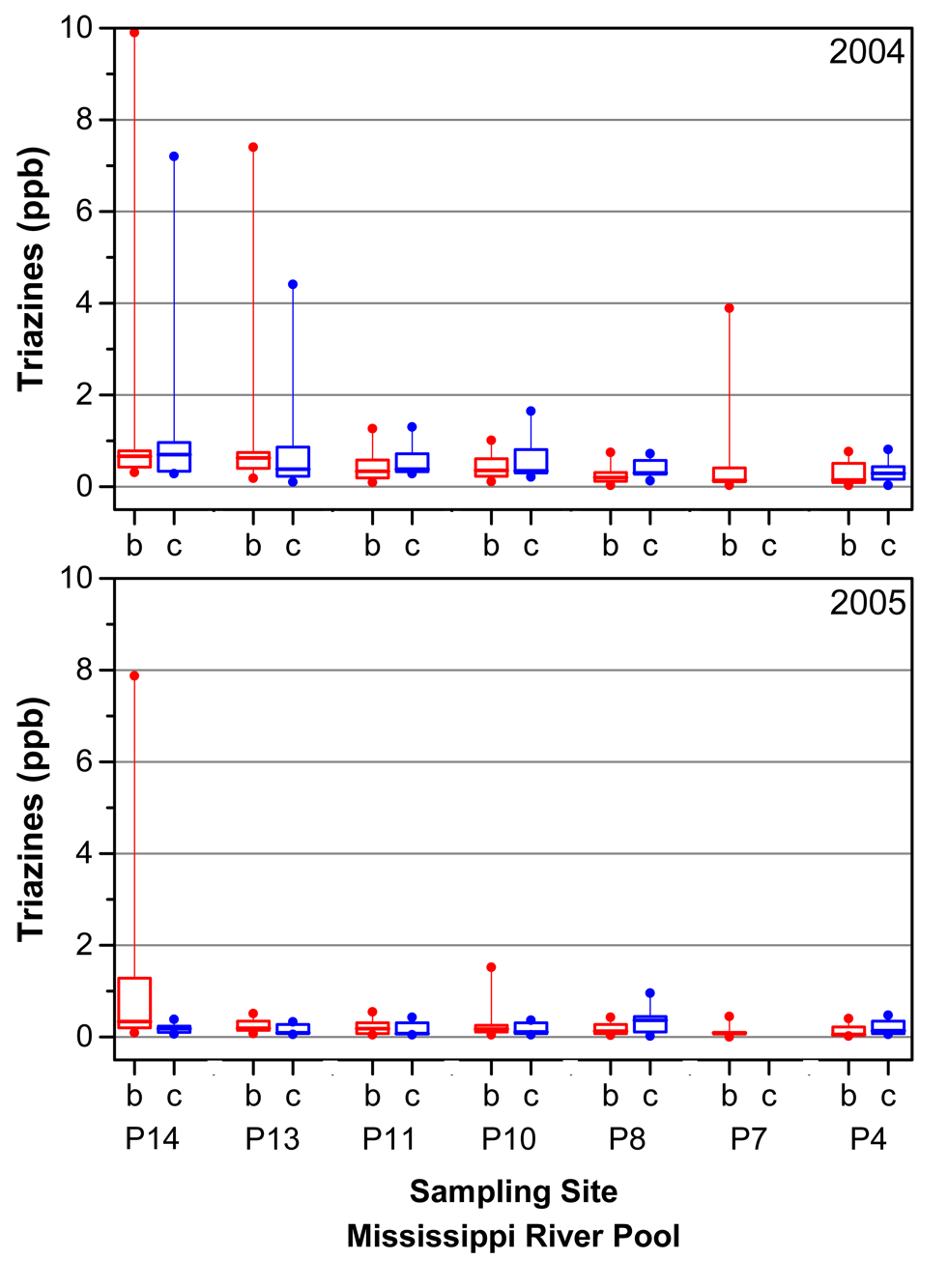


**Figure S8.** **Comparisons of triazine concentrations in water samples from amphibian breeding sites (b) and main channel sites (c) in the Upper Mississippi River National Wildlife and Fish Refuge for 2004 and 2005.**

Pools (P) were the individual pools formed by the lock-and-dam system on the Mississippi River. We measured triazine concentrations via an enzyme-linked immunosorbent assay designed to detect atrazine. Boxes indicate the interquartile range and capture the middle 50% of the data. Thick lines inside boxes indicate the median value. Vertical lines attached to boxes extend to the maximum and minimum values. We did not measure triazine concentrations for the channel site in Pool 7 in 2004 or 2005.
