# Supplementary material for: Indicators of the Statuses of Amphibian Populations and Their Potential for Exposure to Atrazine in Four Midwestern U.S. Conservation Areas
Source: PLoS One. 2014 Sep 12;9(9):e107018. doi: 10.1371/journal.pone.0107018 (PMC4162561; doi:10.1371/journal.pone.0107018)
Supplement: Figure S9 — Linear regressions on results from ELISA and LCMS analyses of water samples collected from the UMR, SCNSR, and VNP. (DOC) [file pone.0107018.s009.doc]

**Supporting Information**


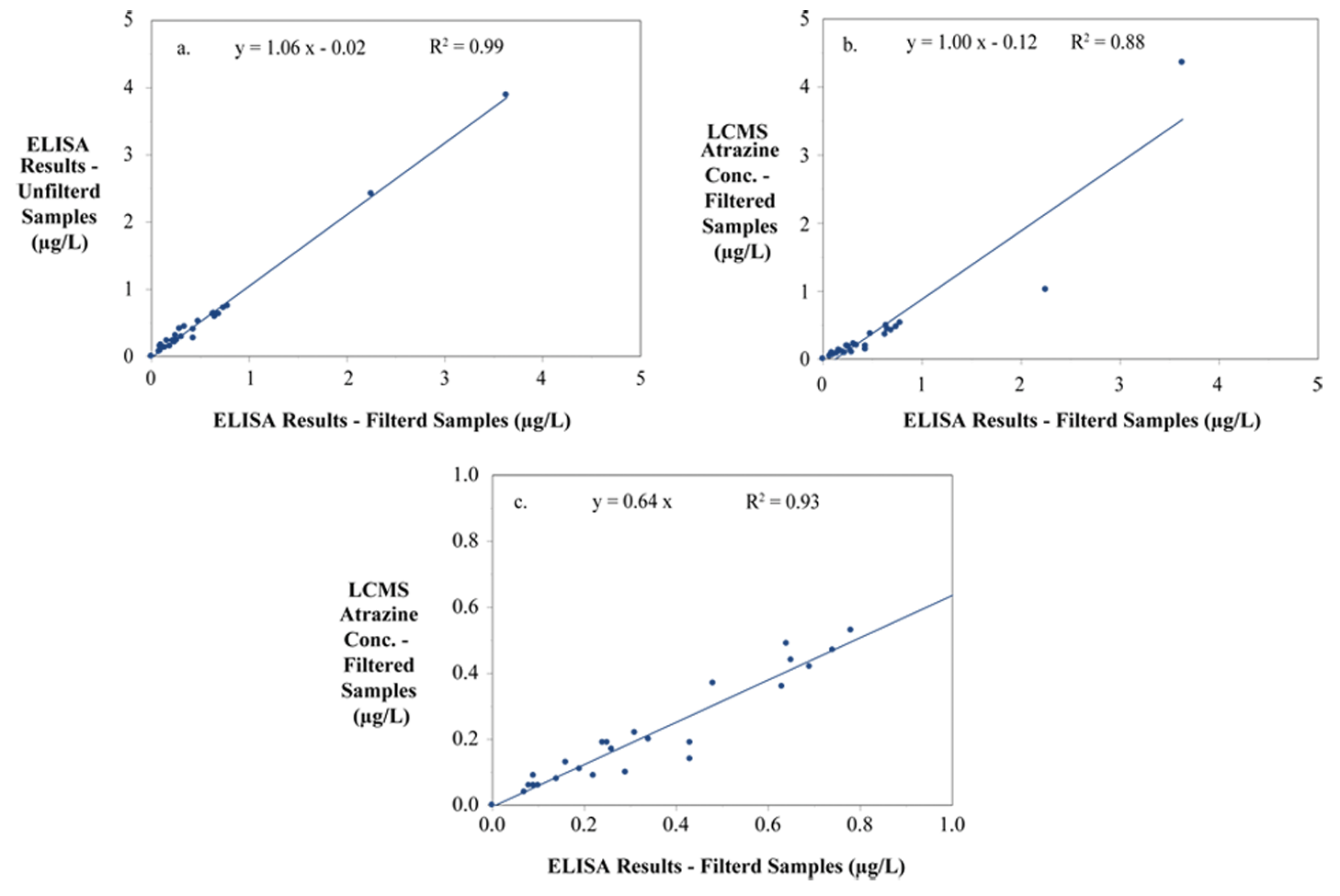


**Figure S9. Linear regressions on results from ELISA and LCMS analyses of water samples collected from 2003 to 2005 in the Upper Mississippi River National Wildlife and Fish Refuge, the St. Croix National Scenic Riverway, and Voyageurs National Park.**

Samples were analyzed via an enzyme-linked immunosorbent assay (ELISA) designed to detect atrazine (but which also could have detected other triazines) and liquid chromatography/mass spectrometry (LCMS). a) ELISA results for filtered samples versus ELISA results for the same samples unfiltered. b) LCMS results for filtered samples versus ELISA results for filtered samples. c) LCMS results for samples with LCMS concentrations below 1.0 µg/L versus ELISA results for filtered samples.
