# Supplementary material for: Indicators of the Statuses of Amphibian Populations and Their Potential for Exposure to Atrazine in Four Midwestern U.S. Conservation Areas
Source: PLoS One. 2014 Sep 12;9(9):e107018. doi: 10.1371/journal.pone.0107018 (PMC4162561; doi:10.1371/journal.pone.0107018)
Supplement: Figure S10 — Total seasonal monthly precipitation across the region containing our study areas from 2003 to 2005. (DOC) [file pone.0107018.s010.doc]

**Supporting Information**

**
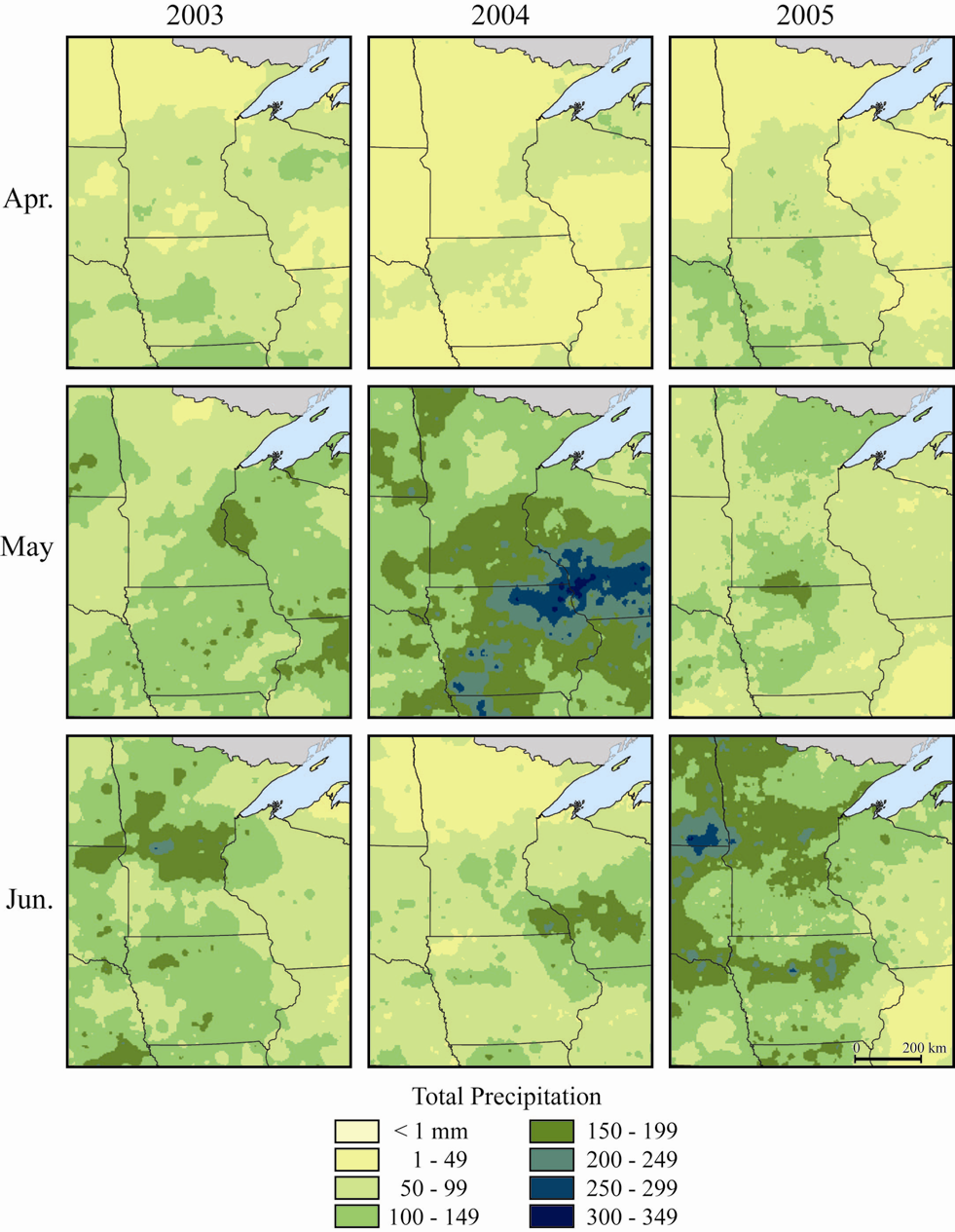
**

**Figure S10.** **Total seasonal monthly precipitation across the region containing our study areas from 2003 to 2005.**

Mapped with data produced by the Parameter-elevation Regressions on Independent Slopes Model [1] available from the PRISM Climate Group archive (http://www.prism.oregonstate.edu. Accessed 28 September 2010). Maps are depicted in an Albers equal-area projection at 4-km spatial resolution.

**References**

1. Daly C, Kittel TGF, McNab A, Gibson WP, Royle JA, Nychka D, Parzybok T, Rosenbloom N, Taylor G (2000) Development of a 103-year high-resolution climate data set for the conterminous United States. In: Proceedings of the 12^th^ American Meteorological Society, Conference on Applied Climatology. Asheville, North Carolina. USA. 8–11 May 2000. pp 249–252.
