# Supplementary material for: Indicators of the Statuses of Amphibian Populations and Their Potential for Exposure to Atrazine in Four Midwestern U.S. Conservation Areas
Source: PLoS One. 2014 Sep 12;9(9):e107018. doi: 10.1371/journal.pone.0107018 (PMC4162561; doi:10.1371/journal.pone.0107018)
Supplement: Figure S12 — Percent snow cover for a 385 km2 landscape block centered on the NS from 2003 to 2005. (DOC) [file pone.0107018.s012.doc]

**Supporting Information**


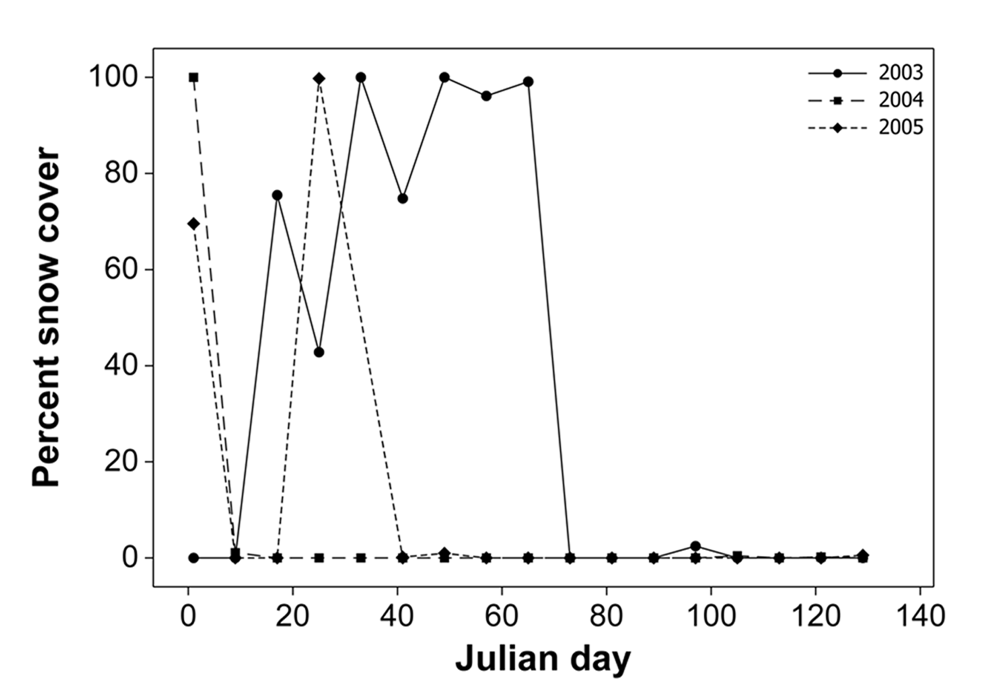


**Figure S12.** **Percent snow cover for a 385-km^2^ landscape block centered on the Neal Smith National Wildlife Refuge from 2003 to 2005**.

Estimated with time-series data from the Terra Moderate-Resolution Imaging Spectroradiometer (MODIS) sensor (MOD10 500-m resolution product; [1]). This landscape block includes the catchment that drains into the Neal Smith NWR, as well as the surrounding airshed.

**References**

1. Hall DK, Riggs GA, Salomonson VV, DiGirolamo NE, Bayr KJ (2002) MODIS snow-cover products. Remote Sens Environ 83: 181–194.
