# Supplementary material for: Indicators of the Statuses of Amphibian Populations and Their Potential for Exposure to Atrazine in Four Midwestern U.S. Conservation Areas
Source: PLoS One. 2014 Sep 12;9(9):e107018. doi: 10.1371/journal.pone.0107018 (PMC4162561; doi:10.1371/journal.pone.0107018)
Supplement: Figure S13 — The Normalized Difference Vegetation Index for corn in a 385 km2 landscape block centered on the NS from 2003 to 2005. (DOC) [file pone.0107018.s013.doc]

**Supporting Information**


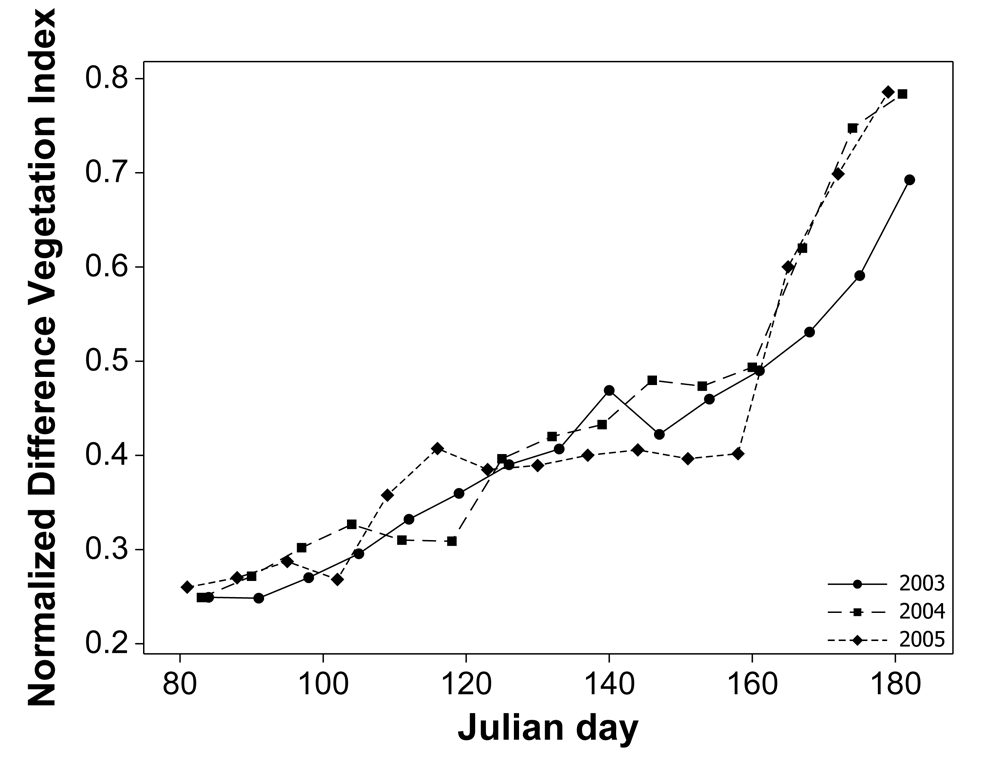


**Figure S13. The Normalized Difference Vegetation Index for corn in a 385-km^2^ landscape block centered on the Neal Smith National Wildlife Refuge from 2003 to 2005**.

Estimated from 250-m resolution eMODIS data [1] obtained from the USGS’ Earth Resources Observation and Science Center. This NDVI (Normalized Difference Vegetation Index) product highlights the production and senescence of green biomass in seven-day time steps. NDVI values are unitless and can range from -1.0 to 1.0, with higher values indicating higher green biomass. Vegetation phenology can be characterized by mapping NDVI values over the course of a year. Major inflection points in the curve are associated with greenup and senescence events. Here we focus on annual timing of the start-of-season inflection of the phenology curve for NDVI values averaged across all pixels identified as corn (National Agricultural Statistics Service, http://www.nass.usda.gov/research/Cropland/SARS1a.htm) in the landscape surrounding the Neal Smith National Wildlife Refuge. Snow is present early in the year, resulting in markedly low NDVI values. Following snowmelt, greenup is noticeable in April, but the rates of greenup (slopes of the curves) at the beginning of the season vary among years.

**References**

1. Jenkerson C, Maiersperger T, Schmidt G (2010) eMODIS–a user-friendly data source. Open-File Report 2010—1055.US Geological Survey, Sioux Falls, South Dakota.
