# Supplementary material for: Indicators of the Statuses of Amphibian Populations and Their Potential for Exposure to Atrazine in Four Midwestern U.S. Conservation Areas
Source: PLoS One. 2014 Sep 12;9(9):e107018. doi: 10.1371/journal.pone.0107018 (PMC4162561; doi:10.1371/journal.pone.0107018)
Supplement: Figure S14 — Relative distributions of lands planted in corn within a 385-km2 landscape block centered on the NS from 2003 to 2005. (DOC) [file pone.0107018.s014.doc]

**Supporting Information**


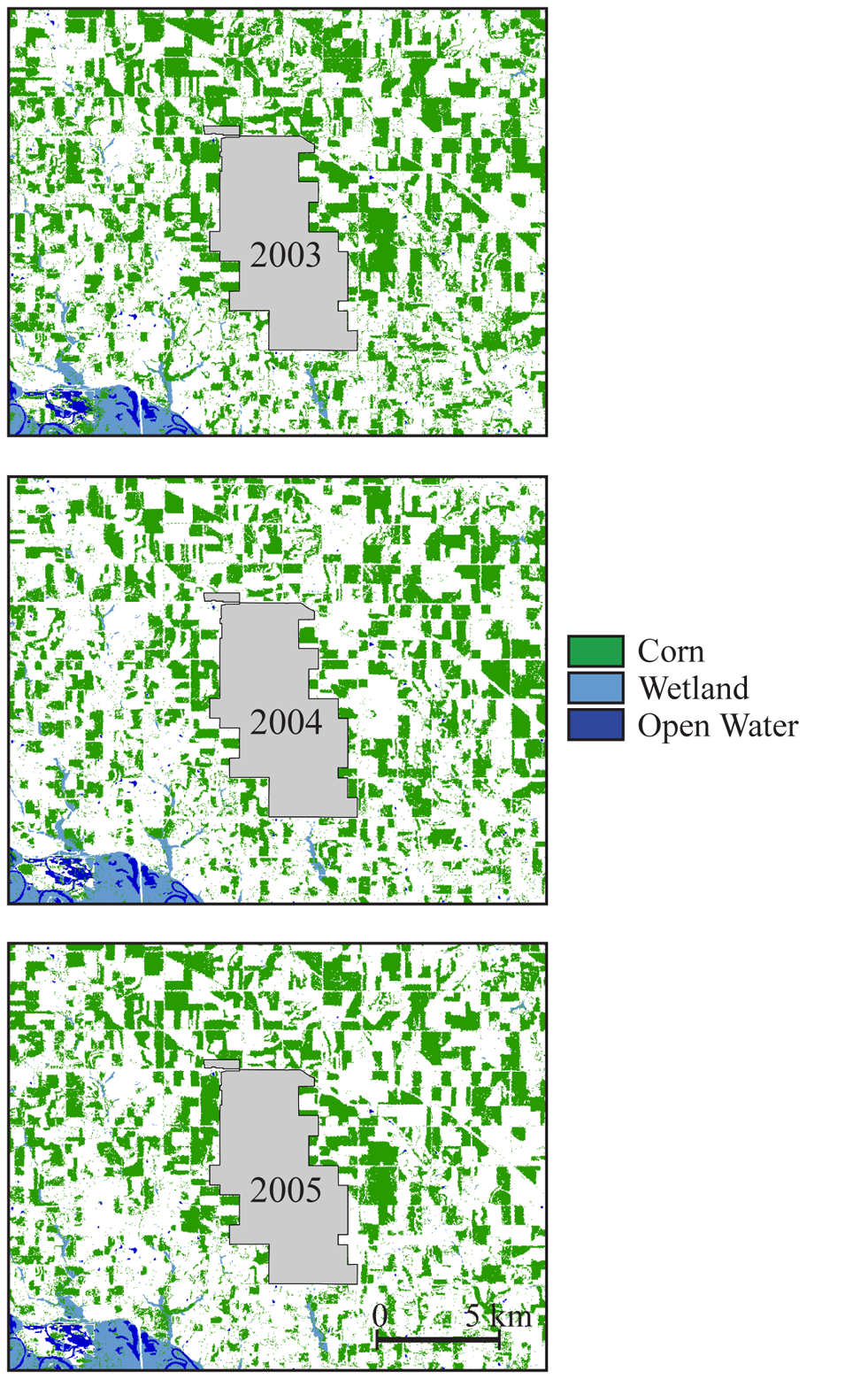


**Figure S14.** **Relative distributions of lands planted in corn within a 385-km^2^ landscape block centered on the Neal Smith National Wildlife Refuge from 2003 to 2005**.

Mapped with data from annual cropland maps developed by the U.S. Department of Agriculture’s National Agricultural Statistics Service based upon data from the Landsat Thematic Mapper sensor (http://www.nass.usda.gov/research/Cropland/SARS1a.htm). The extent of landscape shown in this figure includes the catchment that drains into the Neal Smith National Wildlife Refuge (the polygon containing the year label), as well as the surrounding airshed.
