# Supplementary material for: Indicators of the Statuses of Amphibian Populations and Their Potential for Exposure to Atrazine in Four Midwestern U.S. Conservation Areas
Source: PLoS One. 2014 Sep 12;9(9):e107018. doi: 10.1371/journal.pone.0107018 (PMC4162561; doi:10.1371/journal.pone.0107018)
Supplement: Table S1 — Descriptions of the Level-III Ecoregions in which our study areas were located. (DOC) [file pone.0107018.s015.doc]

**Supporting Information**

**Table S1.** Descriptions of the Level-III Ecoregions in which our study areas were located (abridged from [1]). Numbers in parentheses = the Omernik Level-III Ecoregion code [2].

| **Level-III Ecoregion** | **Description** |
| --- | --- |
| Western Corn Belt Plains  (47) | Once covered with tallgrass prairie, over 75% of the Western Corn Belt Plains now is used for cropland agriculture and much of the remainder is in forage for livestock. A combination of nearly level to gently rolling glaciated till plains and hilly loess plains, an average annual precipitation range of 63 to 89 cm, much of which occurs in the growing season, and fertile, warm, moist soils make this one of the most productive areas for corn and soybeans in the world. Major environmental concerns in the region include surface and groundwater contamination from fertilizer and pesticide applications as well as impacts from concentrated livestock production. |
| Northern Lakes and Forests  (50) | The Northern Lakes and Forests is a region of nutrient poor glacial soils, coniferous and northern hardwood forests, undulating till plains, morainal hills, broad lacustrine basins, and extensive sandy outwash plains. Soils in this ecoregion are thicker than in those to the north and generally lack the arability of soils in adjacent ecoregions to the south. The numerous lakes that dot the landscape are clearer and less productive than those in ecoregions to the south. |
| North Central Hardwood Forests  (51) | The North Central Hardwood Forests is transitional between the predominantly forested Northern Lakes and Forests to the north and the agricultural ecoregions to the south. Land cover/use in this ecoregion is a mosaic of forests, wetlands and lakes, cropland agriculture, pasture, and dairy operations. |
| Driftless Area  (52) | The hilly uplands of the Driftless Area easily distinguish it from surrounding ecoregions. Much of the area is a deeply dissected, loess-capped, bedrock dominated plateau. The region is also called the Paleozoic Plateau because the landscape’s appearance is a result of erosion through rock strata of Paleozoic age. Although there is evidence of glacial drift in the region, the glacial deposits had little impact on the landscape compared to their subduing influences in adjacent ecoregions. Livestock and dairy farming are major land uses and have had a substantial impact on water quality. |
| Central Corn Belt Plains  (54) | Extensive prairies intermixed with oak-hickory forests were native to the glaciated plains of the Central Corn Belt Plains and a stark contrast to the hardwood forests that grew on the drift plains of ecoregions to the east. Beginning in the nineteenth century, the natural vegetation gradually was replaced by agriculture. Farms are extensive on the dark, fertile soils of this ecoregion and mainly produce corn and soybeans. Cattle, sheep, poultry, and especially hogs also are raised. Agriculture has affected surface-water chemistry, turbidity, and habitat. |

**References**

1. Wiken E, Nava FJ, Griffith G (2011) North American Terrestrial Ecoregions – Level III. Commission for Environmental Cooperation, Montreal, Canada.

2. Omernik JM (1987) Ecoregions of the conterminous United States. Annals Assoc Amer Geog 77: 118–125.
