# Supplementary material for: Indicators of the Statuses of Amphibian Populations and Their Potential for Exposure to Atrazine in Four Midwestern U.S. Conservation Areas
Source: PLoS One. 2014 Sep 12;9(9):e107018. doi: 10.1371/journal.pone.0107018 (PMC4162561; doi:10.1371/journal.pone.0107018)
Supplement: Table S2 — Summary of wetlands surveyed during daytime amphibian surveys. (DOC) [file pone.0107018.s016.doc]

**Supporting Information**

**Table S2.** Summary of wetlands surveyed during daytime amphibian surveys in the Upper Mississippi National Wildlife and Fish Refuge (UMR), the St. Croix National Scenic Riverway (SCNSR), Voyageurs National Park (VNP), and the Neal Smith National Wildlife Refuge (NS) from 2002 to 2005.

| **Management**  **Area** | **# of wetlands surveyed - v1** | **# of wetlands surveyed - v2** | **# of wetlands surveyed - v3** | **total # of wetlands surveyed** |
| --- | --- | --- | --- | --- |
| UMR 2002 | 4 | 39 | 52 | 72 |
| SCNSR 2002 | 25 | 52 | 57 | 59 |
| VNP 2002 | 22 | 43 | 47 | 47 |
| UMR 2003 | 37 | 29 | 58 | 58 |
| SCNSR 2003 | 41 | 52 | 58 | 62 |
| VNP 2003 | 29 | 44 | 53 | 56 |
| NS 2004 | 15 | 0 | 16 | 16 |
| UMR 2004 | 19 | 46 | 29 | 54 |
| SCNSR 2004 | 21 | 45 | 62 | 64 |
| VNP 2004 | 23 | 48 | 49 | 55 |
| NS 2005 | 17 | 17 | 11 | 17 |
| UMR 2005 | 2 | 20 | 20 | 20 |
| SCNSR 2005 | 2 | 18 | 19 | 19 |
| VNP 2005 | 23 | 25 | 26 | 26 |

We conducted sampling visits 1 (v1), 2 (v2), and 3 (v3) during the early, mid-, and latter portions of the amphibian reproduction season, respectively. The number of wetlands differed across sampling visits within each year primarily due to resource and logistical constraints. Total numbers of wetlands differed from numbers of wetlands sampled across visits within a season because all wetlands were not necessarily sampled the same number of times each season.
