# Supplementary material for: Indicators of the Statuses of Amphibian Populations and Their Potential for Exposure to Atrazine in Four Midwestern U.S. Conservation Areas
Source: PLoS One. 2014 Sep 12;9(9):e107018. doi: 10.1371/journal.pone.0107018 (PMC4162561; doi:10.1371/journal.pone.0107018)
Supplement: Table S4 — Summary of the number of wetlands surveyed for amphibian deformities. (DOC) [file pone.0107018.s018.doc]

**Supporting Information**

**Table S4.** Summary of the number of wetlands surveyed opportunistically for gross external deformities in the Upper Mississippi National Wildlife and Fish Refuge (UMR), the St. Croix National Scenic Riverway (SCNSR), Voyageurs National Park (VNP), and the Neal Smith National Wildlife Refuge (NS) from 2002 to 2005.

| **Management**  **Area** | **# of wetlands** |
| --- | --- |
| UMR 2002 | 50 |
| SCNSR 2002 | 44 |
| VNP 2002 | 35 |
| UMR 2003 | 39 |
| SCNSR 2003 | 47 |
| VNP 2003 | 35 |
| NS 2004 | 8 |
| UMR 2004 | 24 |
| SCNSR 2004 | 35 |
| VNP 2004 | 38 |
| NS 2005 | 2 |
| UMR 2005 | 19 |
| SCNSR 2005 | 19 |
| VNP 2005 | 26 |
