# Supplementary material for: Indicators of the Statuses of Amphibian Populations and Their Potential for Exposure to Atrazine in Four Midwestern U.S. Conservation Areas
Source: PLoS One. 2014 Sep 12;9(9):e107018. doi: 10.1371/journal.pone.0107018 (PMC4162561; doi:10.1371/journal.pone.0107018)
Supplement: Table S5 — Output from PRESENCE ranking the top occupancy models for the NS. (DOC) [file pone.0107018.s019.doc]

**Supporting Information**

**Table S5.** Output from PRESENCE [1] ranking the top occupancy models for the Neal Smith National Wildlife Refuge from 2004 to 2005*.*

| **Models** | **AIC** | **ΔAIC** | **AIC weight** | **Model likelihood** | **Parameters** |
| --- | --- | --- | --- | --- | --- |
| ***Pseudacris maculata*** | | | | | |
| ψ()γ()ε()ρ(observer and method) | 323.32 | 0 | 1 | 1 | 6 |
| ***Lithobates pipiens*** | | | | | |
| ψ()γ()ε()ρ() | 192.19 | 0 | 0.5805 | 1 | 4 |
| ψ()γ()ε()ρ(observer and method) | 192.84 | 0.65 | 0.4195 | 0.7225 | 6 |

AIC = Akaike’s Information Criterion and ΔAIC = the difference in model AIC value compared to the AIC value of the first model listed. AIC weight = the model likelihood/total of all model likelihoods and is a measure of support for each model being the “best” model. Model likelihood = model AIC weight/AIC weight of the top model listed. Parameters = number of parameters used to fit the model. ψ = estimate of occupancy probability, γ = estimate of colonization probability, ε = estimate of extinction probability, and ρ = estimate of detection probability. Observer (novice or experienced) and method (sampling method) were important covariates for estimating ρ.

**References**

1. Hines JE (2006) PRESENCE software to estimate patch occupancy and related parameters. U.S. Geological Survey, Patuxent Wildlife Research Center. Available: http://www.mbr-pwrc.usgs.gov/software/presence.html. Accessed 24 September 2013.
