# Supplementary material for: Indicators of the Statuses of Amphibian Populations and Their Potential for Exposure to Atrazine in Four Midwestern U.S. Conservation Areas
Source: PLoS One. 2014 Sep 12;9(9):e107018. doi: 10.1371/journal.pone.0107018 (PMC4162561; doi:10.1371/journal.pone.0107018)
Supplement: Table S6 — Output from PRESENCE ranking the top occupancy models for the UMR. (DOC) [file pone.0107018.s020.doc]

**Supporting Information**

**Table S6.** Output from PRESENCE [1] ranking the top occupancy models for the Upper Mississippi National Wildlife and Fish Refuge from 2002 to 2005*.*

| **Models** | **AIC** | **ΔAIC** | **AIC weight** | **Model likelihood** | **Parameters** |
| --- | --- | --- | --- | --- | --- |
| ***Anaxyrus americanus*** | | | | | |
| ψ()γ()ε()ρ() | 212.29 | 0 | 0.4241 | 1 | 4 |
| ψ()γ()ε()ρ(observer and method) | 212.58 | 0.29 | 0.3668 | 0.865 | 6 |
| ψ(mean patch size of habitat)γ()ε()ρ(observer and method) | 214.35 | 2.06 | 0.1514 | 0.357 | 7 |
| ψ(% crops, mean patch size of habitat)γ()ε()ρ(observer and method) | 216.28 | 3.99 | 0.0577 | 0.136 | 8 |
| ***Hyla chrysoscelis/versicolor*^1^** | | | | | |
| ψ()γ()ε()ρ(observer and method) | 396.39 | 0 | 0.6857 | 1 | 6 |
| ψ(mean patch size of habitat)γ()ε()ρ(observer and method) | 397.95 | 1.56 | 0.3143 | 0.4584 | 7 |
| ψ()γ()ε()ρ()* | 404.99 | 8.6 | - | - | 4 |
| ***Lithobates clamitans*** |  |  |  |  |  |
| ψ()γ()ε()ρ() | 1485.32 | 0 | 1 | 1 | 4 |
| ***Lithobates pipiens*** | | | | | |
| ψ(hydroperiod, mean patch size of habitat)γ()ε()ρ(observer and method) | 1536.57 | 0 | 0.4002 | 1 | 9 |
| ψ(hydroperiod, % crops)γ()ε()ρ(observer and method) | 1537.85 | 1.28 | 0.2110 | 0.5273 | 9 |
| ψ(mean patch size of habitat)γ()ε()ρ(observer and method) | 1538.54 | 1.97 | 0.1495 | 0.3734 | 7 |
| ψ(% crops)γ()ε()ρ(observer and method) | 1539.65 | 3.08 | 0.0858 | 0.2144 | 7 |
| ψ()γ()ε()ρ(observer and method) | 1539.81 | 3.24 | 0.0792 | 0.1979 | 6 |
| ψ(% crops, mean patch size of habitat)γ()ε()ρ(observer and method) | 1539.94 | 3.37 | 0.0742 | 0.1854 | 8 |
| ψ()γ()ε()ρ()* | 1576.71 | 40.14 | - | - | 4 |

AIC = Akaike’s Information Criterion and ΔAIC = the difference in model AIC value compared to the AIC value of the first model listed. AIC weight = the model likelihood/total of all model likelihoods and is a measure of support for each model being the “best” model. Model likelihood = model AIC weight/AIC weight of the top model listed. Parameters = number of parameters used to fit the model. ψ = estimate of occupancy probability, γ = estimate of colonization probability, ε = estimate of extinction probability, and ρ = estimate of detection probability. Hydroperiod (ephemeral, semi-permanent, or permanent), % crops (% of croplands within the 4-km site buffer), mean patch size of habitat (mean patch size of land-cover types within the 4-km site buffer that was not cropland and was potential amphibian habitat), observer (novice or experienced), and method (sampling method) were important covariates for estimating the associated parameter.

**^1^** We could not distinguish between these two species visually. Thus, we combined all animals of these species that we detected into one complex for this analysis.

* The null model was > 5 ΔAIC, but met the other model-selection criteria and is listed for comparison.

**References**

1. Hines JE (2006) PRESENCE software to estimate patch occupancy and related parameters. U.S. Geological Survey, Patuxent Wildlife Research Center. Available: http://www.mbr-pwrc.usgs.gov/software/presence.html. Accessed 24 September 2013.
