# Supplementary material for: Indicators of the Statuses of Amphibian Populations and Their Potential for Exposure to Atrazine in Four Midwestern U.S. Conservation Areas
Source: PLoS One. 2014 Sep 12;9(9):e107018. doi: 10.1371/journal.pone.0107018 (PMC4162561; doi:10.1371/journal.pone.0107018)
Supplement: Table S7 — Output from PRESENCE ranking the top occupancy models for the SCNSR. (DOC) [file pone.0107018.s021.doc]

**Supporting Information**

**Table S7.** Output from PRESENCE [1] ranking the top occupancy models for the St. Croix National Scenic Riverway from 2002 to 2005*.*

| **Models** | **AIC** | **ΔAIC** | **AIC weight** | **Model likelihood** | **Parameters** |
| --- | --- | --- | --- | --- | --- |
| ***Anaxyrus americanus*** | | | | | |
| ψ((% crops)γ()ε()ρ(observer and method) | 614.75 | 0 | 1 | 1 | 7 |
| ***Pseudacris crucifer*** | | | | | |
| ψ(mean patch size of habitat)γ()ε()ρ(observer and method) | 512.31 | 0 | 1 | 1 | 7 |
| ***Pseudacris maculata*** | | | | | |
| ψ()γ()ε()ρ() | 781.86 | 0 | 1 | 1 | 4 |
| ***Lithobates clamitans*** | | | | | |
| ψ()γ()ε()ρ(observer and method) | 1525.39 | 0 | 1 | 1 | 6 |
| ψ()γ()ε()ρ()* | 1557.89 | 34.99 | - | - | 4 |
| ***Lithobates pipiens*** | | | | | |
| ψ(mean patch size of habitat)γ()ε()ρ(observer and method) | 734.01 | 0 | 0.7301 | 1 | 7 |
| ψ(% crops, mean patch size of habitat)γ()ε()ρ(observer and method) | 736 | 1.99 | 0.2699 | 0.3697 | 8 |
| ψ()γ()ε()ρ()* | 744.47 | 10.46 | - | - | 4 |
| ***Lithobates septentrionalis*** | | | | | |
| ψ()γ()ε()ρ() | 339.31 | 0 | 1 | 1 | 4 |
| ***Lithobates sylvaticus*** | | | | | |
| ψ()γ()ε()ρ(observer and method) | 1090.31 | 0 | 0.6112 | 1 | 6 |
| ψ(hydroperiod, mean patch size of habitat)γ()ε()ρ(observer and method) | 1092.99 | 2.68 | 0.1600 | 0.2618 | 9 |
| ψ(hydroperiod)γ()ε()ρ(observer and method) | 1093.31 | 3 | 0.1364 | 0.2231 | 8 |
| ψ()γ()ε()ρ() | 1094.09 | 3.78 | 0.0923 | 0.1511 | 4 |

AIC = Akaike’s Information Criterion and ΔAIC = the difference in model AIC value compared to the AIC value of the first model listed. AIC weight = the model likelihood/total of all model likelihoods and is a measure of support for each model being the “best” model. Model likelihood = model AIC weight/AIC weight of the top model listed. Parameters = number of parameters used to fit the model. ψ = estimate of occupancy probability, γ = estimate of colonization probability, ε = estimate of extinction probability, and ρ = estimate of detection probability. Hydroperiod (ephemeral, semi-permanent, or permanent), % crops (% of croplands within the 4-km site buffer), mean patch size of habitat (mean patch size of land-cover types within the 4-km site buffer that was not cropland and was potential amphibian habitat), observer (novice or experienced), and method (sampling method) were important covariates for estimating the associated parameter.

* The null model was > 5 ΔAIC, but met the other model-selection criteria and is listed for comparison.

**References**

1. Hines JE (2006) PRESENCE software to estimate patch occupancy and related parameters. U.S. Geological Survey, Patuxent Wildlife Research Center. Available: http://www.mbr-pwrc.usgs.gov/software/presence.html. Accessed 24 September 2013.
