# Supplementary material for: Indicators of the Statuses of Amphibian Populations and Their Potential for Exposure to Atrazine in Four Midwestern U.S. Conservation Areas
Source: PLoS One. 2014 Sep 12;9(9):e107018. doi: 10.1371/journal.pone.0107018 (PMC4162561; doi:10.1371/journal.pone.0107018)
Supplement: Table S8 — Output from PRESENCE ranking the top occupancy models for VNP. (DOC) [file pone.0107018.s022.doc]

**Supporting Information**

**Table S8.** Output from PRESENCE [1] ranking the top occupancy models for Voyageurs National Park from 2002 to 2005*.*

| **Models** | **AIC** | **ΔAIC** | **AIC weight** | **Model likelihood** | **Parameters** |
| --- | --- | --- | --- | --- | --- |
| ***Anaxyrus americanus*** | | | | | |
| ψ()γ()ε()ρ(observer and method) | 436.31 | 0 | 0.6376 | 1 | 6 |
| ψ(mean patch size of habitat)γ()ε()ρ(observer and method) | 437.44 | 1.13 | 0.3624 | 0.5684 | 7 |
| ψ()γ()ε()ρ()* | 461.73 | 25.42 | - | - | 4 |
| ***Hyla versicolor/chrysoscelis^1^*** | | | | | |
| ψ()γ()ε()ρ(observer and method) | 466.64 | 0 | 0.7261 | 1 | 6 |
| ψ(mean patch size of habitat)γ()ε()ρ(observer and method) | 468.59 | 1.95 | 0.2739 | 0.3772 | 7 |
| ψ()γ()ε()ρ()* | 481.87 | 24.63 | - | - | 4 |
| ***Pseudacris crucifer*** | | | | | |
| ψ(hydroperiod)γ()ε()ρ(observer and method) | 873.00 | 0 | 0.8131 | 1 | 8 |
| ψ()γ()ε()ρ(observer ,method) | 875.94 | 2.94 | 0.1869 | 0.2299 | 6 |
| ψ()γ()ε()ρ()* | 890.11 | 17.11 | - | - | 4 |
| ***Lithobates clamitans*** | | | | | |
| ψ()γ()ε()ρ(observer and method) | 862.69 | 0 | 1 | 1 | 6 |
| ψ()γ()ε()ρ()* | 869.64 | 6.95 | - | - | 4 |
| ***Lithobates pipiens*** | | | | | |
| ψ()γ()ε()ρ(observer and method) | 671.43 | 0 | 0.7140 | 1 | 6 |
| ψ(mean patch size of habitat)γ()ε()ρ(observer and method) | 673.26 | 1.83 | 0.2860 | 0.4005 | 7 |
| ψ()γ()ε()ρ()* | 680.51 | 9.06 | - | - | 4 |
| ***Lithobates septentrionalis*** | | | | | |
| ψ()γ()ε()ρ(observer and method) | 690.23 | 0 | 1 | 1 | 6 |
| ψ()γ()ε()ρ()* | 703.57 | 13.34 | - | - | 4 |
| ***Lithobates sylvaticus*** | | | | | |
| ψ()γ()ε()ρ() | 1582.07 | 0 | 1 | 1 | 4 |

AIC = Akaike’s Information Criterion and ΔAIC = the difference in model AIC value compared to the AIC value of the first model listed. AIC weight = the model likelihood/total of all model likelihoods and is a measure of support for each model being the “best” model. Model likelihood = model AIC weight/AIC weight of the top model listed. Parameters = number of parameters used to fit the model. ψ = estimate of occupancy probability, γ = estimate of colonization probability, ε = estimate of extinction probability, and ρ = estimate of detection probability. Hydroperiod (ephemeral, semi-permanent, or permanent), % crops (% of croplands within the 4-km site buffer), mean patch size of habitat (mean patch size of land-cover types within the 4-km site buffer that was not cropland and was potential amphibian habitat), observer (novice or experienced), and method (sampling method) were important covariates for estimating the associated parameter.

**^1^** We could not distinguish between these two species visually. Thus, we combined all animals of these species into one complex for this analysis.

* The null model was > 5 ΔAIC, but met the other model-selection criteria and is listed for comparison.

**References**

1. Hines JE (2006) PRESENCE software to estimate patch occupancy and related parameters. U.S. Geological Survey, Patuxent Wildlife Research Center. Available: http://www.mbr-pwrc.usgs.gov/software/presence.html. Accessed 24 September 2013.
