# Supplementary material for: Indicators of the Statuses of Amphibian Populations and Their Potential for Exposure to Atrazine in Four Midwestern U.S. Conservation Areas
Source: PLoS One. 2014 Sep 12;9(9):e107018. doi: 10.1371/journal.pone.0107018 (PMC4162561; doi:10.1371/journal.pone.0107018)
Supplement: Table S9 — Summary of results pertaining to covariates from the top occupancy models for the NS. (DOC) [file pone.0107018.s023.doc]

**Supporting Information**

**Table S9.** Summary of results pertaining to covariates from the top occupancy models for each species in the Neal Smith National Wildlife Refuge.

|  | | **Covariate (associated parameter)** | | | | |
| --- | --- | --- | --- | --- | --- | --- |
|  |  | hydroperiod^1^ (ψ) | % crops^2^ (ψ) | % not habitat^3^ (ψ) | mean patch size of habitat^4^ (ψ) | observer and  method^5^ (ρ) |
| **Species** | *Pseudacris maculata* (1^6^) |  |  |  |  |  |
| **Detail** | # models ≤ 5 ∆AIC^7^ | 0 | 0 | 0 | 0 | 1 |
|  | sum AIC weights | 0 | 0 | 0 | 0 | 1 |
|  | sign of coefficient | na | na | na | na | +- |
| **Species** | *Lithobates pipiens* (2^6^) |  |  |  |  |  |
| **Detail** | # models ≤ 5 ∆AIC^7^ | 0 | 0 | 0 | 0 | 1 |
|  | sum AIC weights | 0 | 0 | 0 | 0 | 0.420 |
|  | sign of coefficient | na | na | na | na | -- |

^1^ hydroperiod category for each site (ephemeral, semi-permanent, or permanent)

^2^ % of a 4-km buffer around each site that was cultivated cropland

^3^ % of a 4-km buffer around each site that that was not amphibian habitat

^4^ mean patch size of land-cover types that were not cultivated cropland, but were potential amphibian habitat, within a 4-km buffer around each site

^5^ experience level of the observer and sampling method used per site visit

^6^ total # of suitable models ≤ 5 ∆AIC (Akaike’s Information Criterion)

^7^ # of models that included covariate x (of the total # of models ≤ 5 ∆AIC)

na = not applicable
