# Supplementary material for: Indicators of the Statuses of Amphibian Populations and Their Potential for Exposure to Atrazine in Four Midwestern U.S. Conservation Areas
Source: PLoS One. 2014 Sep 12;9(9):e107018. doi: 10.1371/journal.pone.0107018 (PMC4162561; doi:10.1371/journal.pone.0107018)
Supplement: Table S13 — Triazine concentrations in amphibian breeding sites in the SCNSR, UMR, and VNP. (DOC) [file pone.0107018.s027.doc]

**Supporting Information**

**Table S13.** Triazine concentrations in water samples collected from amphibian breeding sites in the St. Croix National Scenic Riverway (SC), Voyageurs National Park (V), and the Upper Mississippi River National Wildlife and Fish Refuge (P).

| **Site** | **Date** | **Total triazines** | **Atrazine** | **Deethlyatrazine** | **Deethylcyanazine acid** | **ELISA atrazine** |
| --- | --- | --- | --- | --- | --- | --- |
| SC4DC1 | 6/5/2003 | ND | ND | ND | ND | ND |
| SC9DE2 | 6/5/2003 | 0.04 | 0.04 | ND | ND | ND |
| V2DF1 | 6/11/2003 | ND | ND | ND | ND | ND |
| V6DB1 | 6/12/2003 | ND | ND | ND | ND | ND |
| V1DB1 | 6/11/2003 | ND | ND | ND | ND | ND |
| P4DA1 | 5/28/2003 | 0.06 | 0.06 | ND | ND | 0.10 |
| P7DA2 | 5/28/2003 | 0.18 | 0.18 | ND | ND | 0.07 |
| P10DA1 | 5/29/2003 | 0.06 | 0.06 | ND | ND | 0.08 |
| P4DA3 | 7/6/2004 | 0.06 | 0.06 | ND | ND | 0.09 |
| P4DA3 | 6/20/2005 | 0.33 | 0.14 | 0.05 | ND | 0.43 |
| P4DC1 | 7/6/2004 | 1.17 | 0.44 | 0.09 | 0.26 | 0.65 |
| P4DC1 | 6/20/2005 | 0.72 | 0.22 | 0.07 | 0.24 | 0.31 |
| P8DA1 | 7/6/2004 | 0.64 | 0.11 | 0.03 | ND | 0.19 |
| P8DB1 | 7/6/2004 | 0.59 | 0.36 | 0.08 | ND | 0.63 |
| P8DB1 | 6/20/2005 | 0.57 | 0.19 | 0.06 | 0.14 | 0.43 |
| P7DA3 | 7/7/2004 | 6.23 | 4.36 | 0.86 | ND | 3.63 |
| P7DA3 | 6/23/2005 | 0.12 | 0.09 | 0.03 | ND | 0.09 |
| P10DD1 | 7/7/2004 | 0.47 | 0.13 | ND | ND | 0.16 |
| P10DD1 | 6/21/2005 | 0.50 | 0.17 | 0.06 | 0.11 | 0.26 |
| P10DA3 | 7/7/2004 | 0.72 | 0.37 | 0.09 | ND | 0.48 |
| P10DA3 | 6/21/2005 | 0.09 | 0.09 | ND | ND | 0.22 |
| P11DA5 | 7/8/2004 | 0.39 | 0.19 | 0.04 | ND | 0.24 |
| P11DA5 | 6/21/2005 | 0.30 | 0.10 | 0.04 | 0.08 | 0.29 |
| P13DA1 | 7/8/2004 | 1.09 | 0.53 | 0.11 | ND | 0.78 |
| P13DA1 | 6/22/2005 | 0.59 | 0.20 | 0.07 | 0.16 | 0.34 |
| P13DB3 | 7/8/2004 | 1.00 | 0.49 | 0.09 | ND | 0.64 |
| P13DB3 | 6/22/2005 | 0.14 | 0.08 | 0.06 | ND | 0.14 |
| P14DB1 | 7/9/2004 | 0.91 | 0.47 | 0.10 | ND | 0.74 |
| P14DB1 | 6/22/2005 | 0.42 | 0.19 | 0.07 | 0.13 | 0.25 |
| P14DC1 | 7/9/2004 | 1.18 | 0.42 | 0.23 | ND | 0.69 |
| P14DC1 | 6/22/2005 | 1.86 | 1.02 | 0.31 | ND | 2.25 |

Results are from analyses performed by USGS’s Organic Geochemistry Research Group in Lawrence, Kansas, using liquid chromatography/mass spectrometry (LCMS) except for the last column. The last column contains results for samples collected at the same location and time but analyzed at the USGS’s Upper Midwest Environmental Sciences Center via an enzyme-linked immunosorbent assay (ELISA) designed to detect atrazine. The ELISA also could react with other triazines in this list. All concentrations are µg/L. The detection limits were 0.025 and 0.50 µg/L for the LCMS and ELISA methods, respectively. ND = non-detect.
