# Supplementary material for: Indicators of the Statuses of Amphibian Populations and Their Potential for Exposure to Atrazine in Four Midwestern U.S. Conservation Areas
Source: PLoS One. 2014 Sep 12;9(9):e107018. doi: 10.1371/journal.pone.0107018 (PMC4162561; doi:10.1371/journal.pone.0107018)
Supplement: Table S14 — Additional triazine concentrations in amphibian breeding sites in the SCNSR, UMR, and VNP. (DOC) [file pone.0107018.s028.doc]

**Supporting Information**

**Table S14.** Additional triazine concentrations in the same water samples listed in Table S13 from the St. Croix National Scenic Riverway (SC), Voyageurs National Park (V), and the Upper Mississippi River National Wildlife and Fish Refuge (P).

| **Site** | **Date** | **Deisopropylatrazine** | **Deethyldeisopropylatrazine and Didealkyatrazine** | **Hydroxyatrazine** | **Propazine** | **Other triazines detected** |
| --- | --- | --- | --- | --- | --- | --- |
| SC4DC1 | 6/5/2003 | ND | ND | ND | ND |  |
| SC9DE2 | 6/5/2003 | ND | ND | ND | ND |  |
| V2DF1 | 6/11/2003 | ND | ND | ND | ND |  |
| V6DB1 | 6/12/2003 | ND | ND | ND | ND |  |
| V1DB1 | 6/11/2003 | ND | ND | ND | ND |  |
| P4DA1 | 5/28/2003 | ND | ND | ND | ND |  |
| P7DA2 | 5/28/2003 | ND | ND | ND | ND |  |
| P10DA1 | 5/29/2003 | ND | ND | ND | ND |  |
| P4DA3 | 7/6/2004 | ND | ND | ND | ND |  |
| P4DA3 | 6/20/2005 | 0.03 | 0.07 | ND | 0.04 |  |
| P4DC1 | 7/6/2004 | 0.06 | 0.12 | 0.18 | 0.03 |  |
| P4DC1 | 6/20/2005 | 0.04 | 0.08 | ND | 0.04 | Simazine (0.03) |
| P8DA1 | 7/6/2004 | ND | ND | 0.47 | ND | Deisopropylhydroxyatrazine (0.03) |
| P8DB1 | 7/6/2004 | 0.05 | 0.1 | ND | ND |  |
| P8DB1 | 6/20/2005 | 0.03 | 0.08 | ND | 0.04 | Simazine (0.03) |
| P7DA3 | 7/7/2004 | 0.33 | 0.31 | 0.31 | ND |  |
| P7DA3 | 6/23/2005 | ND | ND | ND | ND |  |
| P10DD1 | 7/7/2004 | ND | ND | 0.34 | ND |  |
| P10DD1 | 6/21/2005 | 0.04 | 0.08 | ND | 0.04 |  |
| P10DA3 | 7/7/2004 | 0.05 | 0.12 | 0.09 | ND |  |
| P10DA3 | 6/21/2005 | ND | ND | ND | ND |  |
| P11DA5 | 7/8/2004 | ND | ND | 0.1 | ND | Deisopropylhydroxyatrazine (0.06)  Simazine (0.03) |
| P11DA5 | 6/21/2005 | ND | 0.05 | ND | ND |  |
| P13DA1 | 7/8/2004 | ND | 0.1 | 0.35 | ND |  |
| P13DA1 | 6/22/2005 | 0.03 | 0.13 | ND | ND |  |
| P13DB3 | 7/8/2004 | 0.05 | 0.06 | 0.31 | ND |  |
| P13DB3 | 6/22/2005 | ND | ND | ND | ND |  |
| P14DB1 | 7/9/2004 | 0.06 | 0.07 | 0.21 | ND |  |
| P14DB1 | 6/22/2005 | 0.03 | ND | ND | ND |  |
| P14DC1 | 7/9/2004 | 0.08 | 0.25 | 0.2 | ND |  |
| P14DC1 | 6/22/2005 | 0.09 | 0.17 | 0.2 | 0.04 | Simazine (0.03) |

Results here are from analyses performed by USGS’s Organic Geochemistry Research Group in Lawrence, Kansas via liquid chromatography/mass spectrometry. All concentrations are in µg/L. The detection limit was 0.025 µg/L. ND = non-detect
