# Supplementary material for: Indicators of the Statuses of Amphibian Populations and Their Potential for Exposure to Atrazine in Four Midwestern U.S. Conservation Areas
Source: PLoS One. 2014 Sep 12;9(9):e107018. doi: 10.1371/journal.pone.0107018 (PMC4162561; doi:10.1371/journal.pone.0107018)
Supplement: Text S1 — Studies of amphibians and atrazine in the midwestern United States. (DOC) [file pone.0107018.s029.doc]

**Supporting Information**

**Text S1**

STUDIES OF AMPHIBIANS AND ATRAZINE IN THE MIDWESTERN UNITED STATES

Few field studies of amphibians and atrazine have been conducted in the midwestern United States. Among them, Reeder et al. [1] reported potential relationships between atrazine concentrations and intersexuality in cricket frogs (*Acris crepitans*) that were not statistically significant based upon their limited sample size. Hayes et al. [2] described gonadal abnormalities in northern leopard frogs (*Lithobates pipiens*) collected from seven of eight wetlands in parts of four states, including Iowa, that they characterized as uncontaminated or contaminated based upon records of local applications of atrazine. Rohr et al. [3] reported that atrazine concentrations in a set of Minnesota wetlands predicted slightly more than half the variation in abundance of parasitic larval trematodes in resident northern leopard frogs and the combined concentrations of atrazine and phosphate predicted 74% of this variation. These researchers did not report on the presence or occupancy of amphibian populations at their study sites relative to atrazine exposure or other aspects of agricultural production. Overall, baseline information needed to help understand the risks midwestern amphibian populations face living near corn production is limited.

**References**

1. Reeder AL, Foley GL, Nichols DK, Hansen LG, Wikoff B, Faeh S, Eisold J, Wheeler MB, Warner R, Murphy JE, Beasley VR (1998) Forms and prevalence of intersexuality and effects of environmental contaminants on sexuality in cricket frogs (*Acris crepitans*). Env Health Pers 106(5): 261–266.

2. Hayes T, Haston K, Tsui M, Hoang A, Haeffele C, Vonk A (2003) Atrazine-induced hermaphroditism at 0.1 ppb in American leopard frogs (*Rana pipiens*): Laboratory and field evidence. Environ Health Per 111(4): 568–575.

3. Rohr JR, Schotthoefer AM, Raffel TR, Carrick HJ, Halstead N, Hoverman JT, Johnson CM, Johnson LB, Lieske C, Piwoni MD, Schoff PK, Beasley VR (2008) Agrochemicals increase trematode infections in a declining amphibian species. Nature 455: 1235–1240.
