# Supplementary material for: Indicators of the Statuses of Amphibian Populations and Their Potential for Exposure to Atrazine in Four Midwestern U.S. Conservation Areas
Source: PLoS One. 2014 Sep 12;9(9):e107018. doi: 10.1371/journal.pone.0107018 (PMC4162561; doi:10.1371/journal.pone.0107018)
Supplement: Text S2 — Details on the process of selecting study wetlands. (DOC) [file pone.0107018.s030.doc]

**Supporting Information**

**Text** **S2**

DETAILS ON THE PROCESS OF SELECTING STUDY WETLANDS

We divided the entire NS into three approximately equal blocks north to south and selected study wetlands in each section (see next paragraph). We blocked the entire UMR according to the 14 pools created by the pre-existing lock-and-dam system on the Mississippi River, selected six of these pools randomly (4, 7, 8, 10, 13, and 14), and then selected study wetlands in each of the six pools. We divided the entire SCNSR into 12 approximately equal, contiguous, linear sections, randomly selected a subset of eight of these sections, and selected study wetlands in each section. Traversing the interior of VNP to access wetlands was more difficult than the other areas. Therefore, we limited the pool of potential study wetlands in VNP to those located within one kilometer of roads, trails, or boat access, laid our grid of cells over these areas, and selected study wetlands. We did not block this reduced area of VNP.

We randomly selected individual cells to sample within each study area from the cells available in each section or pool except for VNP. We discarded cells that contained greater than 50% private land because of reduced access or greater than 75% contiguous open water in the SCNSR and VNP because of reduced likelihood of breeding habitat. We confirmed that selected cells contained at least one wetland that qualified as potential breeding habitat for at least one amphibian species by first using global-positioning-system receivers on the ground to census each cell and locate all wetlands within it. We then evaluated whether each such wetland did or could support amphibian breeding and reproduction based upon size, hydroperiod, depth, vegetation, surrounding landscape, and personal experience. We repeated the process of identifying cells containing a minimum of one potential breeding site until we had at least five wetlands per block (NS), pool (UMR), or section (SCNSR), and 50 wetlands in total for VNP. Most wetlands we located qualified as potential amphibian breeding habitat. The individual wetlands we sampled in all management areas most typically were relatively small shallow lentic wetlands of various types that most amphibian species in this region often used for reproduction and other purposes. They ranged in size from approximately 0.1 to 25 ha, but most were smaller than 1 ha. Wetlands we sampled in the UMR were amphibian breeding sites slightly elevated above channels in the floodplain of the Mississippi River and often connected to the river during flooding.

Areas of inference

Based upon our site-selection process, all FWS- or NPS-managed lands within the recognized boundaries of the NS, the UMR, and the SCNSR were within our pre-determined areas of inference for these conservation areas. Our limited portions of VNP within one kilometer of roads, trails, or boat access comprised our area of inference for this park.
