# Supplementary material for: Indicators of the Statuses of Amphibian Populations and Their Potential for Exposure to Atrazine in Four Midwestern U.S. Conservation Areas
Source: PLoS One. 2014 Sep 12;9(9):e107018. doi: 10.1371/journal.pone.0107018 (PMC4162561; doi:10.1371/journal.pone.0107018)
Supplement: Text S3 — Details regarding how we measured land-cover variables for use as covariates on ψ in occupancy models. (DOC) [file pone.0107018.s031.doc]

**Supporting Information**

**Text** **S3**

DETAILS REGARDING HOW WE MEASURED LAND-COVER VARIABLES FOR USE AS COVARIATES ON Ψ IN OCCUPANCY MODELS

We used gridded land-cover data from the 2001 National Land Cover Database (version 2.0; [1]) to calculate the fraction of cropland and other land-cover types within site buffers in the NS, UMR, SCNSR, and most of VNP. We used gridded land-cover data from the 2000 edition of the Ontario Land Cover Data Base [2] to calculate these fractions for some sites in the northern portion of VNP that were within 4-km of Ontario; both land-cover products were derived from Landsat sensor data. The National Land Cover Database did not distinguish quarries and mines from areas of barren rock and sediment. We distinguished between quarries and mines via examinations of fine-scaled orthoimagery of the landscapes within our study areas.

We acquired digital line data for roads [3] and developed a gridded version of local road networks at 10-m cell resolution, a width typical of the roads in and around our study areas. We then merged this roads dataset with the National Land Cover Database, which we had re-sampled at 10-m cell resolution. Using this merged dataset enabled us to calculate patch sizes for land-cover types across the landscape based upon how roads also affected patch sizes. We used the FRAGSTATS spatial-pattern analysis program [4] to calculate the area-weighted average size of landscape patches not in cultivated cropland, urban/suburban, quarry, or road cover. The area-weighted mean was derived from the size of each patch multiplied by its proportional abundance in the landscape; large patches had more influence on the mean statistic than small patches. This metric yielded a landscape-centric perspective on the size of uninterrupted habitat an anuran likely would have encountered within a buffer. Note that in deriving this metric, we only used patches from the U.S. portion of the landscape for northern sites in VNP because the large expanse of open water in Rainy Lake, which straddles the international border, likely rendered the land patches in Canada inaccessible, and therefore irrelevant, to amphibian populations in VNP.

**References**

1. Homer C, Huang C, Yang L, Wylie B, Coan M (2004) Development of a 2001 national land cover database for the United States. Photogram Eng Rem Sens 70(7): 829–840.

2. Spectranalysis Inc (2004) Outline of production methodology and description of 27 land cover classes. In: Introduction to the Ontario Land Cover Data Base. 2nd ed. Unpublished report to Ontario Ministry of Natural Resources.

3. U.S. Census Bureau (2010) TIGER/Line 2010 Census. Census tract national-based data. Available: http://www.census.gov/geo/www/tiger. Accessed 24 September 2012.

4. McGarigal K, Cushman SA, Ene E (2012) FRAGSTATS v4: Spatial pattern analysis program for categorical and continuous maps. Computer software program available at http://www.umass.edu/landeco/research/fragstats/fragstats.html. Accessed 24 September 2012.
