# Supplementary material for: Indicators of the Statuses of Amphibian Populations and Their Potential for Exposure to Atrazine in Four Midwestern U.S. Conservation Areas
Source: PLoS One. 2014 Sep 12;9(9):e107018. doi: 10.1371/journal.pone.0107018 (PMC4162561; doi:10.1371/journal.pone.0107018)
Supplement: Text S4 — Occupancy estimates − outcomes specific to study areas. (DOC) [file pone.0107018.s032.doc]

**Supporting Information**

**Text S4**

OCCUPANCY ESTIMATES − OUTCOMES SPECIFIC TO STUDY AREAS

*Neal Smith National Wildlife Refuge*

Of the two species for which we modeled occupancy in the NS, estimates of ψ suggest *P. maculata* and *L. pipiens* occupied roughly 67–78% of potential sites (Table 3). Standard errors associated with those estimates indicate overlap in estimates across years. Estimates of ρ were relatively moderate (0.387–0.482) for both species (Table 3). None of the top models included covariates on ψ, and the null model (no covariates on any model parameter) had the smallest AIC value among the two top models for *L. pipiens* (Table S5).

*Upper Mississippi River*

We modeled occupancy for four species in the UMR. Estimates of ψ suggest *A. americanus* occupied roughly 10% of potential sites in the UMR and *H. chrysoscelis/versicolor* occupied roughly 30% or fewer of their potential sites (Table 4). Standard errors associated with estimates of ψ indicate overlap in estimates across years for both species, except the estimates for *H. chrysoscelis/versicolor* in 2002, which were greater than those for 2003–2005 (Table 4). Low estimates of ρ for *A. americanus* and *H. chyrsoscelis/versicolor* (0.070–0.148) indicate we had limited success detecting them during daytime surveys when present, especially *H. chrysoscelis/versicolor* (Table 4). The null model had the lowest AIC value among the top models for *A. americanus* and mean patch size of habitat was important for estimating ψ for *H. chyrsoscelis/versicolor* (Tables S6, S10).

In contrast, estimates of ψ for *L. clamitans* and *L. pipiens* suggest these two species occupied roughly 60−75% and 70−90%, respectively, of potential sites and estimates of ρ indicate we detected them at least twice as often as we did the previous two species (Table 4). As with *H. chyrsoscelis/versicolor*, estimates of ψ were higher for both species in 2002. Standard errors associated with estimates of ψ show overlap in estimates across years for both species, although less clearly for *L. clamitans in 2002* compared with the other years (Table 4). Hydroperiod and mean patch size of habitat, and to a lesser extent, % crops, were important for estimating ψ for *L. pipiens* (sum of AIC weights = 0.611, 0.624, and 0.371, respectively; Table S10).

*St. Croix National Scenic Riverway*

We modeled occupancy for seven species in the SCNSR. Estimates of ψ suggest *L. clamitans* occupied roughly 80–100% of potential sites, *L. sylvaticus* roughly 30–60%, and *L. pipiens and P. maculata* occupied roughly 40% and 30–40%, respectively. The remaining species occupied less than a third of their potential sites (Table 5). Standard errors associated with estimates of ψ indicate overlap in estimates across years for most species, except for the estimates for *L. clamitans* in 2002, which were higher than for the following years, and for *L. sylvaticus*, which were higher from 2003– 2005 than for 2002 (Table 5). Estimates of ρ were moderate (> 0.40) for *L. clamitans*, somewhat lower for *L. sylvaticus* and *L. pipiens*, and lower still for *A. americanus*, *P. crucifer*, *P. maculata*, and *L. septentrionalis* (Table 5). Among the generally small number of top models per species, mean patch size of habitat was important for estimating ψ for *P. crucifer* and *L. pipiens* (sum of AIC weights = 1; (Table S11). Hydroperiod was relatively low in importance for *L. sylvaticus* (0.296; Table S11). % crops was relatively low in importance for *L.pipiens* (0.270; Table S11).

*Voyageurs National Park*

We modeled occupancy for seven species in VNP. Estimates of ψ suggest *L*. *clamitans*, *L. pipiens*, *L. septentrionalis*, and *L. sylvaticus* occupied roughly 50–75% of their potential sites, depending upon the species (Table 6). Estimates of ρ for these species indicate we were successful detecting them roughly 30–45% of the time when they were present (Table 6). Estimates of ψ for *A. americanus*, *H. chrysoscelis*, and *P. crucif*er suggest these species occupied roughly 15–40% of their potential sites (except *A. americanus* in 2002 was higher at approximately 40–50% of sites), depending on the species, and estimates of ρ indicate we detected them roughly 15–25% of the time when present (Table 6). Standard errors associated with estimates of ψ show overlap in estimates across years for most species, except estimates for *A. americanus* stand out as higher in 2002 compared with 2003–2005 (Table 6). Among the limited number of top models per species, hydroperiod was relatively high in importance as a covariate for estimating ψ for *P. crucifer* (sum of AIC weights = 0.813; Table S12). Mean patch size of habitat was of limited importance for *A. americanus*, *H. chrysoscelis/versicolor*, and *L. pipiens* (sum of AIC weights = 0.362, 0.274 and 0.284, respectively; Table S12).
