# Supplementary material for: Indicators of the Statuses of Amphibian Populations and Their Potential for Exposure to Atrazine in Four Midwestern U.S. Conservation Areas
Source: PLoS One. 2014 Sep 12;9(9):e107018. doi: 10.1371/journal.pone.0107018 (PMC4162561; doi:10.1371/journal.pone.0107018)
Supplement: Text S5 — Relative difficulty of detecting different genera. (DOC) [file pone.0107018.s033.doc]

**Supporting Information**

**Text** **S5**

RELATIVE DIFFICULTY OF DETECTING DIFFERENT GENERA

Estimates of ψ and ρ generally were higher for *Lithobates* species than for non-*Lithobates* species across study areas, except for *P. maculata* in the NS and, to a lesser extent, *P. crucifer* in VNP (Tables 3–6). All life stages of the non-*Lithobates* anurans, except *A. americanus*, were smaller and more difficult to detect than their *Lithobates* counterparts. Also, as with *Lithobates* species, males typically called more often at night. These factors likely limited our ability to detect non-*Lithobates* species during daytime surveys, especially when they were low in abundance. In retrospect, we conceivably could have improved our parameter estimates for non-*Lithobates* species by increasing our sampling effort or if we had been able to sample these same sites at night via call surveys (not possible due to logistical constraints). For example, more recent data from call surveys we conducted using acoustic recorders in the SCNSR from 2008–2012, at a subset of the sites we report on here, indicate *P. crucifer* occupied approximately 90% of potential sites (unpublished results).
