# Supplementary material for: Indicators of the Statuses of Amphibian Populations and Their Potential for Exposure to Atrazine in Four Midwestern U.S. Conservation Areas
Source: PLoS One. 2014 Sep 12;9(9):e107018. doi: 10.1371/journal.pone.0107018 (PMC4162561; doi:10.1371/journal.pone.0107018)
Supplement: Text S6 — Results from exploratory sampling for phosphorous. (DOC) [file pone.0107018.s034.doc]

**Supporting Information**

**Text S6**

RESULTS FROM EXPLORATORY SAMPLING FOR PHOSPHOROUS

Our only insights into phosphate concentrations in our study areas were based upon exploratory surveys we conducted at wetlands in the UMR (three sites), the SCNSR (four sites), and VNP (three sites) in 2002, during which we collected grab samples from the water column at each site and sent them to the USGS National Water Quality Laboratory in Denver, Colorado, where they were analyzed for total phosphorous via a semi-automated colorimetry process. Results showed that median and first and third quartile concentrations of total phosphorous among these sites per area were 0.081, 0.047, and 0.083 for VNP; 0.13, 0.027, and 0.65 for the SCNSR; and 0.22, 0.16, and 0.26 mg/L for the UMR. This range of concentrations across these three study areas was similar to those Rohr et al. [1] reported for phosphates at their sites in Minnesota.

**References**

1. Rohr JR, Schotthoefer AM, Raffel TR, Carrick HJ, Halstead N, Hoverman JT, Johnson CM, Johnson LB, Lieske C, Piwoni MD, Schoff PK, Beasley VR (2008) Agrochemicals increase trematode infections in a declining amphibian species. Nature 455: 1235–1240.
