# Supplementary material for: Indicators of the Statuses of Amphibian Populations and Their Potential for Exposure to Atrazine in Four Midwestern U.S. Conservation Areas
Source: PLoS One. 2014 Sep 12;9(9):e107018. doi: 10.1371/journal.pone.0107018 (PMC4162561; doi:10.1371/journal.pone.0107018)
Supplement: Text S7 — Amphibian deformities reported relative to agricultural areas in the United States and Quebec. (DOC) [file pone.0107018.s035.doc]

**Supporting Information**

**Text** **S7**

PHYSICAL DEFORMITIES REPORTED RELATIVE TO AGRICULTURAL AREAS IN THE UNITED STATES AND QUEBEC

Our observed proportions of deformities across species were low across study areas, regardless of their proximity to corn production (Figs. 1, 2, S6) and the triazine concentrations we measured in wetlands (Fig. 5). Reeves et al. [1] reported an average of 2% abnormalities among 48,081 individual amphibian metamorphs sampled at 462 sites on 132 U.S. fish and wildlife refuges over a ten-year period and considerable variation in the deformities observed during repeated sampling of individual sites. Several of these refuges were in midwestern states near intensive corn production. Their observed deformity rates among several species for this subset of refuges were low as well, including the UMR, which they sampled at different locations than we did, but with similar results. They did observe high deformity rates at one refuge in northwestern Minnesota and one along the Mississippi River substantially south of the UMR and projected these as relative deformity hotspots that warranted further study.

Vandenlangenberg et al. [2] reported much higher rates of gross physical deformities in *L. pipiens* and other species from some sites they sampled in Minnesota that they categorized as affected (high deformity rates observed) compared with rates more similar to ours from sites they categorized as reference (lower deformity rates observed) and described their observed rates as much higher than those described in previous reports for the state. They did not report measuring any concentrations of agricultural chemicals, but sites in two of their study areas were near or on the edge of intensive corn production in south-central Minnesota.

Ouelett et al. [3] reported overall rates of gross physical deformities in anurans that were similar to ours (maximum of 7.4% deformed) across sites in Quebec, which they categorized qualitatively as agricultural or pesticide-free, but all the deformities they observed were in animals from agricultural sites. None of the *L. pipiens* they examined from either type of site were deformed. Ouelett et al. [3], Reeves et al. [1], Rohr et al. [4], and Vandenlangenberg et al. [2] did not report any data describing atrazine concentrations at collection sites or the population statuses of *L. pipiens* or other species they studied, preventing any comparisons with our triazine measurements, or estimates of presence or site occupancy, in relation to the proximity to intensive corn production.

**References**

1. Reeves MK, Medley KA, Pinkney AE, Holyoak M, Johnson PTJ, et al. (2013) Localized Hotspots Drive Continental Geography of Abnormal Amphibians on U.S. Wildlife Refuges. PLoS ONE 8(11): e77467. doi:10.1371/journal.pone.0077467.

2. Vandenlangenberg SM, Canfield JT, Magner JA (2003) A regional survey of malformed frogs in Minnesota (USA) (Minnesota malformed frogs). Environ Mon Assess 82: 45–61.

3. Ouellet M, Bonin J, Rodrigue J, DesGranges J-L, Lair S (1997) Hindlimb deformities (ectromelia, ectrodactyly) in free-living anurans from agricultural habitats. J Wild Dis 33(1): 95–104.

4. Rohr JR, Schotthoefer AM, Raffel TR, Carrick HJ, Halstead N, Hoverman JT, Johnson CM, Johnson LB, Lieske C, Piwoni MD, Schoff PK, Beasley VR (2008) Agrochemicals increase trematode infections in a declining amphibian species. Nature 455: 1235–1240.
