# Supplementary material for: Indicators of the Statuses of Amphibian Populations and Their Potential for Exposure to Atrazine in Four Midwestern U.S. Conservation Areas
Source: PLoS One. 2014 Sep 12;9(9):e107018. doi: 10.1371/journal.pone.0107018 (PMC4162561; doi:10.1371/journal.pone.0107018)
Supplement: Text S8 — Further discussion of triazine concentrations in the NS and the UMR. (DOC) [file pone.0107018.s036.doc]

**Supporting Information**

**Text** **S8**

FURTHER DISCUSSION OF TRIAZINE CONCENTRATIONS IN THE NS AND UMR

Although variable within and across 2004 and 2005, overall the majority of triazine concentrations we measured in samples from the main channel of the Mississippi River in the UMR overlapped with the majority of concentrations we measured in samples from nearby lentic amphibian breeding sites (Figs. 5, S8). In fact, a small number of breeding sites had higher concentrations than any main-channel sites did in both years (Figs. 5, S8). The amphibian breeding sites in the UMR were slightly elevated above the surface of the main channel when water levels were not at flood stage, but many were connected at the surface during high water. Such high water levels did not occur over our sampling time frame in 2005, but could have occurred between sampling events in 2004. Hydrologic linkages and toxicant transport likely were complex at and below the surface across the floodplain of the Mississippi River. Other factors contributed to this complexity, including atmospheric deposition of atrazine across the entire floodplain, which almost certainly occurred during heavy rains in May of 2004 in particular (Fig. S10), and spatial and temporal variability in groundwater discharge. Summer evapotranspiration rates in the floodplain’s forests hypothetically also could have reduced the surface water in amphibian breeding sites and concentrated toxicants. Our results do suggest, however, that triazine concentrations we measured in the Mississippi River’s main channel in the UMR might be relevant for estimating potential concentrations in the UMR’s usually lentic amphibian breeding sites.

In contrast to the large quantities of atrazine potentially transported hydrologically as well as atmospherically into the UMR, breeding wetlands in the NS were not linked hydrologically to any rivers or streams during this study. Thus, most of the triazines we detected in samples from NS breeding sites likely were applied as atrazine to fields of intensive corn production near (Fig. 1) this relatively small

refuge (2,172 ha), transported atmospherically, and deposited across the landscape either with or without leaching by rain. During 2004 and 2005, triazine concentrations in NS breeding wetlands were not dissimilar from concentrations in UMR breeding wetlands (Fig. 5), demonstrating how different combinations of landscape factors can result in similar potential exposure regimes. These similarities further suggest measurements of atrazine concentrations in rivers taken as part of water-quality monitoring programs might be useful for estimating atrazine concentrations in isolated palustrine wetlands in the region. Sampling across broader spatial and temporal scales under varying weather conditions would be necessary to understand any such relations further.
